# DeepCpG: accurate prediction of single-cell DNA methylation states using deep learning

**DOI:** 10.1186/s13059-017-1189-z

**Published:** 2017-04-11

**Authors:** Christof Angermueller, Heather J. Lee, Wolf Reik, Oliver Stegle

**Affiliations:** 1 European Molecular Biology Laboratory, European Bioinformatics Institute, Wellcome Genome Campus, Hinxton, Cambridge, CB10 1SD UK; 2 Epigenetics Programme, Babraham Institute, Cambridge, UK; 3 Wellcome Trust Sanger Institute, Wellcome Genome Campus, Hinxton, Cambridge, CB10 1SA UK

**Keywords:** Deep learning, Artificial neural network, Machine learning, Single-cell genomics, DNA methylation, Epigenetics

## Abstract

Recent technological advances have enabled DNA methylation to be assayed at single-cell resolution. However, current protocols are limited by incomplete CpG coverage and hence methods to predict missing methylation states are critical to enable genome-wide analyses. We report DeepCpG, a computational approach based on deep neural networks to predict methylation states in single cells. We evaluate DeepCpG on single-cell methylation data from five cell types generated using alternative sequencing protocols. DeepCpG yields substantially more accurate predictions than previous methods. Additionally, we show that the model parameters can be interpreted, thereby providing insights into how sequence composition affects methylation variability.

**Electronic supplementary material:**

The online version of this article (doi:10.1186/s13059-017-1189-z) contains supplementary material, which is available to authorized users.

## Background

DNA methylation is one of the most extensively studied epigenetic marks and is known to be implicated in a wide range of biological processes, including chromosome instability, X-chromosome inactivation, cell differentiation, cancer progression and gene regulation [1–4].

Well-established protocols exist for quantifying average DNA methylation levels in populations of cells. Recent technological advances have enabled profiling DNA methylation at single-cell resolution, either using genome-wide bisulfite sequencing (scBS-seq [5]) or reduced representation protocols (scRRBS-seq [6–8]). These protocols have already provided unprecedented insights into the regulation and the dynamics of DNA methylation in single cells [6, 9], and have uncovered new linkages between epigenetic and transcriptional heterogeneity [8, 10, 11].

**Fig. 1:**
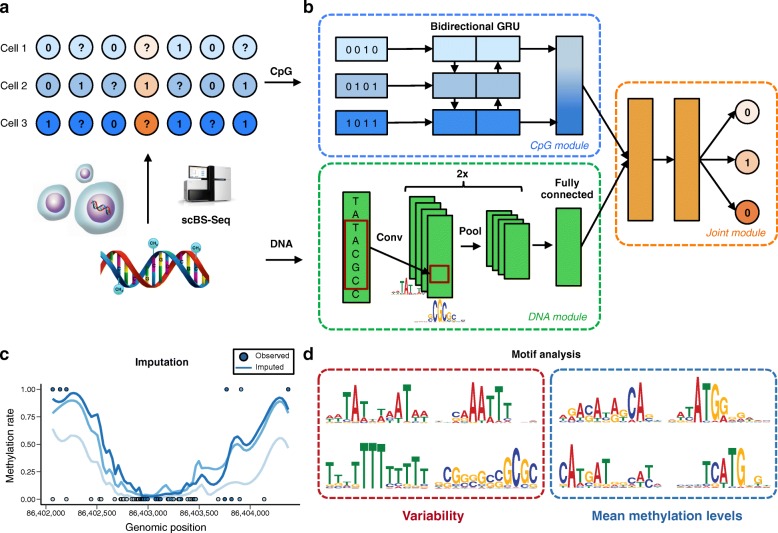
DeepCpG model training and applications. **a** Sparse single-cell CpG profiles as obtained from scBS-seq [5] or scRRBS-seq [6–8]. Methylated CpG sites are denoted by *ones*, un-methylated CpG sites by *zeros*, and *question marks* denote CpG sites with unknown methylation state (missing data). **b** Modular architecture of DeepCpG. The *DNA module* consists of two convolutional and pooling layers to identify predictive motifs from the local sequence context and one fully connected layer to model motif interactions. The *CpG module* scans the CpG neighbourhood of multiple cells (rows in **b**) using a bidirectional gated recurrent network (*GRU*) [36], yielding compressed features in a vector of constant size. The *Joint module* learns interactions between higher-level features derived from the DNA and CpG modules to predict methylation states in all cells. **c**, **d** The trained DeepCpG model can be used for different downstream analyses, including genome-wide imputation of missing CpG sites (**c**) and the discovery of DNA sequence motifs that are associated with DNA methylation levels or cell-to-cell variability (**d**)

Because of the small amounts of genomic DNA starting material per cell, single-cell methylation analyses are intrinsically limited by moderate CpG coverage (Fig. 1a; 20–40% for scBS-seq [5]; 1–10% for scRRBS-seq [6–8]). Consequently, a first critical step is to predict missing methylation states to enable genome-wide analyses. While methods exist for predicting average DNA methylation profiles in cell populations [12–16], these approaches do not account for cell-to-cell variability. Additionally, existing methods require a priori defined features and genome annotations, which are typically limited to a narrow set of cell types and conditions.

Here, we report DeepCpG, a computational method based on deep neural networks [17–19] for predicting single-cell methylation states and for modelling the sources of DNA methylation variability. DeepCpG leverages associations between DNA sequence patterns and methylation states as well as between neighbouring CpG sites, both within individual cells and across cells. Unlike previous methods [12, 13, 15, 20–23], our approach does not separate the extraction of informative features and model training. Instead, DeepCpG is based on a modular architecture and learns predictive DNA sequence and methylation patterns in a data-driven manner. We evaluated DeepCpG on mouse embryonic stem cells profiled using whole-genome single-cell methylation profiling (scBS-seq [5]), as well as on human and mouse cells profiled using a reduced representation protocol (scRRBS-seq [8]). Across all cell types, DeepCpG yielded substantially more accurate predictions of methylation states than previous approaches. Additionally, DeepCpG uncovered both previously known and de novo sequence motifs that are associated with methylation changes and methylation variability between cells.

## Results and discussion

DeepCpG is trained to predict binary CpG methylation states from local DNA sequence windows and observed neighbouring methylation states (Fig. 1a). A major feature of the model is its modular architecture, consisting of a *CpG module* to account for correlations between CpG sites within and across cells, a *DNA module* to detect informative sequence patterns, and a *Joint module* that integrates the evidence from the CpG and DNA module to predict methylation states at target CpG sites (Fig. 1b).

Briefly, the DNA and CpG modules were designed to specifically model each of these data modalities. The DNA module is based on a convolutional architecture, which has been successfully applied in different domains [24–27], including genomics [28–33]. The module takes DNA sequences in windows centred on target CpG sites as input, which are scanned for sequence motifs using convolutional filters, analogous to conventional position weight matrices [34, 35] (“Methods”). The CpG module is based on a bidirectional gated recurrent network [36], a sequential model that compresses patterns of neighbouring CpG states from a variable number of cells into a fixed-size feature vector (“Methods”). Finally, the Joint module learns interactions between output features of the DNA and CpG modules and predicts the methylation state at target sites in all cells using a multi-task architecture. The trained DeepCpG model can be used for different downstream analyses, including i) to impute low-coverage methylation profiles for sets of cells (Fig. 1c) and ii) to discover DNA sequence motifs that are associated with methylation states and cell-to-cell variability (Fig. 1d).

### Accurate prediction of single-cell methylation states

First, we assessed the ability of DeepCpG to predict single-cell methylation states and compared the model to existing imputation strategies for DNA methylation (“Methods”). As a baseline approach, we considered local averaging of the observed methylation states, either in 3-kbp windows centred on the target site of the same cell (*WinAvg*) or across cells at the target site (*CpGAvg*). Additionally, we compared DeepCpG to random forest classifiers [37] trained on individual cells using the DNA sequence information and neighbouring CpG states as input (*RF*). Finally, we evaluated a recently proposed random forest model to predict methylation rates for bulk ensembles of cells [12], which takes comprehensive DNA annotations into account, including genomic contexts, and tissue-specific regulatory annotations such as DNase1 hypersensitivity sites, histone modification marks, and transcription factor binding sites (*RF Zhang*). All methods were trained, selected and tested on distinct chromosomes via holdout validation (“Methods”). Since the proportion of methylated versus unmethylated CpG sites can be unbalanced in globally hypo- or hypermethylated cells, we used the area under the receiver operating characteristics curve (AUC) to quantify the prediction performance of different models. We have also considered a range of alternative metrics, including precision-recall curves, F1 score [38] and Matthews correlation coefficient [39], resulting in overall consistent conclusions (Additional file 1: Figures S1–S3; Additional file 2).

Initially, we applied all methods to 18 serum-cultured mouse embryonic stem cells (mESCs; average CpG coverage 17.7%; Additional file 1: Figure S4), profiled using whole-genome single-cell bisulfite sequencing (scBS-seq) [5].

**Fig. 2:**
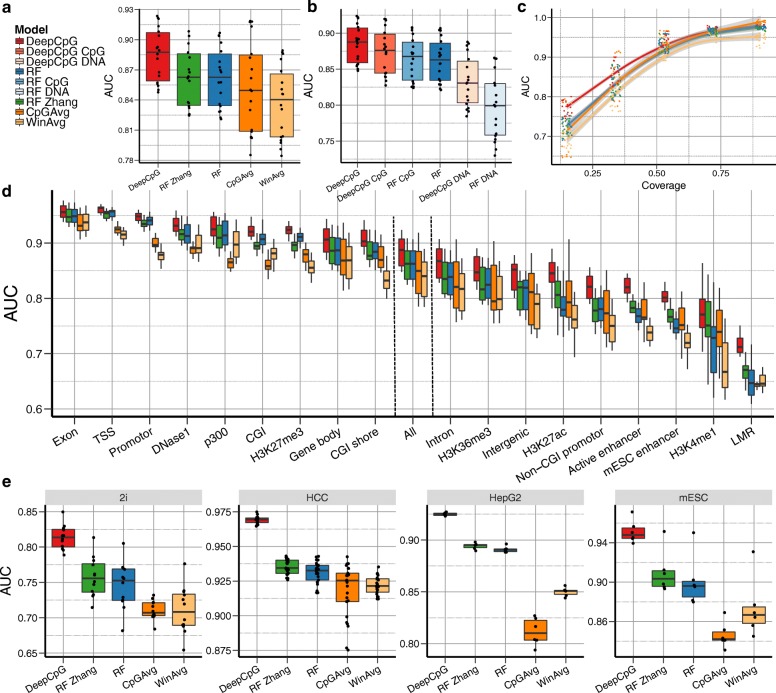
DeepCpG accurately predicts single-cell CpG methylation states. **a** Genome-wide prediction performance for imputing CpG sites in 18 serum-grown mouse embryonic stem cells (*mESCs*) profiled using scBS-seq [5]. Performance is measured by the area under the receiver-operating characteristic curve (*AUC*), using holdout validation. Considered were DeepCpG and random forest classifiers trained either using DNA sequence and CpG features (*RF*) or using additional annotations from corresponding cell types (*RF Zhang* [12]). Additionally, two baseline methods were considered, which estimate methylation states by averaging observed methylation states, either across consecutive 3-kbp regions within individual cells (*WinAvg* [5]) or across cells at a single CpG site (*CpGAvg*). **b** Performance breakdown of DeepCpG and RF, comparing the full models to models trained using either only methylation features (*DeepCpG CpG*, *RF CpG*) or only DNA features (*DeepCpG DNA*, *RF DNA*). **c** AUC of the methods as in (**a**) stratified by genomic contexts with increasing CpG coverage across cells. Trend lines were fit using local polynomial regression (*LOESS* [72]); *shaded areas* denote 95% confidence intervals. **d** AUC for alternative sequence contexts with *All* corresponding to genome-wide performance as in (**a**). **e** Genome-wide prediction performance on 12 2i-grown mESCs profiled using scBS-seq [5], as well as three cell types profiled using scRRBS-seq [8], including 25 human hepatocellular carcinoma cells (*HCC*), six HepG2 cells, and six additional mESCs. *CGI* CpG island, *LMR* low-methylated region, *TSS* transcription start site

DeepCpG yielded more accurate predictions than any of the alternative methods, both genome-wide and in different genomic contexts (Fig. 2). Notably, DeepCpG was consistently more accurate than RF Zhang, a model that relies on genomic annotations. These results indicate that DeepCpG can automatically learn higher-level features from the DNA sequence. To investigate this, we tested for associations between the activity of convolutional filters in the DNA module and known sequence annotations (“Methods”), finding both positive and negative correlations with several annotations, including DNase1 hypersensitive sites, histone modification marks, and CpG-rich genomic contexts (Additional file 1: Figure S5). The ability to extract higher-level features from the DNA sequence is particularly important for analysing single-cell datasets, where individual cells may be of different cell types and states, making it difficult to derive appropriate annotations.

To assess the relative importance of DNA sequence features compared to neighbouring CpG sites, we trained the same models, however, either exclusively using DNA sequence features (DeepCpG DNA, RF DNA) or neighbouring methylation states (DeepCpG CpG, RF CpG). Consistent with previous studies in bulk populations [12], methylation states were more predictive than DNA features, and models trained with both CpG and DNA features performed best (Fig. 2b). Notably, DeepCpG trained with CpG features alone outperformed random forest classifiers trained with both CpG and DNA features. A likely explanation for the accuracy of the CpG module is its recurrent network architecture, which enables the module to effectively transfer information from neighbouring CpG sites across different cells (Additional file 1: Figure S6).

The largest relative gains between RF and DeepCpG were observed when training both models with DNA sequence information only (AUC 0.83 versus 0.80; Fig. 2b). This demonstrates the strength of the DeepCpG DNA module to extract predictive sequence features from large DNA sequence windows of up to 1001 bp (Additional file 1: Figure S7a), which is particularly critical for accurate predictions from DNA in uncovered genomic regions, for example when using reduced representation sequencing data [6–8]. Consistent with this, the relative performance gain of DeepCpG compared to other methods was highest in contexts with low CpG coverage (Fig. 2c; Additional file 1: Figure S8).

Next, we explored the prediction performance of all models in different genomic contexts. In line with previous findings [12, 13], all models performed best in GC-rich contexts (Fig. 2d). However, DeepCpG offered most advantages in GC-poor genomic contexts, including non-CpG island promoters, enhancer regions, and histone modification marks (H3K4me1, H3K27ac)—contexts that are known to be associated with higher methylation variability between cells.

We also applied DeepCpG to 12 2i-cultured mESCs profiled using scBS-seq [5] and to data from three cell types profiled using scRRBS-seq [8], including 25 human hepatocellular carcinoma cells (HCCs), six human heptoplastoma-derived (HepG2) cells, and an additional set of six mESCs. Notably, in contrast to the serum cells, the human cell types are globally hypomethylated (Additional file 1: Figure S4). Across all cell types, DeepCpG yielded substantially more accurate predictions than alternative methods (Fig. 2e; Additional file 1: Figure S2), demonstrating the broad applicability of the model, including to hypo- and hypermethylated cells, as well as to data generated using different sequencing protocols.

### Estimation of the effect of DNA motifs and single-nucleotide mutations on methylation states

In addition to imputing missing methylation states, DeepCpG can be used to discover methylation-associated motifs and to investigate the effect of single-nucleotide mutations on CpG methylation.

**Fig. 3:**
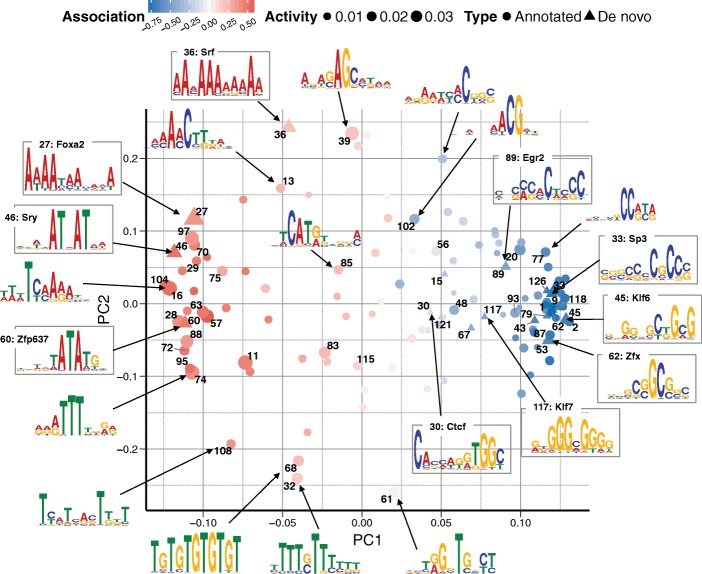
Discovered sequence motifs associated with DNA methylation. Clustering of 128 motifs discovered by DeepCpG. Shown are the first two principal components of the motif occurrence frequencies in sequence windows (activity). *Triangles* denote motifs with significant (FDR <0.05) similarity to annotated motifs in the CIS-BP [42] or UniPROPE [43] databases. Marker size indicates the average activity; the estimated motif effect on methylation level is shown by colour. Sequence logos are shown for representative motifs with larger effects, including ten annotated motifs

To explore this, we used the DeepCpG DNA module trained on serum mESCs and analysed the learnt filters of the first convolutional layer. These filters recognise DNA sequence motifs similarly to conventional position weight matrices and can be visualised as sequence logos (Fig. 3; Additional file 3). We considered two complementary metrics to assess the importance of the 128 motifs discovered by DeepCpG: i) their occurrence frequency in DNA sequence windows (activity), and ii) their estimated effect on single-cell methylation states (Additional file 1: Figure S9). To investigate the co-occurrence of motifs across sequence windows, we applied principal component analysis (Fig. 3) and hierarchical clustering (Additional file 1: Figures S10 and S11) to motif activities.

Motifs with similar nucleotide composition tended to co-occur in the same sequence windows, where two major motif clusters were associated with increased or decreased methylation levels (Fig. 3; Additional file 1: Figure S12). Consistent with previous findings [16, 40, 41], we observed that motifs associated with decreased methylation tended to be CG-rich and were most active in CG-rich promoter regions, transcription start sites, as well as in contexts with active promoter marks such as H3K4me3 and p300 sites (Additional file 1: Figure S11). Conversely, motifs associated with increased methylation levels tended to be AT rich and were most active in CG-poor genomic contexts (Additional file 1: Figure S11).

20 out of the 128 learned motifs significantly matched annotated motifs in the CIS-BP [42] and UniPROPE [43] databases (FDR <0.05). 17 of these annotated motifs were transcription factors with a known implication in DNA methylation [16, 44, 45], including CTCF [46], E2f [47] and members of the Sp/KLF family [48]—transcription factors and regulators of cell differentiation. 13 annotated motifs had been shown to interact with DNMT3a and DNMT3b [44], two major DNA methylation enzymes. Three annotated motifs have no clear associations with DNA methylation. These include Foxa2 [49, 50] and Srf [51, 52], which are implicated in cell differentiation and embryonic development, as well as Zfp637 [53, 54], a zinc finger protein that has recently been linked to spermatogenesis in mouse.

**Fig. 4:**
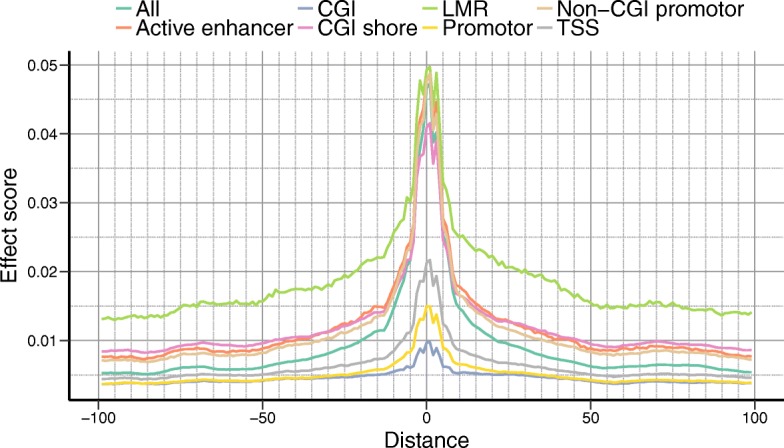
Effect of single-nucleotide mutations on DNA methylation. Average genome-wide effect of single-nucleotide mutations on DNA methylation estimated using DeepCpG, depending on the distance to the CpG site and genomic context. *CGI* CpG island, *LMR* low-methylated region, *TSS* transcription start site

The trained DeepCpG model can also be used to estimate the effect of single-nucleotide mutations on CpG methylation. We adapted a gradient-based approach [55] to estimate mutational effects in a computationally efficient manner, thereby greatly reducing the compute cost compared to previous methods [29, 30, 32] (“Methods”). As expected, mutations in the direct vicinity of the target CpG site had the largest effects (Fig. 4). Mutations in CG dense regions such as CpG islands or promoters tended to have smaller effects, suggesting that DNA methylation in these genomic contexts is more robust to single-nucleotide mutations. Globally, we observed a negative correlation between mutational effects and DNA sequence conservation (*P* < 1.0 × 10^−15^; Additional file 1: Figure S13), providing evidence that estimated single-nucleotide effects capture genuine effects. We further investigated mutational effects in HepG2 cells for 2379 methylation QTLs (mQTLs) [56], finding that known mQTL variants have significantly larger effects than matched random variants (*P* < 1.0 × 10^−15^, Wilcoxon rank sum test; Additional file 1: Figures S14 and S15).

### Discovery of DNA motifs that are associated with methylation variability

We further analysed the influence of motifs discovered by DeepCpG on methylation variability between cells.

To discern motifs that affect variability between cells from those that affect the average methylation level, we trained a second neural network. This network had the same architecture and in particular reused the motifs from the DNA module of DeepCpG; however, it was trained to jointly predict the variability across cells and the mean methylation level of each CpG site (“Methods”).

**Fig. 5:**
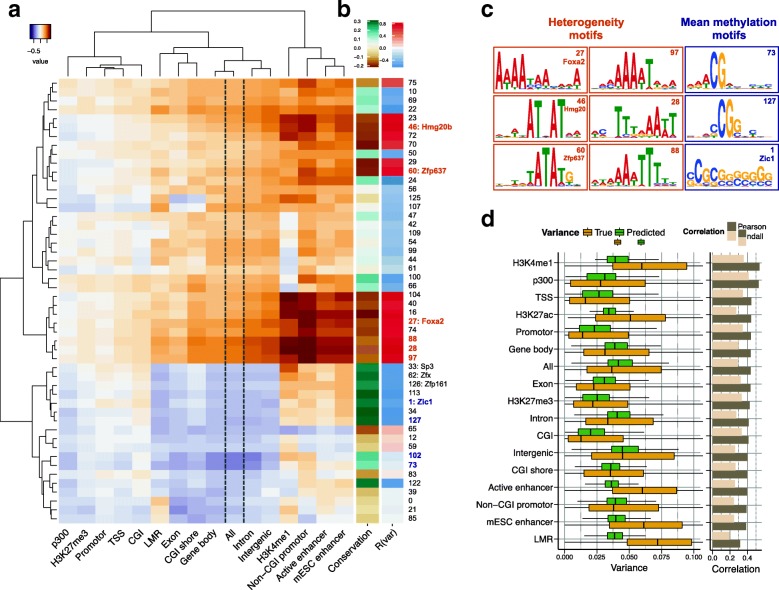
Prediction of methylation variability from local DNA sequence. **a** Difference of motif effect on cell-to-cell variability and methylation levels for different genomic contexts. Motifs associated with increased cell-to-cell variability are highlighted in *brown*; motifs that are primarily associated with changes in methylation level are shown in *purple*. **b** Genome-wide correlation coefficients between motif activity and DNA sequence conservation (*left*), as well as cell-to-cell variability (*right*). **c** Sequence logos for selected motifs identified in (**a**), which are highlighted with coloured text in (**b**). **d** Boxplots of the predicted and the observed cell-to-cell variability for different genomic contexts on held-out test chromosomes (*left*), alongside Pearson and Kendall correlation coefficients within contexts (*right*). *CGI* CpG island, *LMR* low-methylated region, *TSS* transcription start site

Notably, this model could predict both global changes in mean methylation levels (Pearson’s R = 0.80, MAD= 0.01, mean absolute deviation (*M﻿AD*); Additional file 1: Figure S16), as well as cell-to-cell variability (Pearson’s R = 0.44, MAD = 0.03; Fig. 5d; Kendall’s R = 0.29; Additional file 1: Figure S17).

There is an intrinsic relationship between mean methylation levels and cell-to-cell variance (Additional file 1: Figure S18); hence, the separation of the motif impact on mean methylation and methylation variance is partially confounded. To address this, we used a scoring approach that separates the effect of individual motifs on cell-to-cell variability and mean methylation levels (“Methods”). Briefly, we estimated the correlation between motif activities and predicted mean methylation levels as well as cell-to-cell variability and used the difference between the corresponding estimates to identify variance- and mean methylation-associated motifs. This analysis identified 22 motifs that were primarily associated with cell-to-cell variance (Fig. 5). These motifs tended to be active in CG-poor and active enhancer regions—sequence contexts with increased epigenetic variability between cells. Twelve of the identified motifs were AT-rich and associated with increased variability, including the differentiation factors Foxa2 [49, 50], Hmg20b [57] and Zfp637 [53, 54]. Notably, variance-increasing motifs were more frequent in unconserved regions such as active enhancers, in contrast to variance- decreasing motifs, which were enriched in evolutionarily conserved regions such as gene promoters (Fig. 5b; Additional file 1: Figure S19). Our analysis also revealed four motifs that were primarily associated with mean methylation levels, which were in contrast CG-rich and most active in conserved regions.

To explore whether the model predictions for variable sites are functionally relevant, we overlaid predictions with methylome–transcriptome linkages obtained using parallel single-cell methylation and transcriptome sequencing in the same cell type [10]. The rationale behind this approach is that regions with increased methylation variability are more likely to harbour associations with gene expression. Consistent with this hypothesis, we observed a weak but globally significant association (Pearson’s R = 0.11, *P* = 5.72 × 10^−16^; Additional file 1: Figure S20).

## Conclusions

Here we report DeepCpG, a computational approach based on convolutional neural networks for modelling low-coverage single-cell methylation data. Applying it to mouse and human cells, we show that DeepCpG accurately predicts missing methylation states and detects sequence motifs that are associated with changes in methylation levels and cell-to-cell variability.

We have demonstrated that our model enables accurate imputation of missing methylation states, thereby facilitating genome-wide downstream analyses. DeepCpG offers major advantages in shallow sequenced cells as well as in sparsely covered sequence contexts with increased methylation variability between cells. More accurate imputation methods may also help to reduce the required sequencing depth in single-cell bisulfite sequencing studies, thereby enabling the analysis of larger numbers of cells at reduced cost.

We have further shown that DeepCpG can be used to identify known and de novo sequence motifs that are predictive for DNA methylation levels or methylation variability and to estimate the effect of single-nucleotide mutations. Several of the motifs discovered by DeepCpG could be matched to known motifs that are implicated in the regulation of DNA methylation. The specific motifs that can be discovered are intrinsically limited to motifs that account for variations in a given dataset and hence depend on the considered cell type and latent factors that drive methylation variability. Computational approaches such as DeepCpG can also be used to discern pure epigenetic effects from variations that reflect DNA sequence changes. Although we have not considered this in our work, it would also be possible to use the model residuals for studying methylation variability that is independent of DNA sequence effects.

Finally, we have used additional data obtained from parallel methylation–transcriptome sequencing protocols [10] to annotate regions with increased methylation variability. An important area of future work will be to integrate multiple data modalities profiled in the same cells using parallel-profiling methods [8, 10], which are becoming increasingly available for different molecular layers.

## Methods

### DeepCpG model

DeepCpG consists of a *DNA module* to extract features from the DNA sequence, a *CpG module* to extract features from the CpG neighbourhood of all cells and a multi-task *Joint module* that integrates the evidence from both modules to predict the methylation state of target CpG sites for multiple cells.

#### DNA module

The DNA module is a convolutional neural network (CNN) with multiple convolutional and pooling layers and one fully connected hidden layer. CNNs are designed to extract features from high-dimensional inputs while keeping the number of model parameters tractable by applying a series of convolutional and pooling operations. Unless stated otherwise, the DNA module takes as input a 1001 bp long DNA sequence centred on a target CpG site *n*, which is represented as a binary matrix *s*_*n*_ by one-hot encoding the *D = 4* nucleotides as binary vectors A = [1, 0, 0, 0], T = [0, 1, 0, 0], G = [0, 0, 1, 0] and C = [0, 0, 0, 1]. The input matrix *s*_*n*_ is first transformed by a 1d-convolutional layer, which computes the *activations a*_*nfi*_ of multiple convolutional filters *f* at every position *i*:

1 $$ {a}_{n fi}=\mathrm{ReLU}\left({{\displaystyle {\sum}_{l=1}^L{\displaystyle {\sum}_{d=1}^D w}}}_{fld}{s}_{n, i+ l, d}\right). $$

Here, *w*_*f*_ are the parameters or *weights* of convolutional filter *f* of length *L*. These can be interpreted similarly to position weight matrices, which are matched against the input sequence *s*_*n*_ at every position *i* to recognise distinct motifs. The ReLU(*x*) = max(0, *x*) activation function sets negative values to zero, such that *a*_*nfi*_ corresponds to the evidence that the motif represented by *w*_*f*_ occurs at position *i*.

A pooling layer is used to summarise the activations of *P* adjacent neurons by their maximum value:

$$ {p}_{nf i}={ \max}_{\left| k\right|< P/2}\left({a}_{nf, i+ k}\right). $$

Non-overlapping pooling is applied with step size *P* to decrease the dimension of the input sequence and hence the number of model parameters. The DNA module has multiple pairs of convolutional-pooling layers to learn higher-level interactions between sequence motifs, which are followed by one final fully connected layer with a ReLU activation function. The number of convolutional-pooling layers was optimised on the validation set. For example, two layers were selected for models trained on serum, HCCs and mESCs and three layers for the 2i and HepG2 cells (Additional file 4).

#### CpG module

The CpG module consists of a non-linear embedding layer to model dependencies between CpG sites *within* cells, which is followed by a bidirectional gated recurrent network (GRU) [36] to model dependencies *between* cells. Inputs are 100*d* vectors *x*_1_, …, *x*_*T*_, where *x*_*t*_ represents the methylation state and distance of *K* = 25 CpG sites to the left and to the right of a target CpG site in cell *t*. Distances were transformed to relative ranges by dividing by the maximum genome-wide distance. The embedding layer is fully connected and transforms *x*_*t*_ into a 256*d* vector ${\overline{x}}_t$, which allows learning possible interactions between methylation states and distances within cell *t*:

$$ {\overline{x}}_t=\mathrm{ReLU}\left({W}_{\overline{x}} \cdot {x}_t+{b}_{\overline{x}}\right). $$

The sequence of vectors ${\overline{x}}_t$ are then fed into a bidirectional GRU [36], which is a variant of a recurrent neural network (RNN). RNNs have been successfully used for modelling long-range dependencies in natural language [58, 59], acoustic signals [60] and, more recently, genomic sequences [61, 62]. A GRU scans input sequence vectors ${\overline{x}}_1,\dots,\ {\overline{x}}_T$ from left to right and encodes them into fixed-size hidden state vectors *h*_1_, …, *h*_*T*_:

$$ {r}_t=\mathrm{sigmoid}\left({W}_{r\overline{x}}\cdot {\overline{x}}_t+{W}_{r h}\cdot {h}_{t-1}+{b}_r\right) $$

$$ {u}_t=\mathrm{sigmoid}\left({W}_{u\overline{x}}\cdot {\overline{x}}_t+{W}_{u h}\cdot {h}_{t-1}+{b}_u\right) $$

$$ {\overset{\sim }{h}}_t= \tanh \left({W}_{\overset{\sim }{h}\overline{x}} \cdot {\overline{x}}_t+{W}_{\overset{\sim }{h} h} \cdot \left({r}_t\odot {h}_{t-1}\right)+{b}_{\overset{\sim }{h}}\right) $$

$$ {h}_t=\left(1-{u}_t\right)\odot {h}_{t-1}+{u}_t\odot {\tilde{h}}_t. $$

The reset gate *r*_*t*_ and update gate *u*_*t*_ determine the relative weight of the previous hidden state *h*_*t*−1_ and the current input ${\overline{x}}_t$ for updating the current hidden state *h*_*t*_. The last hidden state *h*_*T*_ summarises the sequence as a fixed-size vector. Importantly, the set of parameters *W* and *b* are independent of the sequence length *T*, which allows summarising the methylation neighbourhood independent of the number of cells in the training dataset.

To encode cell-to-cell dependencies independently of the order of cells, the CpG module is based on a bidirectional GRU. It consists of a forward and backward GRU with 256*d* hidden state vectors *h*_*t*_, which scan the input sequence from the left and right, respectively. The last hidden state vector of the forward and backward GRU are concatenated into a 512*d* vector, which forms the output of the CpG module.

#### Joint module

The Joint module takes as input the concatenated last hidden vectors of the DNA and CpG module and models interactions between the extracted DNA sequence and CpG neighbourhood features via two fully connected hidden layers with 512 neurons and ReLU activation function. The output layer contains *T* sigmoid neurons to predict the methylation rate *ŷ*_*nt*_ ∈ [0; 1] of CpG site *n* in cell *t*:

$$ {\widehat{y}}_{nt}\left(\mathrm{x}\right)=\mathrm{sigmoid}\left(\mathrm{x}\right)=\left(\frac{1}{1+{\mathrm{e}}^{-\mathrm{x}}}\right). $$

#### Model training

Model parameters were learnt on the training set by minimizing the following loss function:

$$ L(w)={\mathrm{NLL}}_w\left(\widehat{y}, y\right)+{\lambda}_2{\left\Vert w\right\Vert}_2. $$

Here, the weight-decay hyper-parameter *λ*_2_ penalises large model weights quantified by the L2 norm, and NLL_*w*_(*ŷ*, *y*) denotes the negative log-likelihood, which measures how well the predicted methylation rates *ŷ*_*nt*_ fit to observed binary methylation states *y*_*nt*_ ∈ {0, 1}:

$$ {\mathrm{NLL}}_w\left(\widehat{y}, y\right)=-{\displaystyle {\sum}_{n=1}^N{\displaystyle {\sum}_{t=1}^T{o}_{n t}}}\left[{y}_{n t} \log \left({\widehat{y}}_{n t}\right)+\left(1-{y}_{n t}\right) \log \left(1-{\widehat{y}}_{n t}\right)\right]. $$

The binary indicator *o*_*nt*_ is set to one if the methylation state *y*_*nt*_ is observed for CpG site *n* in cell *t*, and zero otherwise. Dropout [63] with different dropout rates for the DNA, CpG and Joint module was used for additional regularization. Model parameters were initialised randomly following the approach in Glorot et al. [64]. The loss function was optimised by mini-batch stochastic gradient descent with a batch size of 128 and a global learning rate of 0.0001. The learning rate was adapted by Adam [65] and decayed by a factor of 0.95 after each epoch. Learning was terminated if the validation loss did not improve over ten consecutive epochs (early stopping). The DNA and CpG module were pre-trained independently to predict methylation from the DNA sequence (DeepCpG DNA) or the CpG neighbourhood (DeepCpG CpG). For training the Joint module, only the parameters of the hidden layers and the output layers were optimised, while keeping the parameters of the pre-trained DNA and CpG module fixed. Training DeepCpG on 18 serum mESCs using a single NVIDIA Tesla K20 GPU took approximately 24 h for the DNA module, 12 h for the CpG module and 4 h for the Joint module. Model hyper-parameters were optimised on the validation set by random sampling [66] (Additional file 4). DeepCpG is implemented in Python using Theano [67] 0.8.2 and Keras [68] 1.1.2.

### Prediction performance evaluation

#### Data pre-processing

We evaluated DeepCpG on different cell types profiled with scBS-seq [5] and scRRBS-seq [8].

scBS-seq-profiled cells contained 18 serum and 12 2i mESCs, which were pre-processed as described in Smallwood et al. [5], with reads mapped to the GRCm38 mouse genome. We excluded two serum cells (RSC27_4, RSC27_7) since their methylation pattern deviated strongly from the remaining serum cells.

scRRBS-seq-profiled cells were downloaded from the Gene Expression Omnibus (GEO; GSE65364) and contained 25 human HCCs, six human heptoplastoma-derived cells (HepG2) and six mESCs. Following Hou et al. [8], one HCC was excluded (Ca26) and we restricted the analysis to CpG sites that were covered by at least four reads. For HCCs and HepG2 cells, the position of CpG sites was lifted from GRCh37 to GRCh38, and for mESC cells from NCBIM37 to GRCm38, using the liftOver tool from the UCSC Genome Browser.

Binary CpG methylation states for both scBS-seq- and scRRBS-seq-profiled cells were obtained for CpG sites with mapped reads by defining sites with more methylated than un-methylated read counts as methylated, and un-methylated otherwise.

#### Holdout validation

For all prediction experiments and evaluations, we used chromosomes 1, 3, 5, 7, 9 and 11 as the training set, chromosomes 2, 4, 6, 8, 10 and 12 as the test set and the remaining chromosomes as the validation set (Additional file 5). For each cell type, models were fitted on the training set, hyper-parameters were optimised on the validation set and the final model performance and interpretations were exclusively reported on the test set. For computing binary evaluation metrics, such as accuracy, F1 score or MCC score, predicted methylation probabilities greater than 0.5 were rounded to one and set to zero otherwise. Genomic context annotations as shown in Fig. 2d are described in Additional file 6.

The prediction performance of DeepCpG was compared with random forest classifiers trained on each cell separately, using either features similar to DeepCpG (RF) or genome annotation marks as described in Zhang et al. [12] (RF Zhang). Additionally, we considered two baseline models, which estimate missing methylation states by averaging observed methylation states, either across consecutive 3-kbp regions within individual cells (WinAvg) or across cells at a single CpG site (CpGAvg).

#### Window averaging (WinAvg)

For window averaging, the methylation rate *ŷ*_*nt*_ of CpG site *n* and cell *t* was estimated as the mean of all observed CpG neighbours *y*_*n*+*k*,*t*_ in a window of length *W* = 3001 bp centred on the target CpG site *n*:

$$ {\widehat{y}}_{n t}={\mathrm{mean}}_{\left| k\right|<\frac{W}{2}, k\ne 0}\left({y}_{n+ k, t}\right). $$

*ŷ*_*nt*_ was set to the mean genome-wide methylation rate of cell *t* if no CpG neighbours were observed in the window.

#### CpG averaging (CpGAvg)

For CpG averaging, the methylation rate ${\widehat{y}}_{nt}$ of CpG site *n* in cell *t* was estimated as the average of the observed methylation states ${y}_{n{ t}^{\prime }}$ across all remaining cells *t*′≠ *t*:

$$ {\widehat{y}}_{n t}={\mathrm{mean}}_{t^{\prime}\ne t}\left({y}_{n{ t}^{\prime }}\right). $$

${\widehat{y}}_{nt}$ was set to the genome-wide average methylation rate of cell *t* if no methylation states were observed in any of the other cells.

#### Random forest models (RF, RF Zhang)

Features of the *RF* model were i) the methylation state and distance of 25 CpG sites to the left and right of the target site (100 features) and ii) *k*-mer frequencies in the *1001-bp genomic* sequence centred on the target site (256 features). The optimal parameter value for k (*k* = 4) was found using holdout validation (Additional file 1: Figure S21a).

The features for the RF Zhang model (Additional file 7) included i) the methylation state and distance of two CpG neighbours to the left and right of the target site (eight features), ii) annotated genomic contexts (23 features), iii) transcription factor binding sites (24 features), iv) histone modification marks (28 features) and v) DNaseI hypersensitivity sites (one feature). These features were obtained from the ChipBase database and UCSC Genome Browser for the GRCm37 mouse genome and mapped to the GRCm38 mouse genome using the liftOver tool from the UCSC Genome Browser.

We trained a separate random forest model for each individual cell, as a pooled multi-cell model performed worse (Additional file 1: Figure S21b). Hyper-parameters, including the number of trees and the tree depth, were optimised for each cell separately on the validation set by random sampling. Random forest models were implemented using the RandomForestClassifer class of the scikit-learn v0.17 Python package.

### Motif analysis

The motif analysis as presented in the main text was performed using the DNA module trained on serum mESCs. Motifs discovered for 2i cells, HCCs, HepG2 cells and mESCs are provided in Additional file 3. In the following, motifs are referred to filters of the first convolutional layer of the DNA module.

#### Visualization, motif comparison, Gene Ontology analysis

Filters of the convolutional layer of the DNA module were visualised by aligning sequence fragments that maximally activated them. Specifically, the activations of all filter neurons were computed for a set of sequences. For each sequence *s*_*n*_ and filter *f* of length *L*, sequence window *s*_*n*,*i* − *L/2*_, …, *s*_*n*,*i* + *L/2*_ were selected, if the activation *a*_*nfi*_ of filter *f* at position *i* (Eq. 1), was greater than 0.5 of the maximum activation of *f* over all sequences *n* and positions *i*, i.e. *a*_*nfi*_ > 0.5 max_*ni*_(*a*_*nfi*_). Selected sequence windows were aligned and visualised as sequence motifs using WebLogo [69] version 3.4.

Motifs discovered by DeepCpG were matched to annotated motifs in the *Mus musculus* CIS-BP [42] and UniPROBE [43] database (version 12.12, updated 14 Mar 2016), using Tomtom 4.11.1 from the MEME-Suite [70]. Matches at FDR <0.05 were considered as significant.

For Gene Ontology enrichment analysis, the web interface of the GOMo tool of MEME-Suite was used.

#### Quantification of motif importance

Two metrics were used to quantify the importance of filters: their activity (occurrence frequency) and their influence on model predictions.

Specifically, the activity of filter *f* for a set of sequences, e.g. within a certain genomic context, was computed as the average of mean sequence activities *ā*_*nf*_, where *ā*_*nf*_ denotes the weighted mean of activities *a*_*nfi*_ across all window positions *i* (Eq. 1). A linear weighting function was used to compute *ā*_*nf*_ that assigns the highest relative weight to the centre position.

The influence of filter *f* on the predicted methylation states *ŷ*_*nt*_ of cell *t* was computed as the Pearson correlation *r*_*ft*_ = cor_*n*_(*ā*_*nf*_, *ŷ*_*nt*_) over CpG sites *n*, and the mean influence *r*_*f*_ over all cells by averaging *r*_*ft*_.

#### Motif co-occurrence

The co-occurrence of filters was quantified using principal component analysis on the mean sequence activations *ā*_*nf*_ (Fig. 3) and pairwise correlations between mean sequence activations (Additional file 1: Figure S10).

#### Conservation analysis

The association between filter activities *ā*_*nf*_ and sequence conservation was assessed using Pearson correlation. *PhastCons* [71] conservation scores for the Glire subset (phastCons60wayGlire) were downloaded from the UCSC Web Browser and used to quantify sequence conservation.

### Effect of sequence and methylation state changes

We used gradient-based optimization as described in Simonyan et al. [55] to quantify the effect of changes in the input sequence *s*_*n*_ on predicted methylation rates *ŷ*_*nt*_(*s*_*n*_). Specifically, let *ŷ*_*n*_(*s*_*n*_) = mean_*t*_(*ŷ*_*nt*_(*s*_*n*_)) be the mean predicted methylation rate across cells *t*. Then the effect ${e}_{nid}^s$ of changing nucleotide *d* at position *i* was quantified as:

$$ {e}_{n id}^s=\frac{\Delta  {\hat{y}}_n\left({s}_n\right)}{\Delta {s}_{n id}}\ast \left(1-{s}_{n id}\right). $$

Here, the first term is the first-order gradient of *ŷ*_*n*_ with respect to *s*_*nid*_ and the second term sets the effect of wild-type nucleotides (*s*_*nid*_ = 1) to zero. The overall effect score ${e}_{ni}^s$ at position *i* was computed as the maximum absolute effect over all nucleotide changes, i.e. ${e}_{ni}^s={ \max}_d\left|{e}_{ni d}^s\right|$. The overall effect of changes at position *i* as shown in Fig. 3b was computed as the mean effect ${e}_i^s={\mathrm{mean}}_n\left({e}_{n i}^s\right)$ across all sequences *n*. For the mutation analysis shown in Additional file 1: Figure S13, ${e}_{ni}^s$ was correlated with *PhastCons* (phastCons60wayGlire) conservation scores. For quantifying the effect of methylation QTLs (mQTLs) as shown in Additional 1: Figure S14, we obtained mQTLs from the supplementary table of Kaplow et al. [56] and used the DeepCpG DNA module trained on HepG2 cells to compute effect scores for true mQTL variants. Non-mQTL variants were randomly sampled within the same sequence windows, distance-matched to real mQTL variants.

### Predicting cell-to-cell variability

For predicting cell-to-cell variability (variance) and mean methylation levels, we trained a second neural network with the same architecture as the DNA module, except for the output layer. Specifically, output neurons were replaced by neurons with a sigmoid activation function to predict for a single CpG site *n* both the mean methylation rate ${\widehat{m}}_{ns}$ and cell-to-cell variance ${\widehat{v}}_{ns}$ within a window of size *s* ∈ {1000, 2000, 3000, 4000, 5000} bp. Multiple window sizes were used to obtain predictions at different scales, using a multi-task architecture, thereby mitigating the uncertainty of mean and variance estimates in low-coverage regions. For training the resulting model, parameters were initialised with the corresponding parameters of the DNA module and fine-tuned, except for motif parameters of the convolutional layer. The training objective was:

$$ L( w)={\mathrm{MSE}}_w\left(\widehat{m}, m,\widehat{v}, v\right)+{\lambda}_2{\parallel w\parallel}_2, $$

where MSE the is mean squared error between model predictions and training labels:

$$ {\mathrm{MSE}}_w\left(\widehat{m}, m,\widehat{v}, v\right)={\displaystyle \sum_{n=1}^N}{\displaystyle \sum_{s=1}^S}{\left({m}_{n s}-{\widehat{m}}_{n s}\right)}^2+{\left({v}_{n s}-{\widehat{v}}_{n s}\right)}^2. $$

*m*_*ns*_ is the estimated mean methylation level for a window centred on target site *n* of a certain size indexed by *s*:

$$ {m}_{ns}=\frac{1}{T}{\displaystyle \sum_{t=1}^T}{m}_{ns t}. $$

Here, *m*_*nst*_ denotes the estimated mean methylation rate of cell *t* computed by averaging the binary methylation state *y*_*it*_ of all observed CpG sites *Y*_*nst*_ in window *s*:

$$ {m}_{nst}=\frac{1}{\left|{Y}_{nst}\right|}{\displaystyle \sum {{}_{i\in Y}}_{{}_{nst}}{y}_{i t}}, $$

where *v*_*ns*_ denotes the estimated cell-to-cell variance

$$ {v}_{ns}=\frac{1}{T}{\displaystyle {\sum}_{t=1}^T{\left({m}_{ns t}-{m}_{ns}\right)}^2}. $$

#### Identifying motifs associated with cell-to-cell variability

The influence ${r}_{fs}^v$ of filter *f* on cell-to-cell variability in widows of size *s* was computed as the Pearson correlation between mean sequence filter activities *ā*_*nf*_ and predicted variance levels ${\widehat{v}}_{ns}$ of sites *n*:

$$ {r}_{fs}^v = {\mathrm{cor}}_n\left({\overline{a}}_{n f},{\widehat{v}}_{n s}\right). $$

The influence ${r}_{fs}^m$ on predicted mean methylation levels ${\widehat{m}}_{ns}$ was computed analogously. The difference ${r}_{fs}^d=\left|{r}_{fs}^v\right|-\left|{r}_{fs}^m\right|$ between the absolute value of the influence on variance and mean methylation levels was used to identify motifs that were primarily associated with cell-to-cell variance (${r}_{fs}^d$ > 0.25) or with changes in mean methylation levels (${r}_{fs}^d$ < −0.25).

#### Functional validation of predicted variability

For functional validation, methylation–transcriptome linkages as reported in Angermueller et al. [10] were correlated with the predicted cell-to-cell variability. Specifically, let ${r}_{ij}^e$ be the linkage between expression levels of gene *i* and the mean methylation levels of an adjacent region *j* [10]. Then we correlated ${r}_{ij}^e$, which is the average predicted variability over all CpG sites within context *j*, and FDR adjusted *P* values over genes *i* and contexts *j*.

## Additional files

Additional file 1: Additional figures. (PDF 3485 kb)

Additional file 2: Prediction performance. Performance metrics for all cell types and models. (XLSX 3371 kb)

Additional file 3: Sequence motifs. HTML files with sequence logos and summary statistics for all cell types. (ZIP 19482 kb)

Additional file 4: DeepCpG hyper-parameters. Hyper-parameters of DeepCpG DNA, CpG and the Joint module. (XLSX 28 kb)

Additional file 5: Size of datasets. Number of training, validation and test samples for all cell types. (XLSX 36 kb)

Additional file 6: Genomic context. Description of genomic contexts. (XLSX 9 kb)

Additional file 7: Features used for training the RF Zhang model. (XLSX 30 kb)

## Abbreviations

AUC: Area under the receiver operating characteristic curve
bp: Base pair
CNN: Convolutional neural network
FDR: False discovery rate
GEO: Gene Expression Omnibus
GRU: Gated recurrent unit
HCC: Hepatocellular carcinoma cell
LMR: Low-methylated region
MAD: Mean absolute deviation
mESC: Mouse embryonic stem cell
mQTL: Methylation quantitative trait locus
RF: Random forest
RNN: Recurrent neural network
scBS-seq: single-cell bisulfite sequencing
scRNA-seq: Single-cell RNA sequencing
scRRBS-seq: Single-cell reduced representation bisulfite sequencing.
