# Erratum to: DeepCpG: accurate prediction of single-cell DNA methylation states using deep learning

**DOI:** 10.1186/s13059-017-1233-z

**Published:** 2017-05-12

**Authors:** Christof Angermueller, Heather J. Lee, Wolf Reik, Oliver Stegle

**Affiliations:** 1 European Molecular Biology Laboratory, European Bioinformatics Institute, Wellcome Genome Campus, Hinxton, Cambridge, CB10 1SD UK; 2 Epigenetics Programme, Babraham Institute, Cambridge, UK; 3 Wellcome Trust Sanger Institute, Wellcome Genome Campus, Hinxton, Cambridge, CB10 1SA UK

## Erratum

After publication of this article [1] it was noticed that the legend of Fig. 3 is incorrect. The circles should be labelled as ‘De novo’ and triangles as ‘Annotated’.

The original article has been updated.
