# Supplementary material for: DeepCpG: accurate prediction of single-cell DNA methylation states using deep learning
Source: Genome Biol. 2017 Apr 11;18:67. doi: 10.1186/s13059-017-1189-z (PMC5387360; doi:10.1186/s13059-017-1189-z)
Supplement: Supplementary file 1 — Additional figures. (PDF 3485 kb) [file 13059_2017_1189_MOESM1_ESM.pdf]

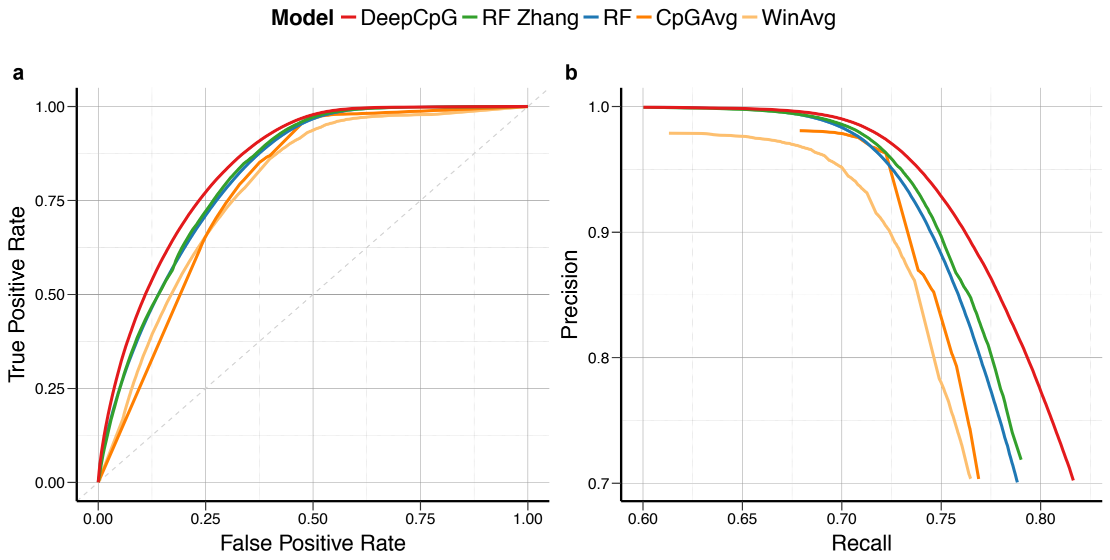


**Figure S1 | Receiver operating characteristic and precision recall curves for predicting DNA methylation states using alternative methods.** Receiver operating characterisic curve (**a**) and precision recall curve (**b**) for predicting methylation states in serum ESCs, analogous to results shown in main Figure 2. Considered were DeepCpG and random forest classifiers, either trained using similar features as DeepCpG (RF) or using additional DNA annotations (RF Zhang). Two baseline methods were considered, which estimate methylation states by averaging observed methylation states, either across consecutive 3-kbp regions within individual cells (WinAvg), or across cells at a single CpG site (CpGAvg). Curves represent the average performance across cells.

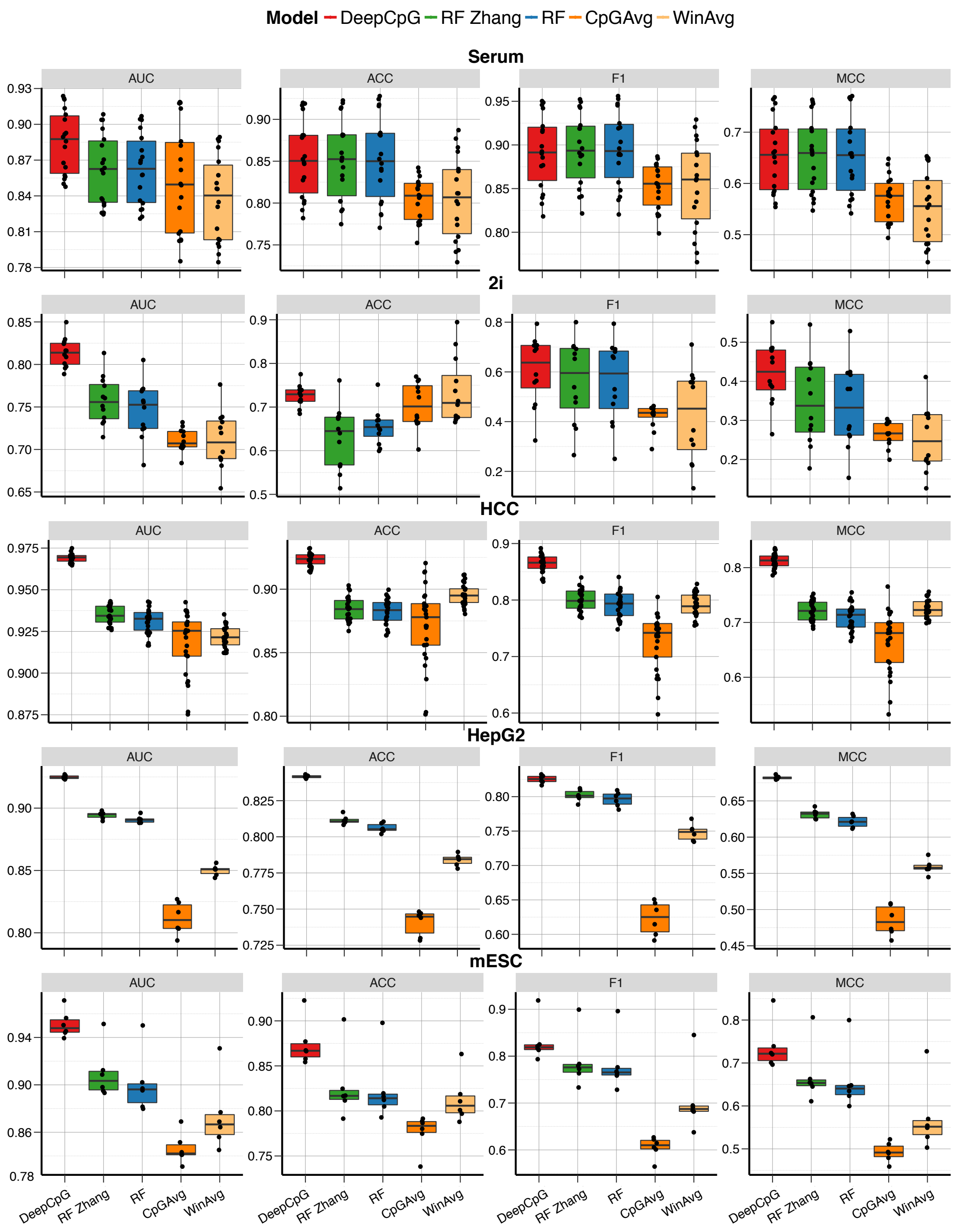


**Figure S2 | Prediction performance of alternative methods and for alternative metrics across five 5 datasets.** Test prediction performance metrics for 18 serum and 12 2i mouse ESCs profiled using scBS-seq (Smallwood et al., 2014), as well as for cells profiled using scRRBS-seq (Hou et al., 2016), including 25 human HCC cells, 6 HepG2 cells, and 6 additional mouse ESCs. Shown is the prediction performance for alternative methods, considering the area under receiver operating characteristic curve (AUC), accuracy (ACC), F1 score (F1), or Matthews correlation coefficient (MCC).

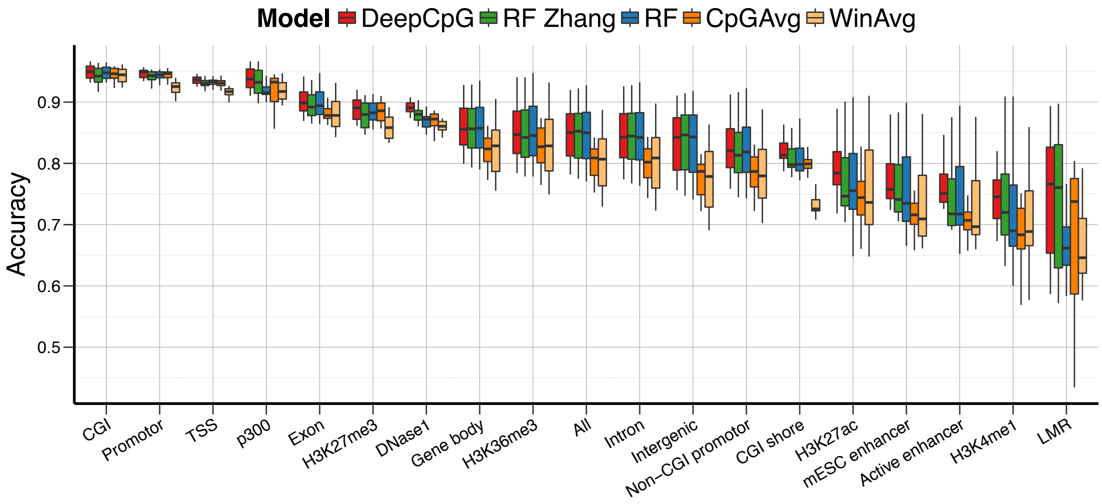


**Figure S3 | Accuracy of predictions from alternative methods in different genomic contexts for serum mESCs.** Test prediction accuracy for serum mouse ESCs stratified by different genomic contexts as in main Figure 2d.

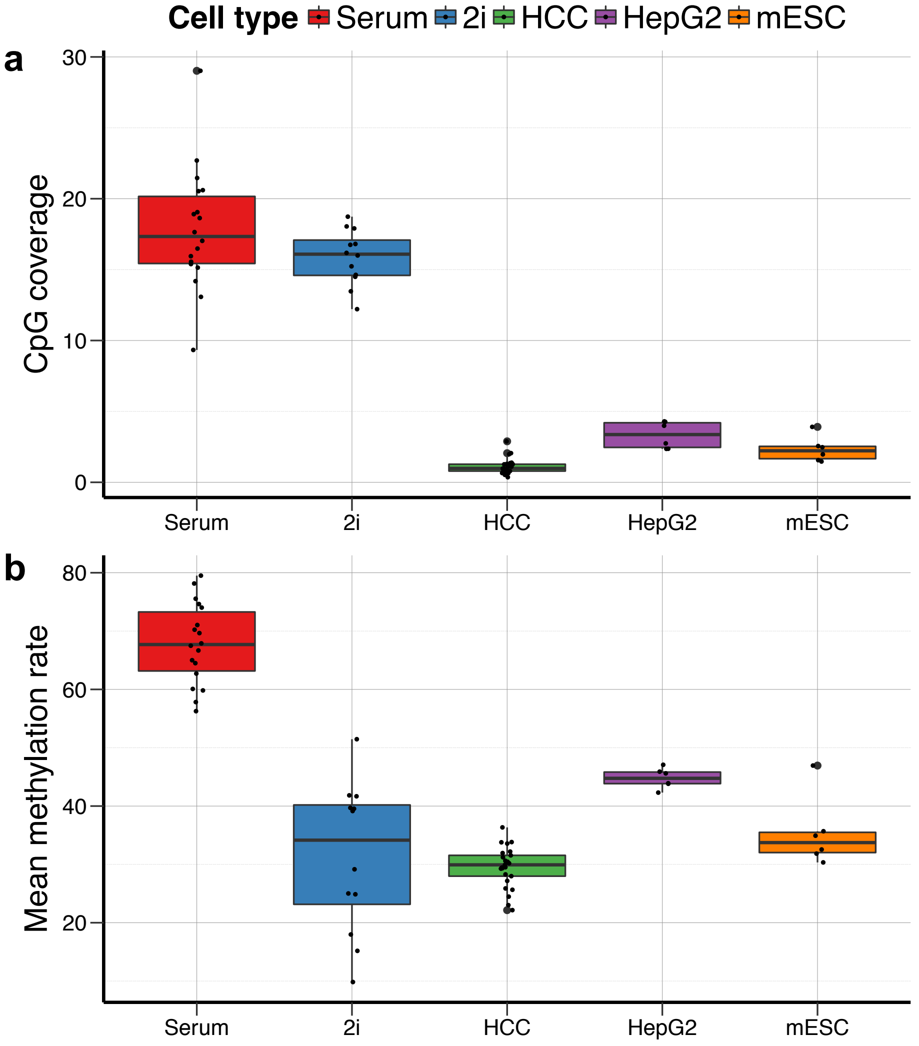


| | Serum | 2i | HCC | HepG2 | mESC |
|---|---|---|---|---|---|
| Number | 18 | 20 | 25 | 6 | 6 |
| CpG cov. | 17.8 | 15.9 | 1.1 | 3.3 | 2.3 |
| Meth. rate | 67.8 | 31.3 | 29.5 | 44.8 | 35.4 |

**Figure S4 | Quality metrics of cells profiled using scBS-seq and scRRBS-seq.** Genome wide CpG coverage (**a**) and mean methylation rate (**b**) of cells profiled using scBS-seq (Serum, 2i), and scRRBS-seq (HCC, HepG2, mESC).

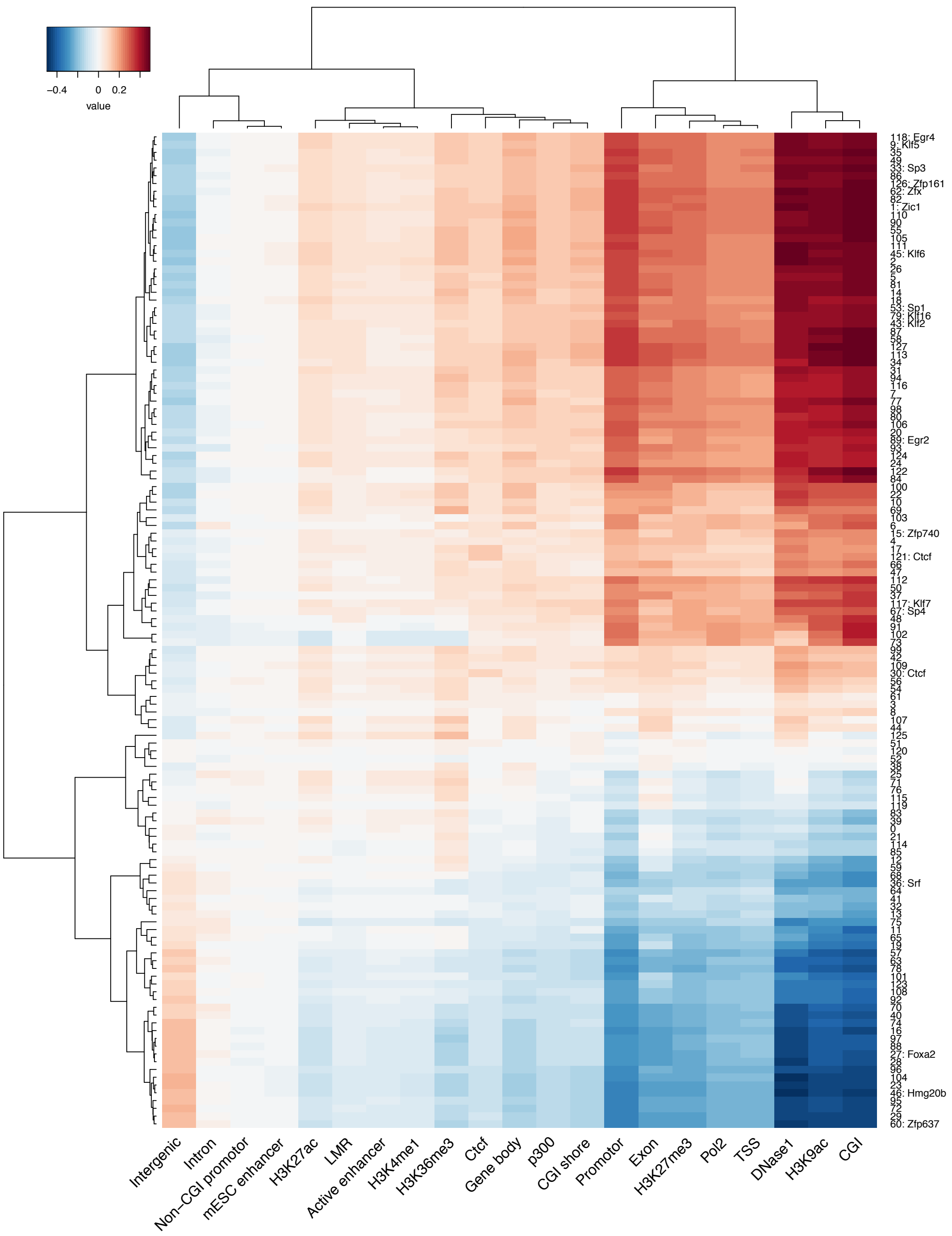


**Figure S5 | Correlation between filter activities and higher-level sequence features.** Spearman correlation between the activity of filters of the first convolutional layer of the DNA module (motifs) and higher-level sequence features considered in Zhang et al. Activities were strongly correlated with DNase1 hypersensitive sites, histone modification marks, and CpG dense genomic context. This indicates that DeepCpG is able to automatically learn higher-level features from the raw DNA sequence.

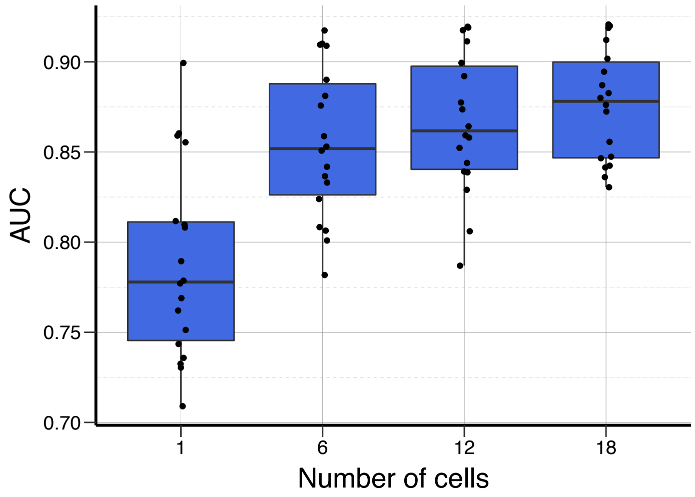


**Figure S6 | Prediction performance of the DeepCpG CpG module depending on the number of cells.** Test AUC for serum mouse ESCs, using an increasing number of cells as input for the DeepCpG CpG module.

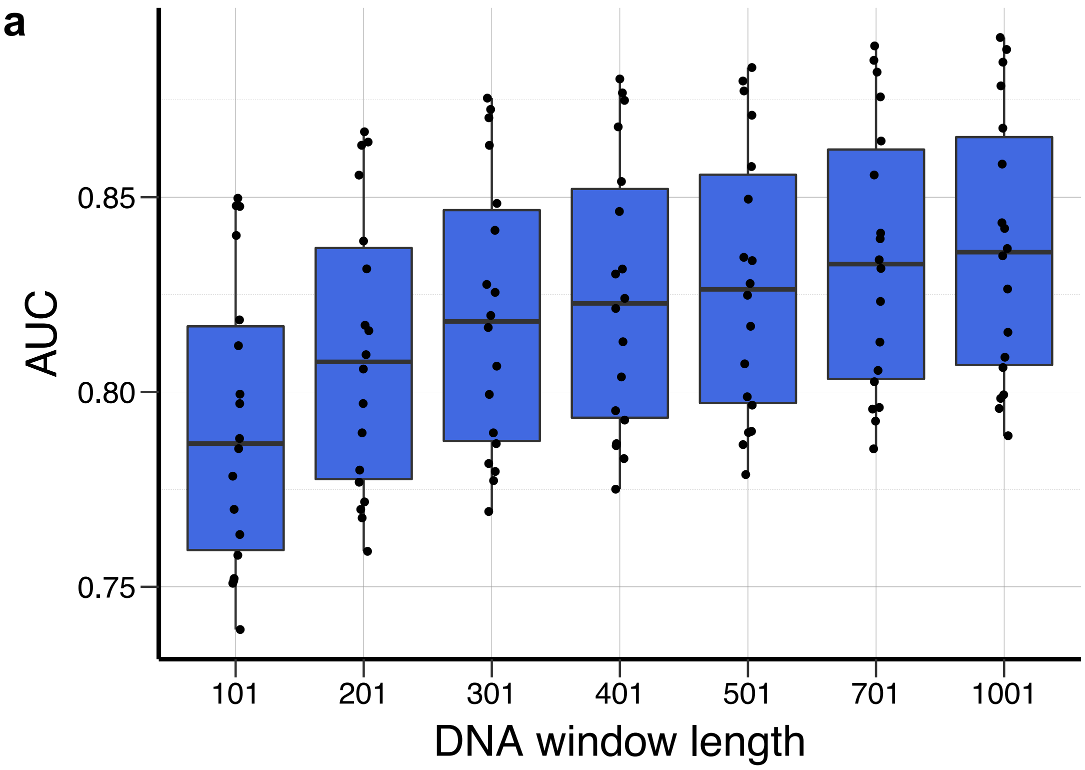


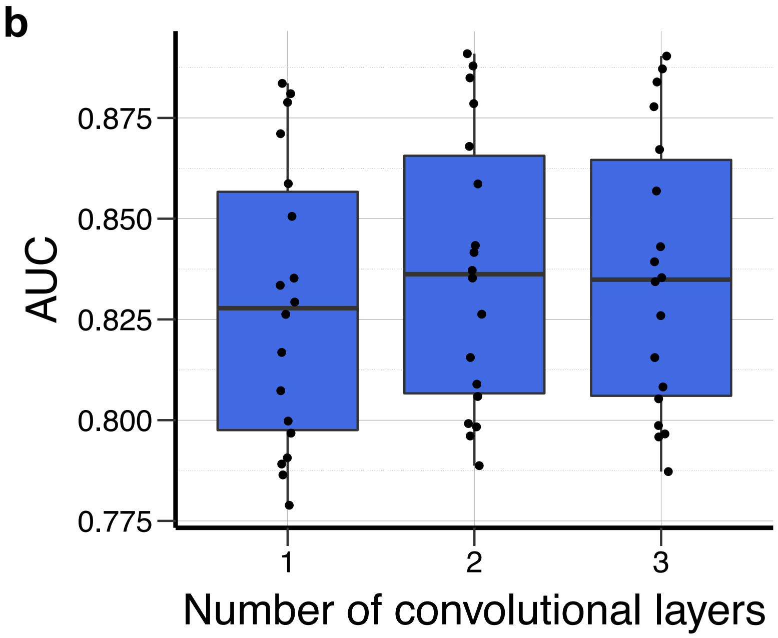


**Figure S7 | Prediction performance of the DeepCpG DNA module for DNA sequence windows of increasing length, and for architectures of different depth.** Test AUC for serum ESCs of the DeepCpG DNA module (DeepCpG DNA) analogous to main Figure 2b, depending on (**a**) the input DNA sequence window length (101 bp to 1001 bp), and (**b**) the number of convolutional layers in the model.

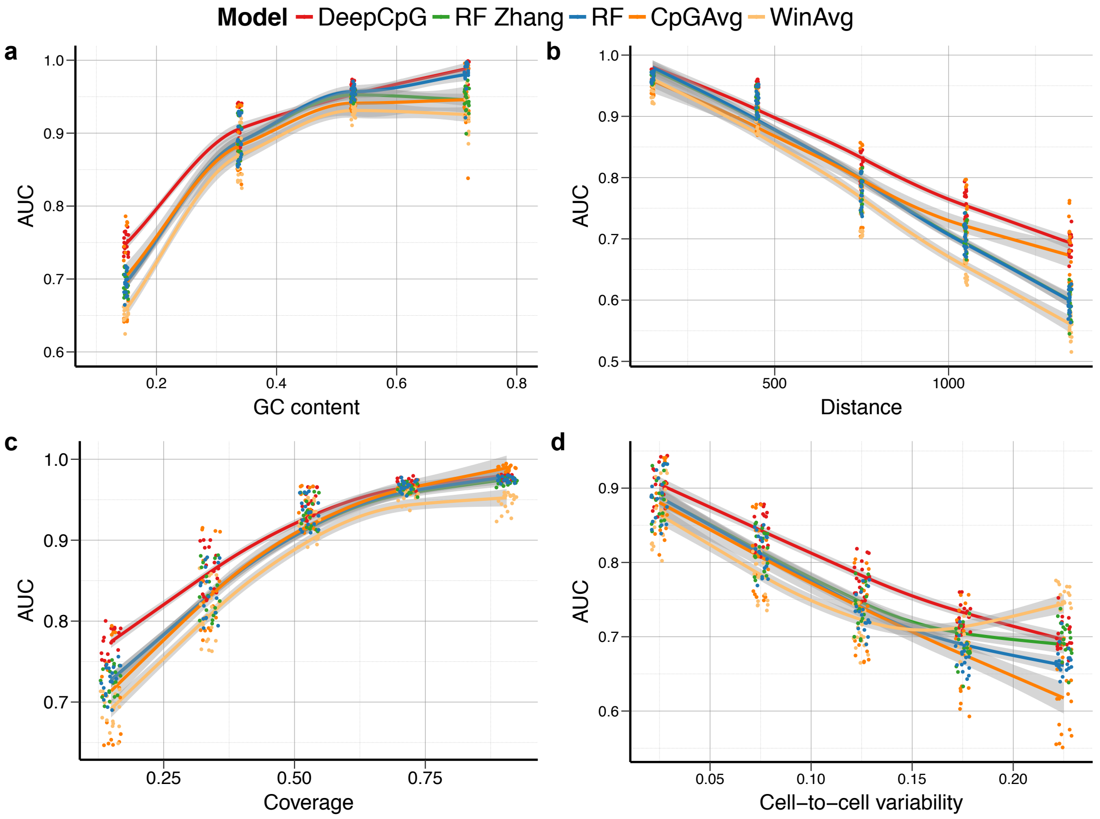


**Figure S 8 | Prediction performances stratified by different metrics.** Test AUC for serum mouse ESCs, considering alternative methods, stratified by (**a**) GC content, (**b**) distance to neighboring CpG sites, (**c**) fraction of cells by which the target CpG site is covered, (**d**) cell-to-cell variability within 3- kbp windows centered on the target CpG site. Trend lines were fit to observed coverage levels using local polynomial regression (LOESS), with shaded areas corresponding to 95% confidence intervals.

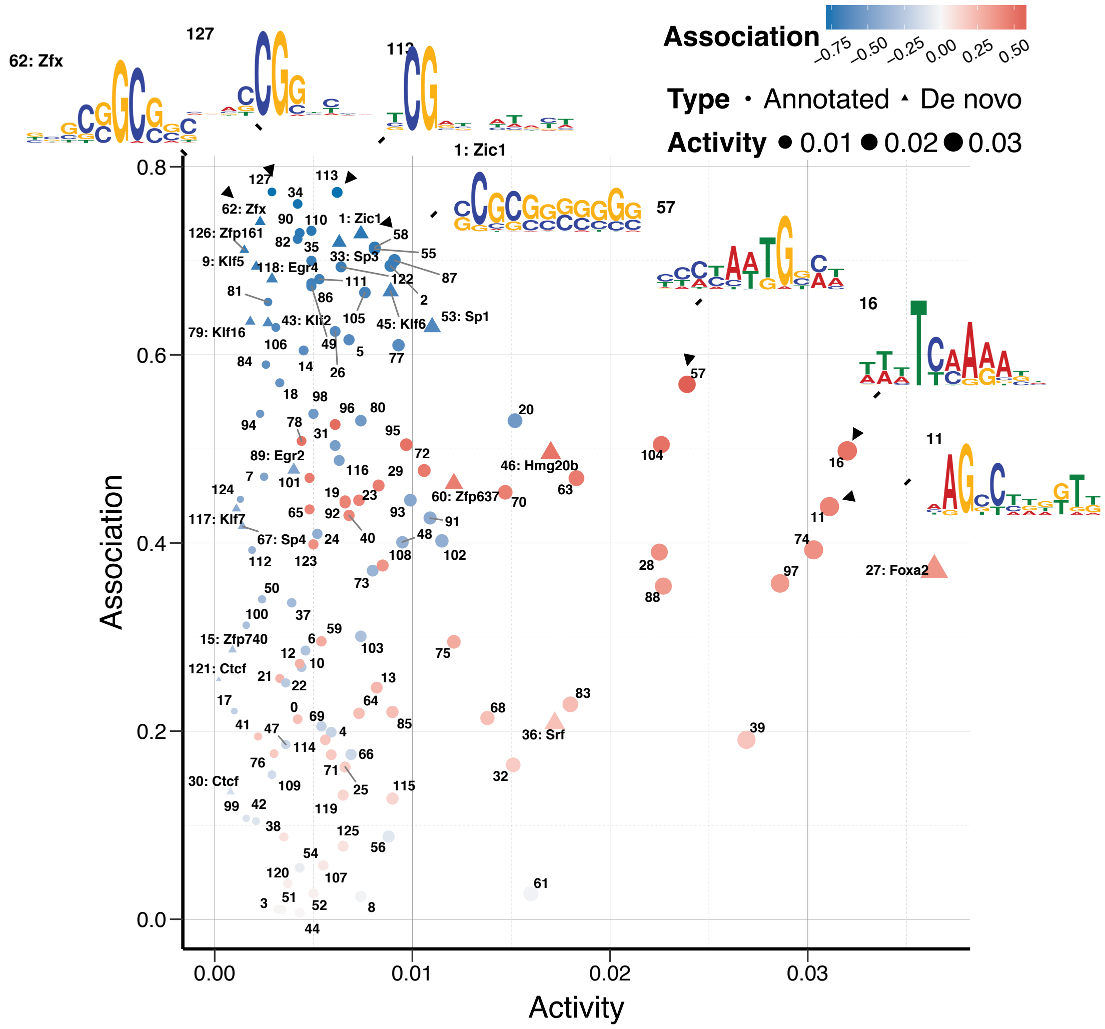


**Figure S9 | Importance of the DNA sequence motifs learnt by DeepCpG.** Average motif activity on the x-axis vs. the absolute estimated motif effect on methylation (association) on the y-axis. Marker size and colour correspond to the activity of motifs, and their association with methylation states, respectively. Triangles denote annotated motifs (matched to motif in CIS-BP or UniPROPE database; FDR<0.05); circles denote de novo motifs.

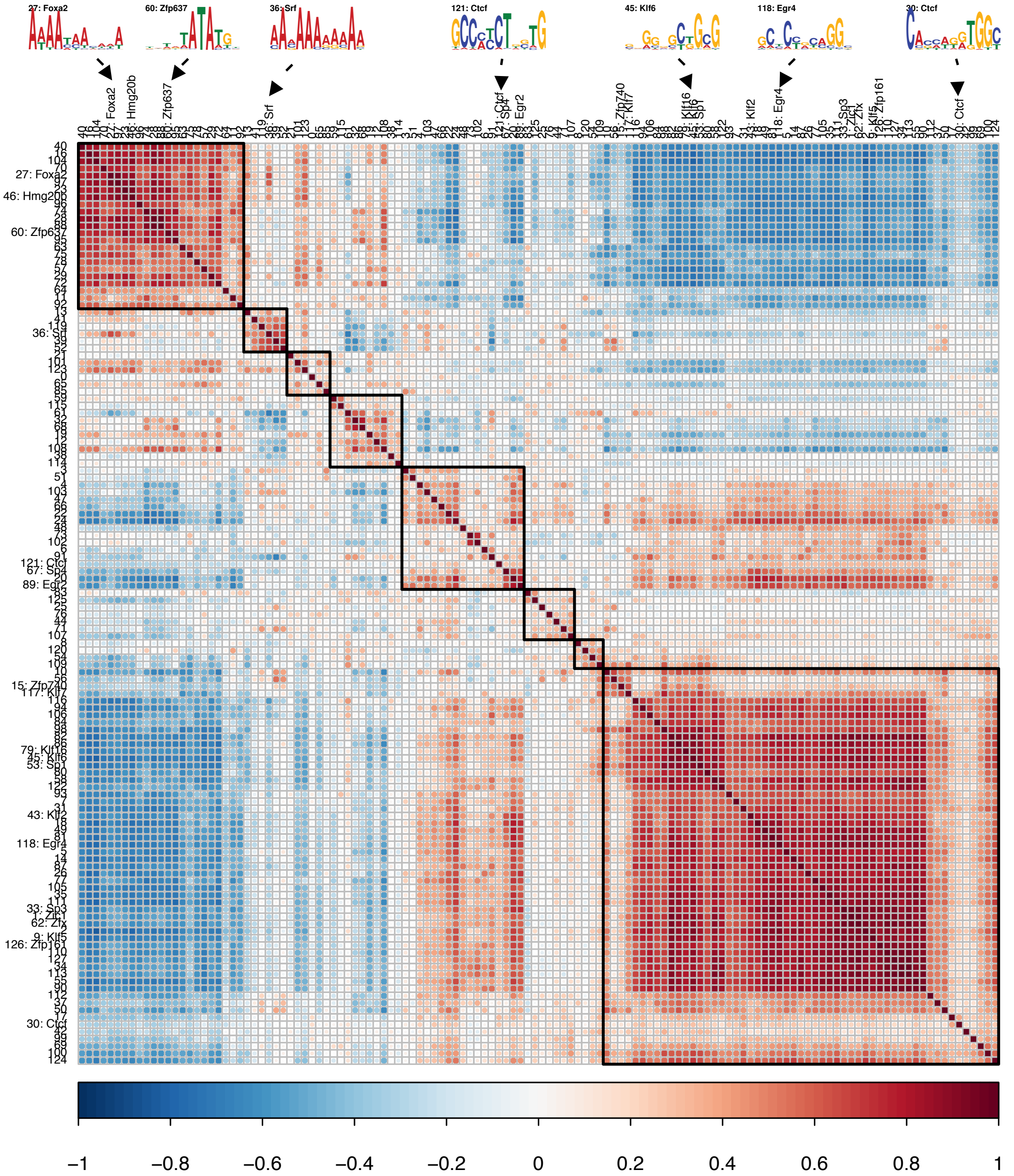


**Figure S10 | Correlation of DNA sequence motifs.** Pairwise pearson correlation coefficiens between motif activitities, showing clusters of motifs with similar nucleotide composition.

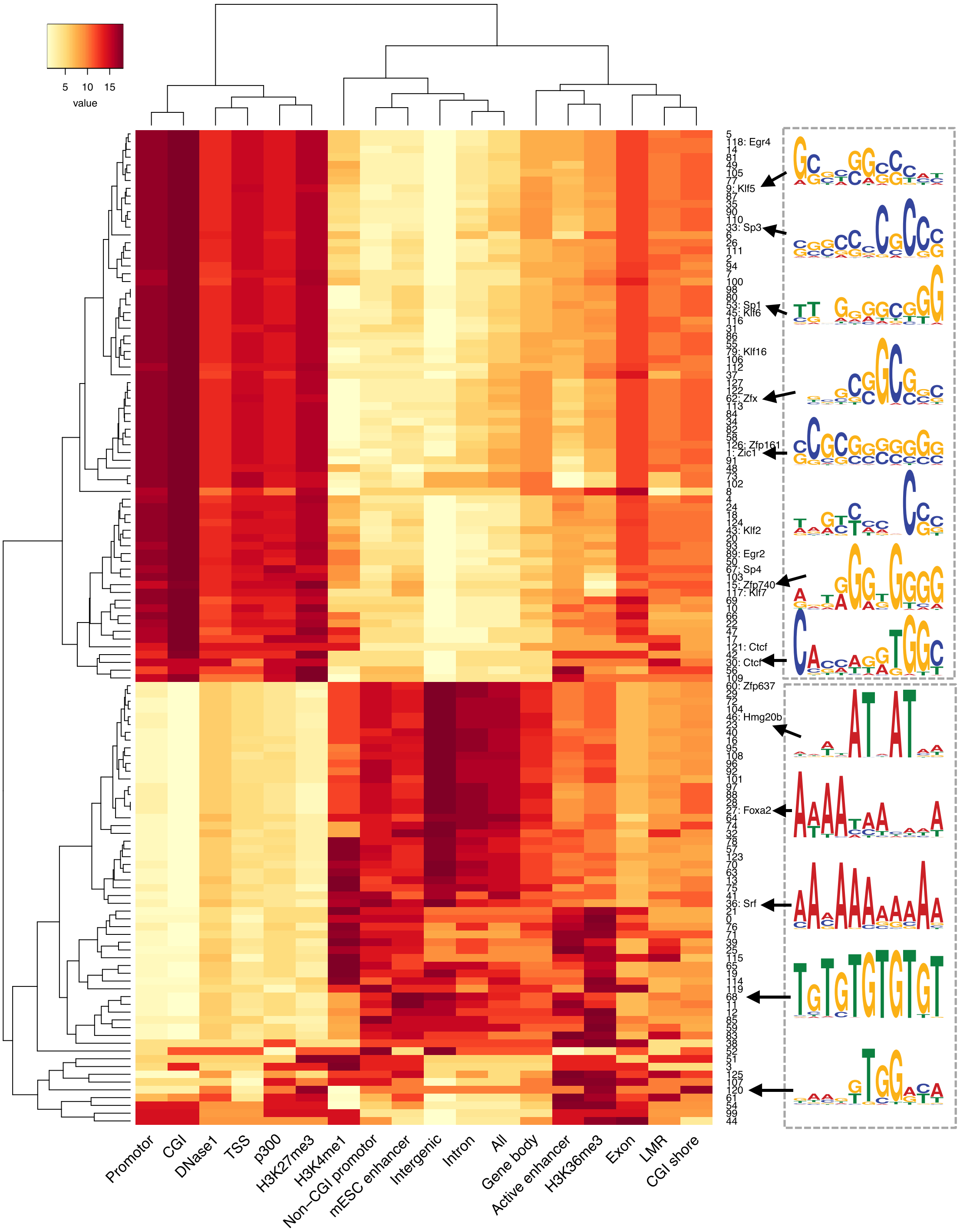


**Figure S11|Activity of DNA sequence motifs in genomic contexts.** Rank of motif activities in different genomic contexts on test chromosomes. The highest activity of CG rich motifs is observed in GC-rich regions such as promotors and CpG islands (CGI). Conversely, the highest activity of AT rich motifs is observed in GC-poor regions such as intergenic or active enhancer regions.

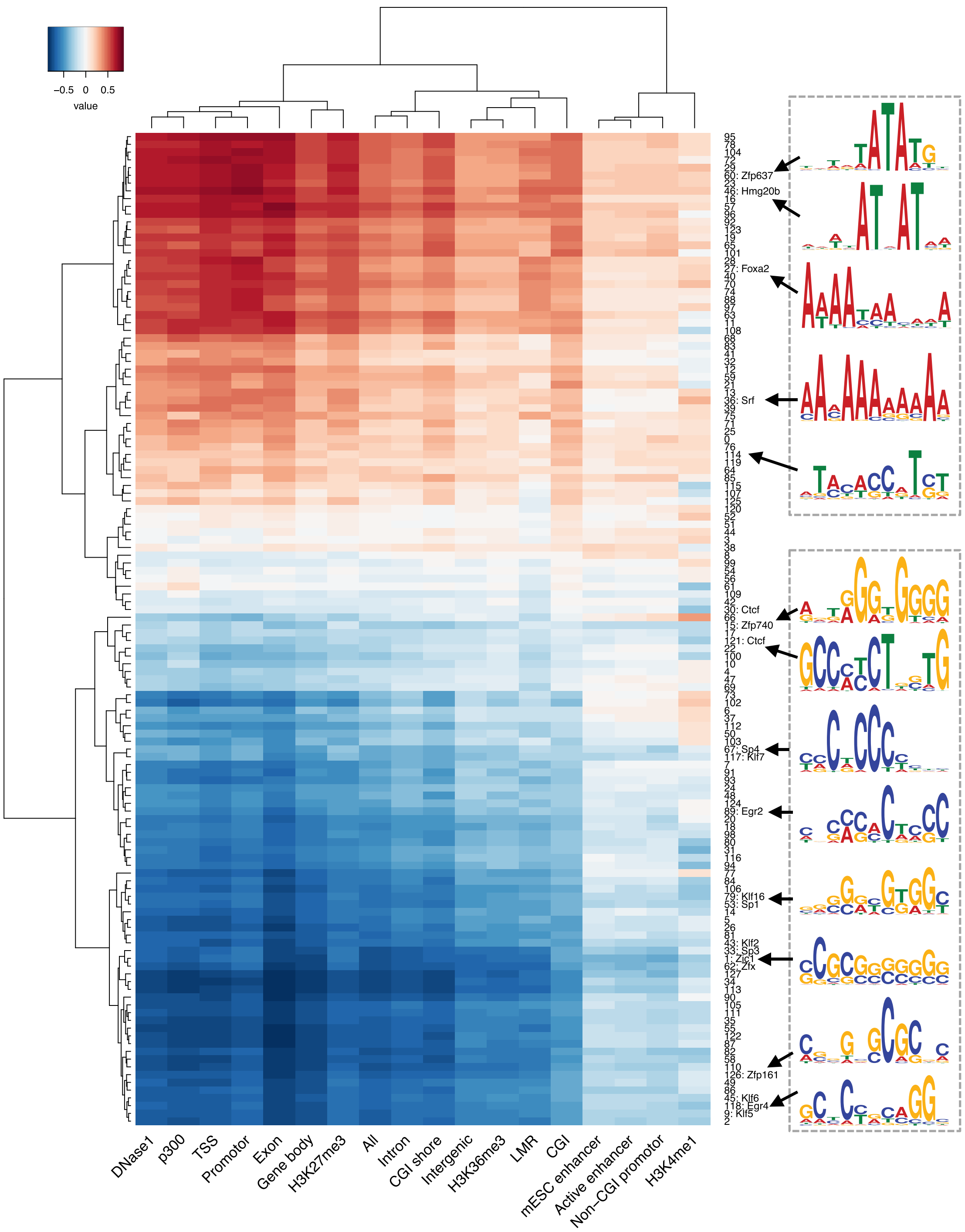


**Figure S12 | Effect of DNA sequence motifs on methylation.** Effect of discovered motifs on CpG methylation in different genomic contexts on test chromosomes, quantified by Pearson's correlation between motif activities and predicted methylation levels (**Methods**). Motifs cluster into CG-rich, methylation-decreasing motifs, and AT-rich methylation-increasing motifs.

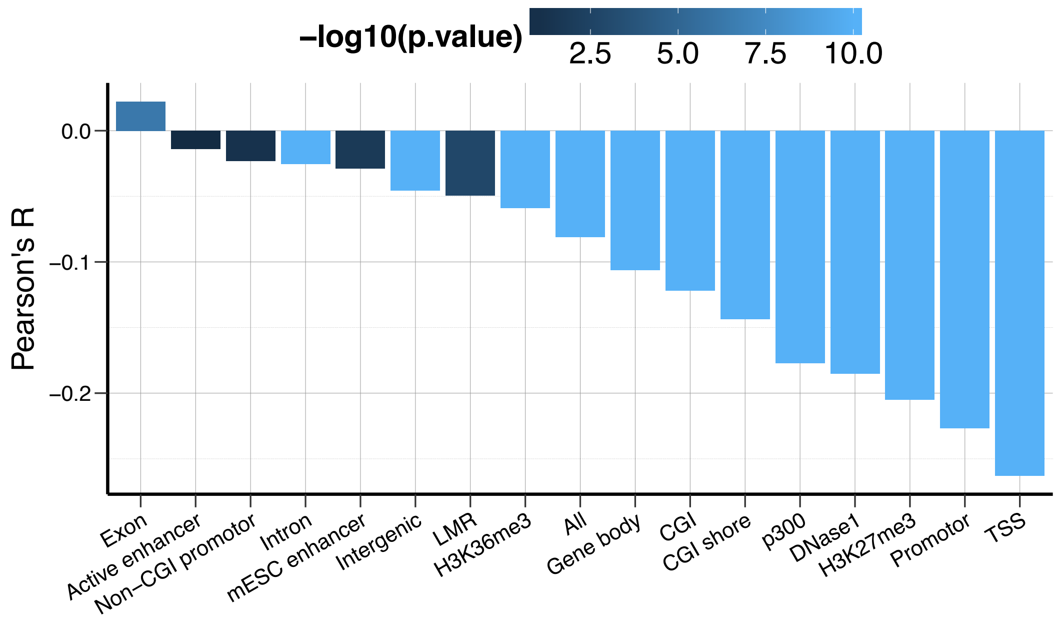


**Figure S13 | Correlation between estimated mutation effects and DNA sequence conservation.** Correlation between the estimated effect of single-nucleotide mutations and *PhastCons* conservation score for alternative contexts on test chromosomes. Estimated effects are significantly anti-correlated overall ('All', $P < 1.0{\times}10^{-15}$) in CpG dense regions.

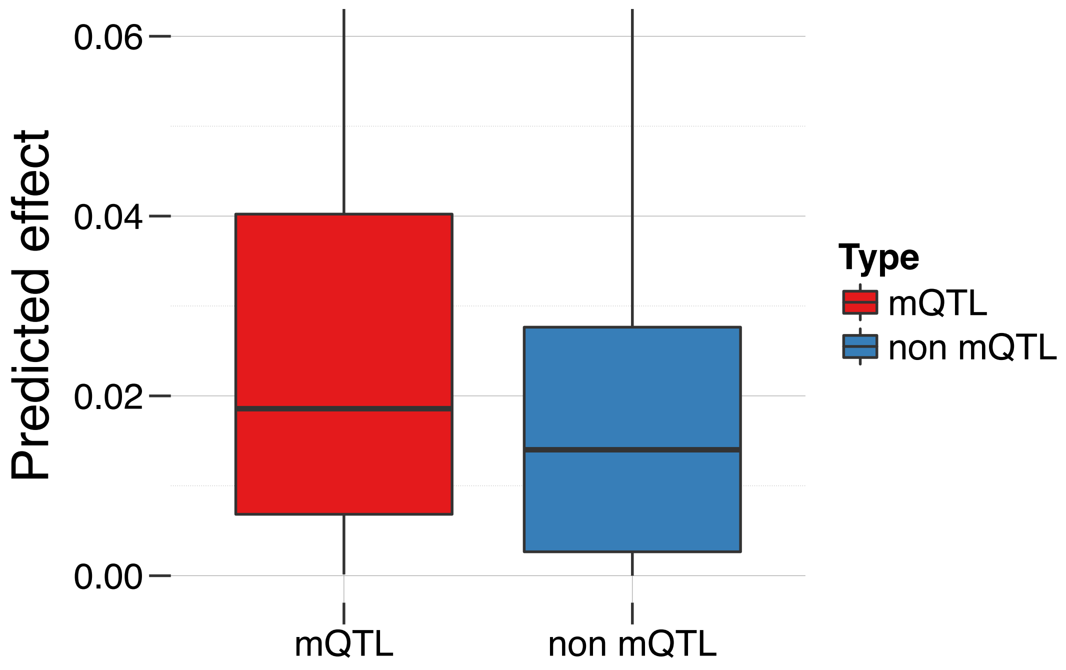


**Figure S14 | Mutation effects for methylation QTLs and non-methylation QTLs**. Distribution of the mutation effect for 2,379 methylation QTLs (mQTL) variants from Kaplow et al., compared to distance-matched random variants (non mQTL).

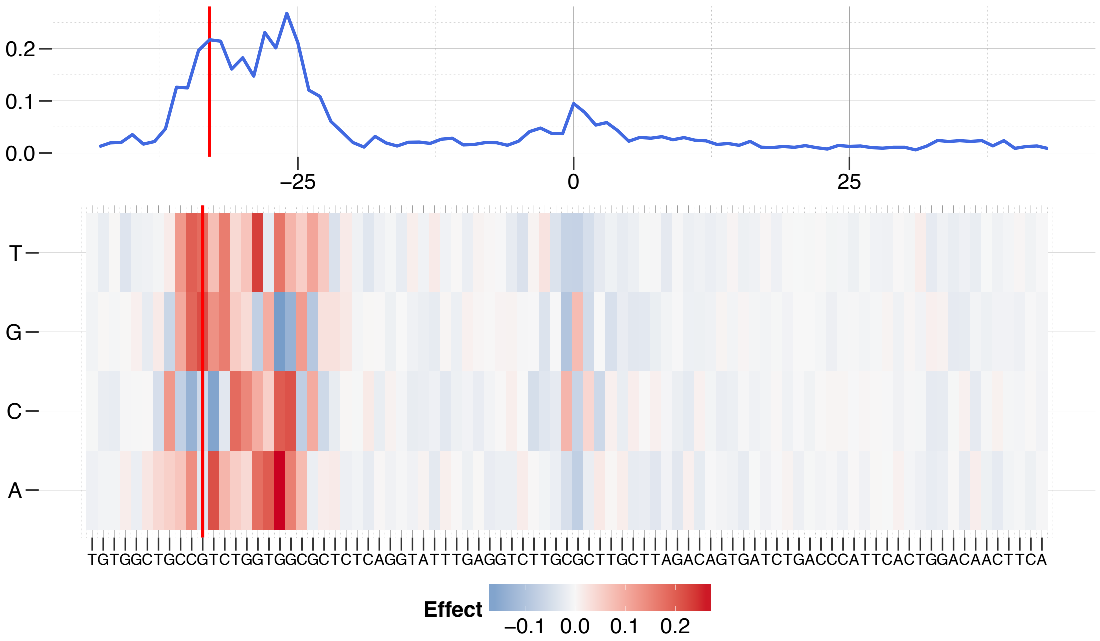


**Figure S15 | Visualization of mutation effects for an example CpG site and the corresponding mQTL.** Mutations effects in a window centered on an example CpG site (chromosome 1; position 159791497). The position of the corresponding mQTL from Kaplow et al (rs60205880; position 159791464) is indicated by the vertical red line. The heat map in the lower panels shows effect sizes for individual nucleotides, and line plot in the upper panel the maximum absolute effect across nucleotides.

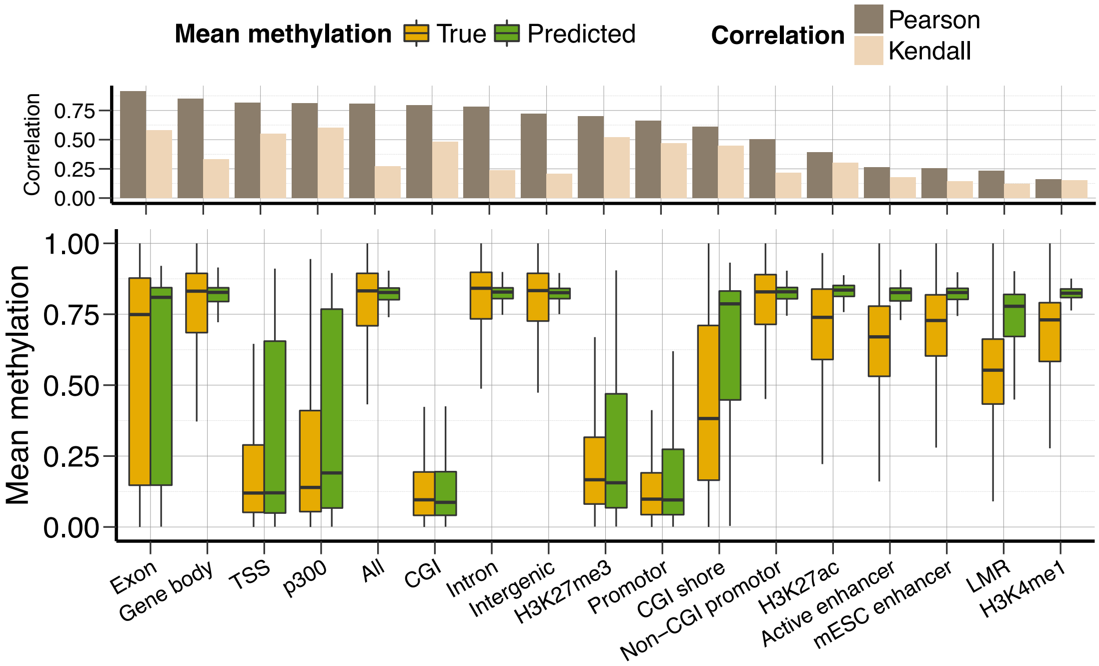


**Figure S16 | Prediction performance of mean methylation levels.** Histogram of predicted (green) and observed (orange) mean methylation levels in 3-kbp windows centered on indivual CpG sites on test chromosomes of serum mouse ESCs, along with Pearon's and Kendall's correlation coefficients on top.

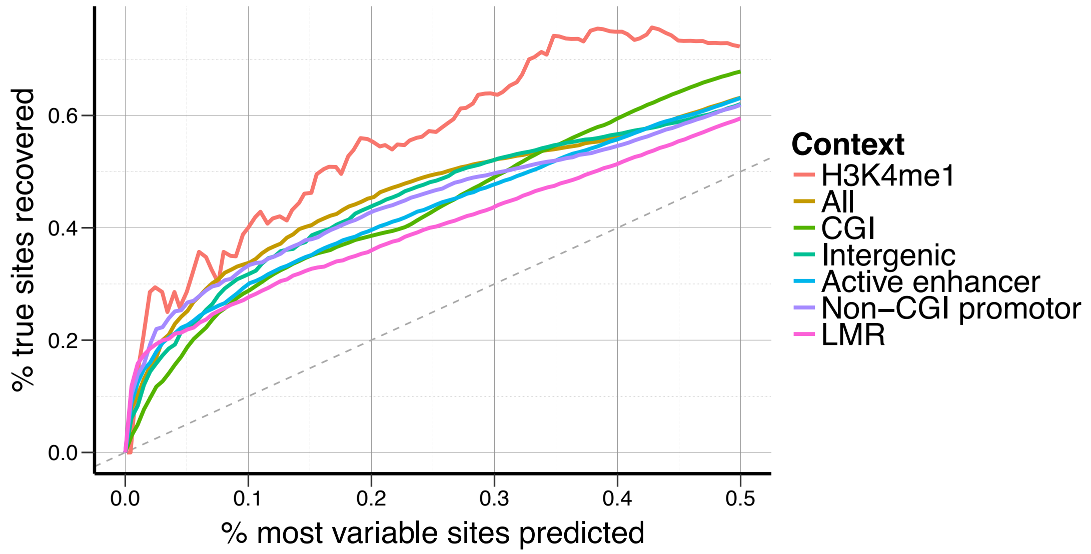


**Figure S17 | Sensitivity of discovering highly-variable CpG sites.** Sensitivity of discovering most variable sites for different thresholds and genomic contexts on test chromosomes. Individual CpG sites were ranked by the empirical variance estimated in 3-kbp windows centered on the CpG sites, or the variance predicted by DeepCpG, and the overlap computed for the fraction of most variables sites shown on the x-axis. The dashed line indicates the performance of a random ranking.

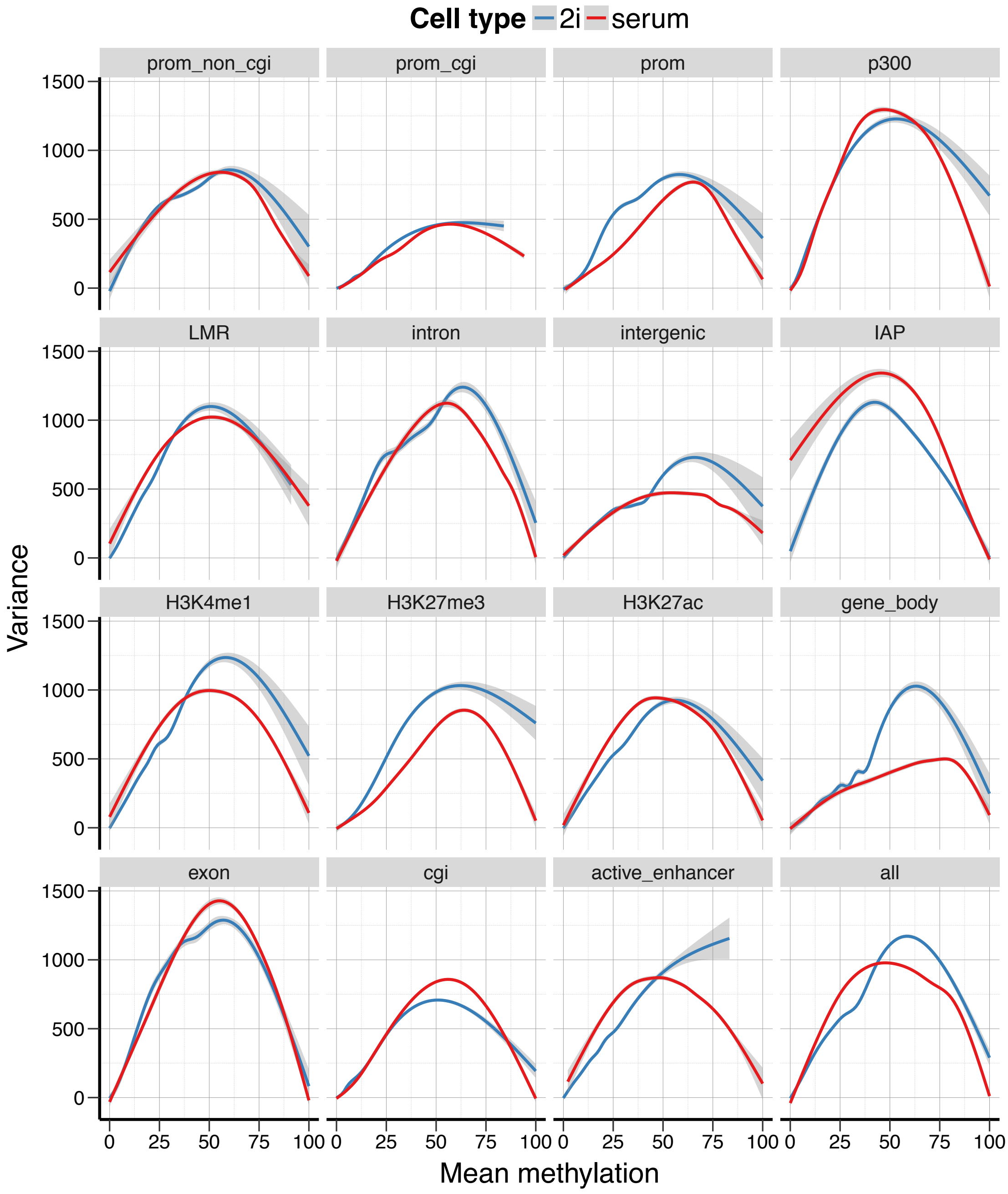


**Figure S18 | Dependency between mean methylation levels and cell-to-cell variance.** Smoothed regression fit between mean methylation levels (x-axis) verus cell-to-cell variance (y-axis), for 2i and serum cells in different genomic contexts. Cell-to-cell variance is linked to the mean methylation level, and highest for an intermediate methylation level of 50%.

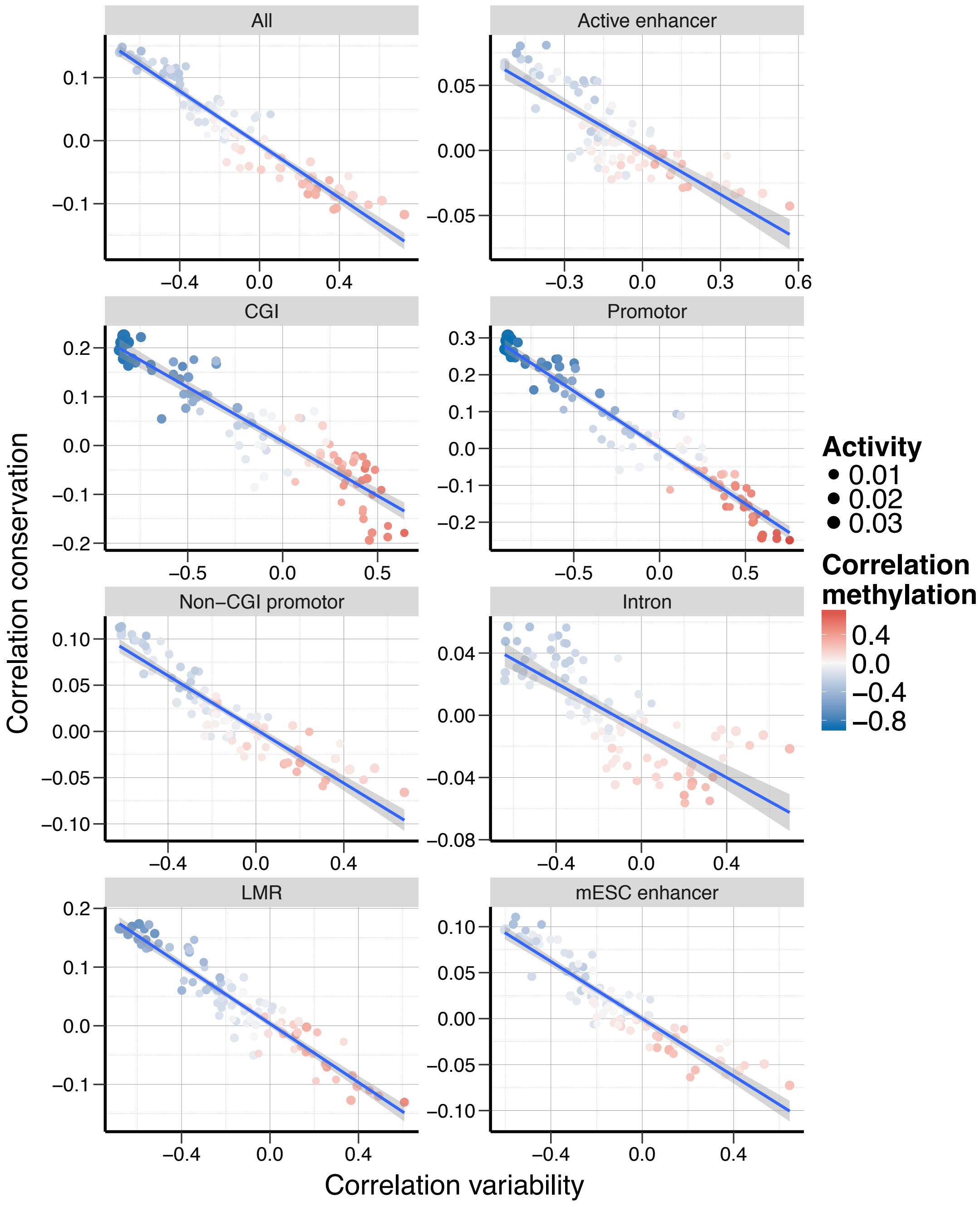


**Figure S19 | Linkage between motif correlation with sequence conservation and cell-to-cell variability.** Correlation of motif activities with cell-to-cell variability (x-axis), and DNA conservation (y-axis). Variability increasing motifs are most active in non-conserved regions. Individual dots correspond to motifs with their mean activity represented by the size, and influence on CpG methylation by colour.

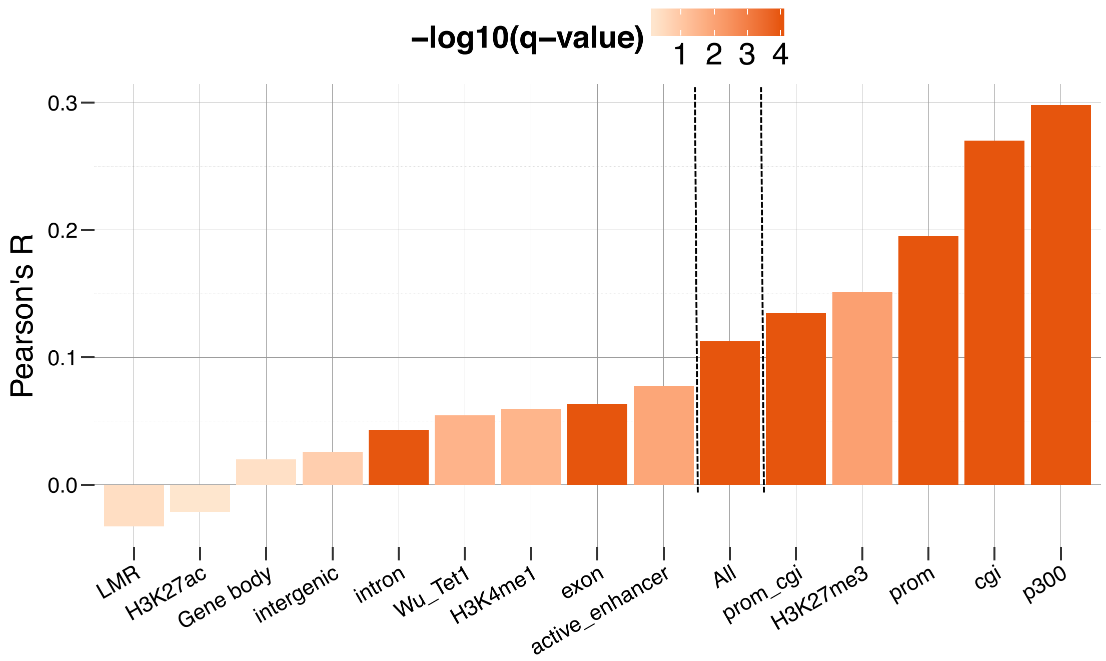


**Figure S20 | Functional assessment of predicted cell-to-cell variability**. Pearson correlation coefficient between methylome-transcriptome linkage as reported in Angermueller *et al.* (2016), and the predicted cell-to-cell variability for test chromosomes. Colour denotes statistical significance (q-value, Benjamini Hochberg adjusted). Significant correlations (FDR < 0.01) were obseved genome-wide ('All'), and in most indivdiual genomic contexts.

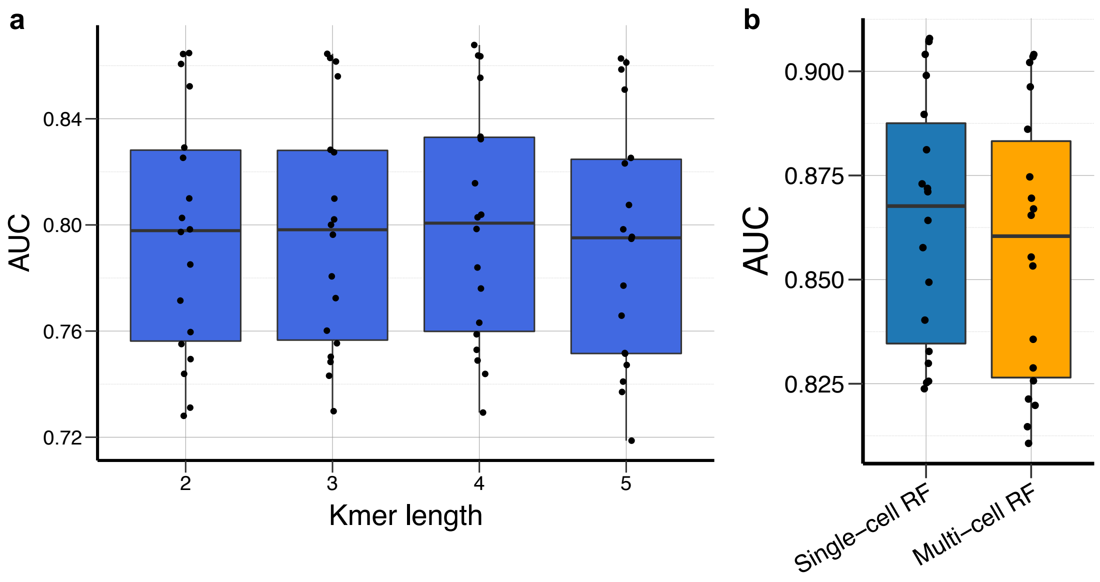


**Figure S21 | Prediction performance of random forest classifiers depending on the k-mer length and training strategy. (a)** Test AUC of random forest classifiers trained with k-mer frequencies of size one to five in 1001 bp DNA sequence windows centred on the target CpG site. **(b)** Test AUC of random forest classifiers trained on individual cells (Single-cell RF), vs. a single random forest classifier trained on all cells (Multi-cell RF).
