# Supplementary material for: DeepCpG: accurate prediction of single-cell DNA methylation states using deep learning
Source: Genome Biol. 2017 Apr 11;18:67. doi: 10.1186/s13059-017-1189-z (PMC5387360; doi:10.1186/s13059-017-1189-z)
Supplement: Supplementary file 3 — Sequence motifs. HTML files with sequence logos and summary statistics for all cell types. (ZIP 19482 kb) [file 13059_2017_1189_MOESM3_ESM.zip › 2i.html]

Filter table


# Filter table

#### *Christof Angermueller*

#### *2016-12-17*

Nr | Label | Logo | Influence | q-value | Related known motifs | GO annotations || 1 | 2: Klf8 |  |  | 0.000 | Klf8 (0.000) Klf4 (0.000) Klf5 (0.000) Klf12 (0.000) Klf2 (0.001) Klf7 (0.001) Sp5 (0.001) Klf7 (0.002) Klf1 (0.002) Klf16 (0.007) Sp8 (0.009) Zfp410 (0.012) Klf14 (0.016) Egr2 (0.016) Klf13 (0.019) Klf3 (0.047) Sp3 (0.047) Sp1 (0.062) Sp4 (0.069) Zfp281 (0.069) Sp4 (0.099) Zfp740 (0.110) Zfp148 (0.123) Wt1 (0.124) Smad3 (0.141) | BP: small GTPase mediated signal transduction BP: transcription CC: lysosome MF: hydrolase activity, acting on carbon-nitrogen (but not peptide) bonds MF: actin filament binding |
| 2 | 9: Klf4 |  |  | 0.000 | Klf4 (0.000) Klf8 (0.000) Klf5 (0.000) Sp5 (0.000) Klf12 (0.000) Klf2 (0.000) Klf16 (0.000) Klf7 (0.000) Sp8 (0.000) Klf14 (0.000) Klf7 (0.000) Klf1 (0.000) Sp4 (0.001) Sp3 (0.001) Sp1 (0.001) Klf13 (0.001) Egr2 (0.001) Zfp281 (0.003) Sp4 (0.003) Egr4 (0.003) Zfp148 (0.004) Wt1 (0.006) Klf3 (0.006) Smad3 (0.024) Egr3 (0.029) | BP: transcription BP: negative regulation of cell differentiation MF: transcription factor activity MF: sequence-specific DNA binding MF: transcription activator activity |
| 3 | 30: Zfp281 |  |  | 0.000 | Zfp281 (0.000) Zfp740 (0.000) Rreb1 (0.000) Wt1 (0.001) Maz (0.001) Sp1 (0.001) Zfp281 (0.001) Zfp740 (0.002) Sp3 (0.002) Zfp148 (0.003) Zfp219 (0.004) Klf15 (0.005) Zfp410 (0.005) Egr2 (0.007) Zbtb7a (0.008) Sp4 (0.008) E2f3 (0.015) Klf16 (0.015) Sp4 (0.018) Smad3 (0.018) Egr1 (0.019) Sp8 (0.021) Glis3 (0.024) Egr4 (0.024) Klf6 (0.031) Klf5 (0.031) | BP: transcription BP: inner ear morphogenesis CC: transcription factor complex MF: transcription activator activity MF: zinc ion binding |
| 4 | 33: Sp3 |  |  | 0.000 | Sp3 (0.000) Wt1 (0.000) Zfp281 (0.000) Klf16 (0.000) Sp1 (0.001) Egr4 (0.001) Zfp740 (0.001) Zbtb7a (0.001) Maz (0.001) Zfp219 (0.001) Sp5 (0.001) Klf5 (0.001) Sp8 (0.002) Klf15 (0.002) Klf6 (0.003) Klf14 (0.003) Egr2 (0.004) Zfp148 (0.005) E2f3 (0.006) Sp4 (0.006) Klf4 (0.006) Klf7 (0.006) E2f1 (0.007) Zfp281 (0.008) Sp4 (0.011) | BP: transcription BP: protein amino acid phosphorylation CC: transcription factor complex MF: zinc ion binding MF: ATP binding |
| 5 | 111: Ctcf |  |  | 0.000 | Ctcf (0.000) Ctcfl (0.000) Neurod1 (0.004) Zic4 (0.016) Sp4 (0.045) Scrt1 (0.068) Twist1 (0.068) Scrt2 (0.068) Atoh8 (0.068) Klf13 (0.072) Zic2 (0.077) Zic1 (0.083) Tcf3 (0.099) Zic5 (0.099) Zic3 (0.117) Myc (0.176) Atoh1 (0.225) Neurod2 (0.271) Max (0.271) Tcf4 (0.271) Tcf12 (0.271) Clock (0.271) Insm1 (0.271) Klf6 (0.288) Tal1 (0.291) Zic2 (0.291) Myog (0.291) | CC: extracellular region CC: plasma membrane CC: endoplasmic reticulum CC: photoreceptor inner segment MF: peptide receptor activity, G-protein coupled |
| 6 | 73: Klf2 |  |  | 0.001 | Klf2 (0.001) Klf5 (0.002) Sp4 (0.002) Klf4 (0.002) Klf8 (0.002) Klf12 (0.003) Klf1 (0.003) Klf7 (0.003) Sp5 (0.006) Klf7 (0.010) Klf16 (0.021) Klf14 (0.021) Klf3 (0.022) Sp3 (0.038) Wt1 (0.050) Klf13 (0.056) Sp8 (0.056) Egr2 (0.060) Sp4 (0.060) Egr4 (0.060) Klf15 (0.106) Ctcfl (0.138) Plagl1 (0.161) Bcl6b (0.163) Ascl2 (0.227) | BP: small GTPase mediated signal transduction BP: protein amino acid phosphorylation CC: lysosome MF: ATP binding MF: magnesium ion binding |
| 7 | 118: Sp3 |  |  | 0.001 | Sp3 (0.001) E2f1 (0.003) E2f3 (0.005) Wt1 (0.006) Sp1 (0.006) Klf6 (0.006) Zfp281 (0.006) Zbtb7a (0.007) Zfp740 (0.007) Zfp161 (0.007) Egr1 (0.014) Plagl1 (0.016) Zfp148 (0.017) Maz (0.020) Sp4 (0.020) Klf16 (0.020) Zfp219 (0.020) Zfp281 (0.020) E2f4 (0.025) Klf15 (0.035) Plagl1 (0.035) Egr1 (0.035) Egr4 (0.036) E2f6 (0.039) Rreb1 (0.043) Sp8 (0.043) E2F3 (0.043) | BP: transcription CC: transcription factor complex MF: ATP binding MF: zinc ion binding MF: transcription activator activity |
| 8 | 15: Zfp281 |  |  | 0.004 | Zfp281 (0.004) Zfp148 (0.004) Maz (0.004) Klf5 (0.005) Sp1 (0.005) Klf4 (0.006) Klf2 (0.007) Sp5 (0.007) Sp4 (0.007) Egr2 (0.007) Klf8 (0.007) Sp4 (0.007) Zfp281 (0.009) Sp8 (0.009) Klf14 (0.009) Rreb1 (0.009) Zfp740 (0.010) Klf16 (0.010) Zfp740 (0.010) Sp3 (0.011) Klf7 (0.011) Klf12 (0.011) Klf7 (0.011) Gli2 (0.013) Klf1 (0.013) | BP: transcription BP: anterior/posterior pattern formation BP: negative regulation of transcription from RNA polymerase II promoter BP: negative regulation of cell proliferation MF: transcription activator activity |
| 9 | 51: Rreb1 |  |  | 0.005 | Rreb1 (0.005) Zfp281 (0.013) Sp1 (0.013) Klf16 (0.050) Zfp740 (0.050) Zbtb7a (0.050) Sp8 (0.050) Sp3 (0.050) Zic3 (0.050) Zic2 (0.050) Zfp219 (0.061) Klf6 (0.082) Wt1 (0.082) Zfp740 (0.082) Sp4 (0.082) Eomes (0.082) Zfp410 (0.082) Klf7 (0.082) Maz (0.084) Runx3 (0.089) Zfp148 (0.092) Klf15 (0.096) Klf14 (0.096) Zic5 (0.096) Glis2 (0.096) | BP: transcription BP: negative regulation of transcription from RNA polymerase II promoter BP: inner ear morphogenesis CC: transcription factor complex MF: transcription activator activity |
| 10 | 1: Zfp161 |  |  | 0.007 | Zfp161 (0.007) E2f1 (0.049) Plagl1 (0.072) Egr4 (0.092) Egr1 (0.138) Wt1 (0.175) Sp3 (0.175) E2f3 (0.175) Sp2 (0.185) Gata6 (0.218) Egr1 (0.218) Egr3 (0.304) Zbtb7a (0.304) Tcfap2d (0.304) Zfx (0.304) Zfp161 (0.304) Nr2f2 (0.361) Ctcfl (0.445) Tcfap2c (0.445) Hes1 (0.445) E2F3 (0.456) Hes7 (0.464) Mbd2 (0.464) Myf6 (0.464) Rarb (0.464) Zic1 (0.464) | BP: transcription BP: negative regulation of transcription from RNA polymerase II promoter CC: transcription factor complex MF: ATP binding MF: zinc ion binding |
| 11 | 64: Ehf |  |  | 0.007 | Ehf (0.007) Elf5 (0.023) Elf3 (0.045) Elf3 (0.045) Elf1 (0.072) Spdef (0.107) Elf4 (0.107) Ets2 (0.137) Ehf (0.143) Elf2 (0.188) Etv6 (0.222) Elk4 (0.339) Etv5 (0.416) Elk1 (0.439) Irx2 (0.487) Etv3 (0.487) Gm4881 (0.496) Fli1 (0.507) Elk3 (0.560) Mybl1 (0.658) Erf (0.685) Osr1 (0.737) Gabpa (0.737) Stat3 (0.737) Etv2 (0.737) Gabpa (0.737) | BP: cell division BP: mitosis CC: spliceosomal complex MF: ATP binding MF: structural constituent of ribosome |
| 12 | 88: Rara |  |  | 0.011 | Rara (0.011) Nr2f2 (0.011) Prkrir (0.091) Tcf7l2 (0.091) Nr4a2 (0.130) Lef1 (0.139) Pparg (0.139) Nr2c1 (0.139) Nr4a3 (0.139) Rarb (0.162) Nr2f6 (0.206) Tcf7 (0.229) Rarg (0.229) Nr1h2 (0.229) Ctcf (0.229) Rara (0.229) Tcf3 (0.229) Rfx3 (0.229) Hnf4g (0.229) Ppara (0.229) Pdx1 (0.229) Nr4a1 (0.231) Esrra (0.239) Esrrb (0.353) Rxra (0.379) | BP: sensory perception of smell BP: G-protein coupled receptor protein signaling pathway BP: defense response to bacterium CC: integral to membrane MF: olfactory receptor activity |
| 13 | 114: Ctcf |  |  | 0.016 | Ctcf (0.016) Ctcfl (0.395) Zic2 (0.395) Plag1 (0.395) Zic2 (0.395) Zic3 (0.395) Zic3 (0.395) Zic1 (0.498) Zfp281 (0.576) Zic1 (0.602) Zbtb7b (0.886) Zfp202 (0.886) Zic5 (0.886) Hsf1 (0.886) Tcfap2c (0.886) Tcfap2a (0.887) Ebf1 (0.887) Zbtb7b (0.898) Glis1 (0.898) | BP: response to external stimulus BP: immune response CC: plasma membrane CC: extracellular space CC: proteinaceous extracellular matrix |
| 14 | 108: Srf |  |  | 0.018 | Srf (0.018) Elf3 (0.151) Zfp105 (0.887) Mtf1 (0.887) Tcf3 (0.887) Foxa2 (0.887) | BP: sensory perception of smell BP: G-protein coupled receptor protein signaling pathway CC: spliceosomal complex MF: olfactory receptor activity MF: RNA binding |
| 15 | 57: Glis2 |  |  | 0.020 | Glis2 (0.020) Sry (0.212) Arid3b (0.212) Dbx1 (0.212) Sox14 (0.212) Pit1 (0.212) Sox21 (0.227) Phf21a (0.319) Hoxa9 (0.319) Foxd4 (0.337) Arid5a (0.337) Hoxa10 (0.459) Mtf1 (0.471) Tbp (0.536) Pou4f3 (0.646) Hoxd10 (0.646) Pou3f4 (0.646) Arid5a (0.646) Pou1f1 (0.659) Hoxa4 (0.659) Sox30 (0.659) Arid3a (0.659) Tlx2 (0.659) Six6 (0.685) Hmg20b (0.685) | BP: G-protein coupled receptor protein signaling pathway BP: sensory perception of smell BP: signal transduction BP: cell communication MF: olfactory receptor activity |
| 16 | 67: Sp4 |  |  | 0.020 | Sp4 (0.020) Sp3 (0.020) Sp4 (0.020) Klf6 (0.020) Maz (0.031) Egr1 (0.033) Zfp219 (0.035) Pitx1 (0.035) Sp1 (0.045) Obox2 (0.045) Klf14 (0.046) Wt1 (0.066) Obox5 (0.066) Obox3 (0.066) Bcl6 (0.068) Sp5 (0.073) Zfp281 (0.073) Zfp148 (0.075) E2f1 (0.077) Zfp281 (0.079) Sp8 (0.089) Smad3 (0.098) Obox1 (0.110) Klf16 (0.113) Zfp263 (0.127) | BP: transcription BP: negative regulation of transcription from RNA polymerase II promoter BP: protein amino acid phosphorylation BP: embryonic limb morphogenesis MF: transcription activator activity |
| 17 | 43: Lcor |  |  | 0.024 | Lcor (0.024) Mef2c (0.113) Mef2a (0.113) | BP: sensory perception of smell BP: G-protein coupled receptor protein signaling pathway BP: signal transduction BP: cell communication MF: olfactory receptor activity |
| 18 | 72: Pou6f1 |  |  | 0.027 | Pou6f1 (0.027) Pou6f2 (0.476) Vsx2 (0.476) Pou2f2 (0.621) Pou2f3 (0.621) Hoxa3 (0.621) Pou3f2 (0.621) Rhox6 (0.744) Pou6f1 (0.744) Lhx9 (0.744) Pou2f3 (0.744) Lhx2 (0.744) Pdx1 (0.744) Hoxc9 (0.744) Lbx2 (0.744) Pou6f1 (0.744) Pou3f4 (0.744) Pou4f2 (0.744) Pou2f2 (0.744) Dobox4 (0.744) Lhx4 (0.744) Hoxd3 (0.744) Gata3 (0.744) Hoxb3 (0.744) Lhx4 (0.744) Evx1 (0.744) Mnx1 (0.744) Zbtb12 (0.744) Atf2 (0.744) Lhx3 (0.744) Hoxa2 (0.744) Emx2 (0.744) Prrxl1 (0.744) Lhx2 (0.744) Duxbl (0.744) Pou3f1 (0.744) Hoxb3 (0.744) Mecom (0.744) Hoxc13 (0.744) Vax2 (0.744) Arx (0.744) Prop1 (0.744) Prrx2 (0.744) Prrx1 (0.744) Meox1 (0.744) Gsx2 (0.744) Hoxb5 (0.744) Rax (0.744) Nkx1-2 (0.744) Mixl1 (0.744) Pou4f3 (0.744) Pou2f1 (0.744) Vsx1 (0.744) Lhx6 (0.744) Evx2 (0.744) Gata6 (0.744) Ipf1 (0.744) Hoxb5 (0.744) Lhx1 (0.744) Lhx5 (0.744) Pax6 (0.744) | BP: sensory perception of smell BP: G-protein coupled receptor protein signaling pathway CC: integral to membrane CC: external side of plasma membrane MF: olfactory receptor activity |
| 19 | 21 |  |  | 0.054 | Tbp (0.054) Zfp637 (0.208) Zfp128 (0.762) Pou3f3 (0.762) Cdc5l (0.830) | BP: G-protein coupled receptor protein signaling pathway BP: sensory perception of smell BP: signal transduction CC: integral to membrane MF: olfactory receptor activity |
| 20 | 83 |  |  | 0.054 | Snai2 (0.054) Figla (0.105) Atoh8 (0.122) Creb3l2 (0.122) Tcfe2a (0.122) Tcf15 (0.141) Hey1 (0.141) Zbtb49 (0.141) Insm1 (0.141) Max (0.152) Mycn (0.175) Clock (0.180) Max (0.180) Tcf4 (0.181) Atoh1 (0.181) Max (0.183) Twist1 (0.183) Mitf (0.183) Myb (0.183) Tcfe3 (0.183) Scrt2 (0.194) Klf15 (0.206) Myf6 (0.206) Mtf1 (0.206) Usf2 (0.206) | BP: heterophilic cell-cell adhesion CC: endoplasmic reticulum MF: transcription factor activity MF: protein binding MF: sequence-specific DNA binding |
| 21 | 7 |  |  | 0.066 | Tbx19 (0.066) Hif3a (0.751) Sox17 (0.751) Mesp2 (0.751) | BP: sensory perception of smell BP: G-protein coupled receptor protein signaling pathway BP: signal transduction CC: integral to membrane MF: olfactory receptor activity |
| 22 | 50 |  |  | 0.066 | Klf1 (0.066) Klf2 (0.066) Eomes (0.188) Ctcfl (0.188) Ctcf (0.193) Klf4 (0.224) Klf3 (0.224) Klf5 (0.287) Sp4 (0.301) Klf8 (0.314) Plagl1 (0.423) Klf12 (0.423) Klf7 (0.423) Klf15 (0.423) Yy1 (0.423) Zscan20 (0.423) Plagl1 (0.423) Klf7 (0.423) Plagl1 (0.492) Sp5 (0.558) Mbtps2 (0.558) Zfx (0.677) Klf14 (0.677) Tcf15 (0.703) Six2 (0.703) Fosl2 (0.703) Zbtb7a (0.703) Zfp281 (0.703) Zbtb6 (0.703) 9430076C15Rik (0.703) Esr1 (0.703) | BP: transcription BP: small GTPase mediated signal transduction MF: transcription factor activity MF: sequence-specific DNA binding MF: GTP binding |
| 23 | 101 |  |  | 0.078 | Rest (0.078) Esrrb (0.320) Nr1h2 (0.320) Nr6a1 (0.320) Esr2 (0.320) Esrrg (0.320) Nr4a3 (0.320) Nr5a1 (0.320) Nr5a2 (0.345) Ppard (0.421) Trp53 (0.421) Pparg (0.421) Esrra (0.423) Ppara (0.596) Nr3c2 (0.596) Esrra (0.858) | BP: G-protein coupled receptor protein signaling pathway BP: sensory perception of smell CC: integral to membrane MF: serine-type endopeptidase activity MF: olfactory receptor activity |
| 24 | 49 |  |  | 0.081 | Klf7 (0.081) | BP: mRNA processing BP: RNA splicing MF: transcription factor activity MF: ATP binding MF: zinc ion binding |
| 25 | 79 |  |  | 0.088 | Nr2c2 (0.088) Elf1 (0.088) Gabpa (0.088) Ctcf (0.088) Ets2 (0.153) Erg (0.209) Ets1 (0.235) Elf5 (0.334) Ctcfl (0.345) Ehf (0.369) Ehf (0.412) Stat4 (0.420) Erf (0.474) Elf4 (0.474) Scrt2 (0.474) Nkx1-1 (0.477) Elf2 (0.539) Scrt1 (0.539) Twist1 (0.539) Elf3 (0.539) Spdef (0.587) Evx1 (0.587) Spdef (0.644) Emx2 (0.661) Fev (0.685) | BP: transport BP: negative regulation of cellular process BP: regulation of transcription from RNA polymerase II promoter CC: endoplasmic reticulum MF: zinc ion binding |
| 26 | 104 |  |  | 0.089 | Pou3f2 (0.089) Phf21a (0.089) Alx3 (0.114) Otx1 (0.114) Uncx4.1 (0.114) Nkx6-3 (0.114) Nkx6-1 (0.114) Hoxb4 (0.114) Pou1f1 (0.121) Hoxd1 (0.127) Arid3a (0.127) Obox5 (0.127) Lhx3 (0.127) Lhx2 (0.127) Cart1 (0.127) Sebox (0.127) Lhx5 (0.127) Msx3 (0.127) Rhox6 (0.127) Hoxd8 (0.127) Arid3b (0.127) Sox11 (0.127) Isl2 (0.127) Lhx1 (0.127) Pou3f4 (0.127) Lhx6 (0.127) Evx1 (0.127) Phox2b (0.127) Lmx1b (0.127) Dbx1 (0.127) Pax4 (0.127) Pou2f1 (0.127) Dbx1 (0.127) Lhx3 (0.127) Hoxc5 (0.127) Pbx2 (0.127) Otp (0.127) Tlx2 (0.127) Msx1 (0.127) Hmbox1 (0.127) Hoxc4 (0.127) Hoxc6 (0.127) En1 (0.127) Tlx2 (0.127) | BP: sensory perception of smell BP: G-protein coupled receptor protein signaling pathway BP: signal transduction BP: cell communication MF: olfactory receptor activity |
| 27 | 121 |  |  | 0.089 | Esrra (0.089) Nr2f2 (0.089) Rara (0.629) Rxrg (0.629) Rxra (0.629) Rxrb (0.629) E4f1 (0.629) Nr2f2 (0.629) Rarb (0.629) Atf1 (0.629) Pknox1 (0.629) Nr2c1 (0.629) Egr4 (0.629) Creb1 (0.629) Xbp1 (0.629) Nr2f1 (0.629) Zic4 (0.629) Nr2f6 (0.629) Hnf4a (0.629) Glis3 (0.629) Rfx3 (0.629) Eomes (0.629) Thra (0.629) Ppard (0.629) Rara (0.629) Ctcfl (0.629) Meis3 (0.629) Nr4a2 (0.629) Six4 (0.629) Jundm2 (0.629) Atf6 (0.629) Meis2 (0.629) Pknox1 (0.629) Clock (0.629) Klf7 (0.629) Egr1 (0.629) Insm1 (0.629) Zic5 (0.629) Hnf4a (0.629) Gmeb1 (0.629) Fosl2 (0.629) Hes7 (0.629) Pknox2 (0.629) Creb3l2 (0.629) Bhlhb2 (0.629) Ppara (0.629) Tcfap2a (0.629) | BP: mRNA processing BP: RNA splicing MF: ATP binding MF: RNA binding MF: transcription factor activity |
| 28 | 0 |  |  | 0.117 | Plagl1 (0.117) Dobox4 (0.117) Plagl1 (0.181) Glis2 (0.186) Rarb (0.186) Six1 (0.187) Glis2 (0.187) Dobox4 (0.188) Zic5 (0.301) Plagl1 (0.354) Six1 (0.354) Six6 (0.354) Hivep1 (0.354) Six3 (0.394) Mzf1 (0.394) Six4 (0.394) Rara (0.394) Glis3 (0.394) Nr4a1 (0.394) Rarg (0.394) Six6 (0.394) Zfp281 (0.394) Six6 (0.394) Nr2c1 (0.394) Zfp740 (0.394) | BP: regulation of production of small RNA involved in gene silencing by RNA BP: regulation of system process CC: proteinaceous extracellular matrix CC: extracellular space MF: calcium ion binding |
| 29 | 53 |  |  | 0.145 | Pgr (0.145) ENSMUSG00000044690 (0.145) Fev (0.194) | BP: regulation of production of small RNA involved in gene silencing by RNA BP: innate immune response CC: extracellular space MF: peptide receptor activity, G-protein coupled MF: cytokine activity |
| 30 | 127 |  |  | 0.157 | Obox6 (0.157) Spdef (0.350) Dmbx1 (0.350) Obox1 (0.350) Crx (0.436) Obox6 (0.647) Spib (0.647) Obox2 (0.647) Gsc2 (0.647) Spic (0.647) Nr2c2 (0.647) Obox1 (0.647) Dmbx1 (0.647) Rhox11 (0.647) Zfp354c (0.772) Sfpi1 (0.851) Elk3 (0.851) Elk4 (0.866) Elk1 (0.866) Etv3 (0.866) Ybx1 (0.866) Pitx3 (0.866) Fli1 (0.866) Myc (0.866) Etv1 (0.866) Gm4881 (0.866) Zbtb3 (0.866) Tcfcp2 (0.866) | BP: cell division MF: transcription factor activity MF: sequence-specific DNA binding MF: ATP binding MF: zinc ion binding |
| 31 | 13 |  |  | 0.162 | Nr5a1 (0.162) Nr1h2 (0.189) Nr6a1 (0.189) Esrrg (0.189) Esrra (0.325) Ppara (0.364) Nr4a2 (0.364) Nr4a1 (0.382) Esrra (0.500) Myt1l (0.537) Nr5a2 (0.547) Rxra (0.568) Nr4a3 (0.601) Pparg (0.601) Titf1 (0.624) Rara (0.789) Tcf7 (0.789) | BP: sensory perception of smell BP: G-protein coupled receptor protein signaling pathway BP: signal transduction BP: response to pheromone MF: olfactory receptor activity |
| 32 | 52 |  |  | 0.163 | Klf3 (0.163) Klf1 (0.167) Twist2 (0.167) Klf4 (0.167) Klf8 (0.167) Klf2 (0.167) Bhlhe22 (0.167) Ets1 (0.167) Erg (0.167) Klf12 (0.167) Klf7 (0.167) Neurod2 (0.167) Zfp238 (0.167) Gabpa (0.177) Scrt1 (0.213) Bhlhe23 (0.213) Scrt2 (0.295) Neurod1 (0.295) Neurog2 (0.308) Neurog3 (0.308) Olig1 (0.339) Olig2 (0.363) Creb3l2 (0.363) Mycn (0.363) Clock (0.363) | BP: sensory perception of smell BP: G-protein coupled receptor protein signaling pathway CC: integral to membrane MF: olfactory receptor activity MF: sugar binding |
| 33 | 54 |  |  | 0.238 | Nfic (0.238) Hic2 (0.622) Hic1 (0.622) Nfix (0.622) | BP: sensory perception of smell BP: G-protein coupled receptor protein signaling pathway BP: signal transduction CC: integral to membrane MF: olfactory receptor activity |
| 34 | 61 |  |  | 0.239 | Prdm4 (0.239) | BP: immune response BP: defense response to bacterium CC: extracellular space MF: iron ion binding MF: cytokine activity |
| 35 | 89 |  |  | 0.269 | Zfp740 (0.269) Glis1 (0.269) Zbtb7b (0.269) Tcfap2c (0.269) E2f5 (0.269) Zfp740 (0.269) Insm1 (0.269) Zfp524 (0.336) Irf6 (0.351) Zfp410 (0.381) Sox13 (0.703) Irf5 (0.703) Bcl6b (0.703) Egr1 (0.703) | BP: transcription BP: anterior/posterior pattern formation MF: transcription factor activity MF: sequence-specific DNA binding MF: RNA binding |
| 36 | 4 |  |  | 0.301 | Bcl6 (0.301) Nr1h2 (0.301) Esr2 (0.301) Nr1i3 (0.301) Sox3 (0.301) Ppard (0.301) Lef1 (0.301) Smad1 (0.338) Sox6 (0.338) Maz (0.338) Atf5 (0.338) Lef1 (0.338) Insm1 (0.338) Tcf7l2 (0.349) Tcf7l2 (0.388) Ppara (0.388) Rxra (0.388) Nr1i2 (0.392) Sox4 (0.392) Nr3c1 (0.392) Zfp524 (0.469) Tcf3 (0.542) Sox11 (0.542) Tcf7 (0.542) Sox9 (0.548) | CC: proteinaceous extracellular matrix CC: integral to plasma membrane MF: calcium ion binding MF: sequence-specific DNA binding MF: transcription factor activity |
| 37 | 35 |  |  | 0.314 | Egr4 (0.314) Erf (0.360) Elf1 (0.839) Gabpa (0.839) Gabpa (0.839) ENSMUSG00000044690 (0.839) Zic3 (0.839) Zfp740 (0.839) Fev (0.839) Ebf1 (0.839) Dobox5 (0.839) Etv5 (0.839) Ehf (0.839) Smad1 (0.839) Zic1 (0.839) Zic3 (0.839) Nr2c2 (0.839) Elk1 (0.839) Gm5454 (0.839) Zbtb3 (0.839) Etv4 (0.839) Irf6 (0.839) Zic2 (0.839) Gfi1b (0.839) Elk3 (0.839) Zic4 (0.839) Egr1 (0.839) Etv1 (0.839) | BP: transcription BP: protein amino acid phosphorylation BP: positive regulation of transcription from RNA polymerase II promoter MF: transcription factor activity MF: ATP binding |
| 38 | 84 |  |  | 0.356 | Zfp740 (0.356) Nr2c2 (0.394) Zbtb1 (0.663) Stat1 (0.785) Ehf (0.811) | BP: transcription BP: positive regulation of transcription from RNA polymerase II promoter MF: zinc ion binding MF: ATP binding MF: transcription activator activity |
| 39 | 42 |  |  | 0.368 | Arid5b (0.368) | BP: sensory perception of smell BP: G-protein coupled receptor protein signaling pathway BP: signal transduction BP: cell communication MF: olfactory receptor activity |
| 40 | 39 |  |  | 0.371 | Tcf7 (0.371) Tcf3 (0.371) Tcf7l2 (0.371) Nr1h2 (0.405) Nr2f2 (0.405) Ppard (0.632) Prkrir (0.861) Rara (0.861) Foxp4 (0.861) Lhx4 (0.861) Pparg (0.875) | BP: sensory perception of smell BP: G-protein coupled receptor protein signaling pathway CC: integral to membrane MF: olfactory receptor activity MF: serine-type endopeptidase inhibitor activity |
| 41 | 31 |  |  | 0.405 | Nfix (0.405) Nfic (0.405) Nfib (0.821) | BP: response to external stimulus BP: immune response CC: integral to membrane CC: proteinaceous extracellular matrix MF: serine-type endopeptidase activity |
| 42 | 91 |  |  | 0.433 | Bapx1 (0.433) | BP: sensory perception of smell BP: G-protein coupled receptor protein signaling pathway BP: nuclear mRNA splicing, via spliceosome MF: olfactory receptor activity MF: RNA binding |
| 43 | 85 |  |  | 0.434 | Irx5 (0.434) Irx3 (0.434) Irx2 (0.434) Irx4 (0.434) Myf6 (0.434) Bhlhe40 (0.434) Irx6 (0.695) Ar (0.695) Irx3 (0.733) Irx4 (0.754) Irx6 (0.754) | BP: sensory perception of smell BP: G-protein coupled receptor protein signaling pathway BP: cell communication BP: response to pheromone MF: olfactory receptor activity |
| 44 | 126 |  |  | 0.457 | Sox5 (0.457) Pou6f1 (0.545) Cdc5l (0.545) Nfil3 (0.545) Hmg20b (0.561) Pou3f3 (0.810) | BP: G-protein coupled receptor protein signaling pathway BP: sensory perception of smell BP: signal transduction CC: integral to membrane MF: olfactory receptor activity |
| 45 | 29 |  |  | 0.495 | Msx3 (0.495) Hoxb13 (0.495) Onecut1 (0.495) Hoxb9 (0.495) Barhl2 (0.495) Hoxa3 (0.495) Onecut3 (0.528) Barhl1 (0.528) Onecut2 (0.549) Isl2 (0.838) Hoxd13 (0.838) Hoxd10 (0.838) Hoxd13 (0.838) Hoxb6 (0.838) Cdx2 (0.838) Hoxd9 (0.838) | BP: sensory perception of smell BP: G-protein coupled receptor protein signaling pathway BP: signal transduction CC: integral to membrane MF: olfactory receptor activity |
| 46 | 10 |  |  | 0.510 | Foxa3 (0.510) Pou2f3 (0.510) Foxa2 (0.510) Pou2f2 (0.510) Foxf2 (0.510) Sox2 (0.537) Pou5f1 (0.579) Foxa1 (0.579) Rhox11 (0.824) Foxs1 (0.842) | BP: sensory perception of smell BP: G-protein coupled receptor protein signaling pathway BP: signal transduction BP: cell communication MF: olfactory receptor activity |
| 47 | 105 |  |  | 0.514 | Klf7 (0.514) Klf8 (0.514) Klf2 (0.514) Klf12 (0.514) Klf4 (0.514) Klf1 (0.537) | BP: ribosome biogenesis CC: mitochondrion CC: ribonucleoprotein complex MF: RNA binding MF: protein binding |
| 48 | 81 |  |  | 0.551 | Nr1h4 (0.551) Esrra (0.551) Sox15 (0.606) Esrra (0.606) Trp53 (0.631) Hoxa3 (0.631) Bbx (0.631) Esrrg (0.631) Nr5a1 (0.631) Hbp1 (0.631) Sox9 (0.631) Nr2f2 (0.631) Bbx (0.631) Sox6 (0.631) Mecp2 (0.631) Irx5 (0.729) Pax8 (0.761) Rara (0.783) Esrrb (0.783) Sox30 (0.783) Ppara (0.783) Prkrir (0.783) Pdx1 (0.783) Irx4 (0.783) Myb (0.790) | BP: sensory perception of smell BP: G-protein coupled receptor protein signaling pathway BP: signal transduction BP: cell communication MF: olfactory receptor activity |
| 49 | 75 |  |  | 0.585 | Pou2f2 (0.585) Pou2f3 (0.585) Foxo1 (0.585) Nkx2-6 (0.585) Mixl1 (0.585) Nkx1-2 (0.608) Rax (0.608) Bapx1 (0.608) Vsx1 (0.608) Shox2 (0.608) Alx4 (0.884) Hmx3 (0.884) Alx1 (0.884) Arx (0.884) Lhx2 (0.884) Lhx9 (0.884) Uncx (0.884) Lbx2 (0.884) Lhx8 (0.884) Isx (0.884) | BP: sensory perception of smell BP: G-protein coupled receptor protein signaling pathway BP: signal transduction CC: integral to membrane MF: olfactory receptor activity |
| 50 | 34 |  |  | 0.586 | Mbd2 (0.586) | BP: mRNA processing BP: negative regulation of transcription from RNA polymerase II promoter CC: transcription factor complex MF: ATP binding MF: zinc ion binding |
| 51 | 74 |  |  | 0.679 | Arid3a (0.679) Irx6 (0.679) | BP: sensory perception of smell BP: G-protein coupled receptor protein signaling pathway BP: signal transduction BP: cell communication MF: olfactory receptor activity |
| 52 | 76 |  |  | 0.711 | Hic2 (0.711) Mbtps2 (0.711) Yy1 (0.711) Plagl1 (0.711) Smarcc1 (0.711) Eomes (0.711) | BP: transcription BP: protein amino acid phosphorylation MF: transcription factor activity MF: ATP binding MF: zinc ion binding |
| 53 | 47 |  |  | 0.732 | Thra (0.732) | BP: sensory perception of smell BP: G-protein coupled receptor protein signaling pathway CC: integral to membrane CC: extracellular region MF: olfactory receptor activity |
| 54 | 98 |  |  | 0.738 | Tcfap2c (0.738) Tcfap2a (0.738) Erg (0.738) Tcfap2e (0.738) Tcfap2a (0.738) Tcfap2e (0.747) Nr2c2 (0.747) Zbtb3 (0.747) ENSMUSG00000044690 (0.747) Tcfap2b (0.747) Elk3 (0.747) Bcl11a (0.747) Zbtb3 (0.747) Sfpi1 (0.754) Etv5 (0.754) Nr4a3 (0.767) Scrt1 (0.767) Etv1 (0.767) Tcfap2c (0.767) Spic (0.767) Rxra (0.767) Tcf3 (0.767) Etv6 (0.767) Tcfap2c (0.767) Zic2 (0.767) Etv4 (0.767) Spib (0.767) Tcfap2d (0.767) Runx1 (0.767) Erf (0.767) Ets1 (0.767) Elk1 (0.767) Tcfap2b (0.767) Zbtb3 (0.767) | BP: angiogenesis BP: protein amino acid phosphorylation MF: transcription factor activity MF: sequence-specific DNA binding MF: ATP binding |
| 55 | 82 |  |  | 0.740 | Tcfap2c (0.740) Etv4 (0.740) Gm5454 (0.740) Etv1 (0.740) Rara (0.740) E2f8 (0.740) Elk1 (0.740) Etv5 (0.740) Fli1 (0.740) Elk4 (0.740) Gm4881 (0.740) Tcf1 (0.740) Etv3 (0.740) Elk3 (0.740) Tcfcp2l1 (0.740) Dmbx1 (0.819) Erf (0.819) Sp2 (0.819) Etv6 (0.819) | BP: RNA splicing CC: transcription factor complex MF: ATP binding MF: zinc ion binding MF: translation regulator activity |
| 56 | 102 |  |  | 0.740 | Egr4 (0.740) | BP: transcription BP: cell division MF: ATP binding MF: transcription factor activity MF: sequence-specific DNA binding |
| 57 | 17 |  |  | 0.747 | Twist1 (0.747) Ctcfl (0.747) Atoh8 (0.747) Ctcf (0.747) Tgif2 (0.747) Snai2 (0.838) Clock (0.838) Neurod1 (0.838) Figla (0.838) Atoh1 (0.838) Creb3l2 (0.838) Max (0.838) Prrx2 (0.838) Mycn (0.838) Hoxd1 (0.838) Tcfap4 (0.838) Nkx3-2 (0.838) Nkx2-3 (0.838) Tlx1 (0.838) T (0.838) Pou6f1 (0.838) Nkx3-1 (0.838) Nkx1-1 (0.838) Cxxc1 (0.838) Nkx2-6 (0.838) E2F3 (0.838) Insm1 (0.838) Rfx3 (0.838) Nkx2-5 (0.838) Lhx4 (0.838) Tcfe2a (0.838) Sox13 (0.838) Osr1 (0.838) Plag1 (0.838) Tcf4 (0.838) Klf6 (0.838) Neurod2 (0.838) Nkx1-1 (0.838) Nrl (0.838) Hoxb3 (0.838) Zfp784 (0.838) Pou6f1 (0.838) Six6 (0.838) Max (0.838) Hoxa9 (0.838) Klf3 (0.838) Tcfe3 (0.838) Npas2 (0.838) Mitf (0.838) Usf2 (0.838) Egr1 (0.838) Nkx2-4 (0.838) Scrt1 (0.838) Gli1 (0.838) Myf6 (0.838) Lhx2 (0.838) Scrt2 (0.838) | BP: transport BP: immune response CC: extracellular region CC: cytoplasmic part MF: binding |
| 58 | 110 |  |  | 0.749 | E2f1 (0.749) Ctcf (0.815) Zfp384 (0.815) | CC: transcription factor complex CC: mitochondrial inner membrane MF: ATP binding MF: zinc ion binding MF: translation regulator activity |
| 59 | 5 |  |  | 0.753 | Sp4 (0.753) Klf2 (0.753) Ctcf (0.753) | BP: transcription CC: Golgi apparatus part MF: transcription factor activity MF: sequence-specific DNA binding MF: transcription activator activity |
| 60 | 86 |  |  | 0.756 | Ipf1 (0.756) Barx1 (0.756) Mnx1 (0.756) Nkx2-9 (0.756) Lhx4 (0.756) Barx2 (0.756) Lhx2 (0.756) Nkx3-1 (0.756) Nkx6-3 (0.885) Nkx2-3 (0.885) | BP: sensory perception of smell BP: G-protein coupled receptor protein signaling pathway BP: signal transduction CC: integral to membrane MF: olfactory receptor activity |
| 61 | 60 |  |  | 0.768 | Srf (0.768) | BP: G-protein coupled receptor protein signaling pathway BP: sensory perception of smell BP: signal transduction CC: integral to membrane MF: olfactory receptor activity |
| 62 | 103 |  |  | 0.839 | Spic (0.839) Egr1 (0.839) Sp3 (0.839) Wt1 (0.839) Gabpa (0.839) E2f3 (0.839) Irf4 (0.839) Etv6 (0.839) E2f1 (0.839) Zbtb4 (0.839) Hivep2 (0.839) Etv5 (0.839) Sp2 (0.839) Elk1 (0.839) Nr2c2 (0.839) Spib (0.839) Irf2 (0.839) Irf6 (0.850) Erf (0.863) Etv1 (0.869) Elk3 (0.869) Gm5454 (0.869) Etv4 (0.869) Elf2 (0.869) Erg (0.869) Pax5 (0.869) Fli1 (0.869) Bcl6b (0.869) Elf4 (0.869) Irf5 (0.869) Pitx3 (0.869) Nhlh1 (0.869) Ybx1 (0.869) Egr1 (0.869) Etv3 (0.869) Elk4 (0.869) Gm4881 (0.869) | BP: cell division BP: RNA splicing MF: ATP binding MF: zinc ion binding MF: transcription activator activity |
| 63 | 23 |  |  | 0.858 | Hmg20b (0.858) | BP: G-protein coupled receptor protein signaling pathway BP: sensory perception of smell BP: signal transduction BP: cell communication MF: olfactory receptor activity |
| 64 | 3 |  |  | NA |  | BP: sensory perception of smell BP: signal transduction BP: cell communication CC: integral to membrane MF: olfactory receptor activity |
| 65 | 6 |  |  | NA |  | CC: mitochondrial inner membrane MF: RNA binding MF: ATP binding MF: GTP binding MF: structural constituent of ribosome |
| 66 | 8 |  |  | NA |  | BP: sensory perception of smell BP: G-protein coupled receptor protein signaling pathway BP: signal transduction CC: integral to membrane MF: olfactory receptor activity |
| 67 | 11 |  |  | NA |  | BP: sensory perception of smell BP: G-protein coupled receptor protein signaling pathway BP: signal transduction BP: cell communication MF: olfactory receptor activity |
| 68 | 12 |  |  | NA |  | BP: sensory perception of smell BP: G-protein coupled receptor protein signaling pathway BP: signal transduction CC: integral to membrane MF: olfactory receptor activity |
| 69 | 14 |  |  | NA |  | BP: sensory perception of smell BP: G-protein coupled receptor protein signaling pathway BP: signal transduction BP: cell communication MF: olfactory receptor activity |
| 70 | 16 |  |  | NA |  | BP: sensory perception of smell BP: G-protein coupled receptor protein signaling pathway BP: signal transduction CC: integral to membrane MF: olfactory receptor activity |
| 71 | 18 |  |  | NA |  | BP: sensory perception of smell BP: immune response MF: olfactory receptor activity |
| 72 | 19 |  |  | NA |  | BP: sensory perception of smell BP: G-protein coupled receptor protein signaling pathway BP: signal transduction CC: integral to membrane MF: olfactory receptor activity |
| 73 | 20 |  |  | NA |  | BP: nucleosome assembly CC: nucleosome CC: mitochondrion CC: nucleus MF: binding |
| 74 | 22 |  |  | NA |  | BP: G-protein coupled receptor protein signaling pathway BP: sensory perception of smell CC: integral to membrane MF: olfactory receptor activity MF: cytokine activity |
| 75 | 24 |  |  | NA |  | BP: transcription BP: translation BP: positive regulation of developmental process BP: cell-cell signaling CC: transcription factor complex |
| 76 | 25 |  |  | NA |  | BP: sensory perception of smell BP: G-protein coupled receptor protein signaling pathway BP: signal transduction CC: integral to membrane MF: olfactory receptor activity |
| 77 | 26 |  |  | NA |  | BP: cell differentiation CC: plasma membrane MF: calcium ion binding MF: transcription factor activity MF: sequence-specific DNA binding |
| 78 | 27 |  |  | NA |  | CC: extracellular space CC: integral to membrane CC: proteinaceous extracellular matrix MF: calcium ion binding MF: cytokine activity |
| 79 | 28 |  |  | NA |  | BP: G-protein coupled receptor protein signaling pathway BP: sensory perception of smell BP: signal transduction BP: cell communication MF: olfactory receptor activity |
| 80 | 32 |  |  | NA |  | BP: sensory perception of smell BP: G-protein coupled receptor protein signaling pathway BP: signal transduction BP: cell communication MF: olfactory receptor activity |
| 81 | 36 |  |  | NA |  | BP: sensory perception of smell BP: G-protein coupled receptor protein signaling pathway BP: signal transduction BP: cell communication MF: olfactory receptor activity |
| 82 | 37 |  |  | NA |  | BP: sensory perception of smell BP: G-protein coupled receptor protein signaling pathway BP: signal transduction CC: integral to membrane MF: olfactory receptor activity |
| 83 | 38 |  |  | NA |  | BP: sensory perception of smell BP: G-protein coupled receptor protein signaling pathway BP: defense response CC: extracellular region MF: olfactory receptor activity |
| 84 | 40 |  |  | NA |  | BP: sensory perception of smell BP: G-protein coupled receptor protein signaling pathway BP: signal transduction BP: cell communication MF: olfactory receptor activity |
| 85 | 41 |  |  | NA |  | BP: sensory perception of smell BP: G-protein coupled receptor protein signaling pathway BP: signal transduction CC: integral to membrane MF: olfactory receptor activity |
| 86 | 44 |  |  | NA |  | BP: G-protein coupled receptor protein signaling pathway BP: oxidation reduction CC: integral to membrane CC: plasma membrane MF: olfactory receptor activity |
| 87 | 45 |  |  | NA |  | BP: translation BP: RNA splicing BP: protein transport CC: spliceosomal complex MF: RNA binding |
| 88 | 46 |  |  | NA |  | BP: sensory perception of smell BP: G-protein coupled receptor protein signaling pathway BP: defense response to bacterium BP: regulation of steroid hormone receptor signaling pathway MF: olfactory receptor activity |
| 89 | 48 |  |  | NA |  | BP: translation BP: mRNA processing BP: mitosis CC: spliceosomal complex MF: RNA binding |
| 90 | 55 |  |  | NA |  | BP: signal transduction BP: G-protein coupled receptor protein signaling pathway CC: extracellular space MF: sequence-specific DNA binding MF: transcription factor activity |
| 91 | 56 |  |  | NA |  | BP: G-protein coupled receptor protein signaling pathway BP: inflammatory response BP: cell communication CC: extracellular space MF: calcium ion binding |
| 92 | 58 |  |  | NA |  | BP: transcription BP: positive regulation of transcription from RNA polymerase II promoter BP: protein amino acid phosphorylation BP: negative regulation of transcription from RNA polymerase II promoter MF: ATP binding |
| 93 | 59 |  |  | NA |  | BP: sensory perception of smell BP: G-protein coupled receptor protein signaling pathway BP: signal transduction BP: cell communication MF: olfactory receptor activity |
| 94 | 62 |  |  | NA |  | BP: defense response BP: oxidation reduction CC: extracellular region CC: extrinsic to plasma membrane MF: oxidoreductase activity |
| 95 | 63 |  |  | NA |  | BP: peptidyl-citrulline biosynthetic process from peptidyl-arginine CC: proteinaceous extracellular matrix CC: extracellular space MF: calcium ion binding MF: protein-arginine deiminase activity |
| 96 | 65 |  |  | NA |  | BP: sensory perception of smell BP: G-protein coupled receptor protein signaling pathway BP: signal transduction CC: integral to membrane MF: olfactory receptor activity |
| 97 | 66 |  |  | NA |  | BP: sensory perception of smell BP: G-protein coupled receptor protein signaling pathway BP: cell communication MF: olfactory receptor activity MF: pheromone receptor activity |
| 98 | 68 |  |  | NA |  | BP: sensory perception of smell BP: G-protein coupled receptor protein signaling pathway BP: signal transduction CC: integral to membrane MF: olfactory receptor activity |
| 99 | 69 |  |  | NA |  | BP: immune response BP: cell adhesion BP: positive regulation of B cell activation CC: extracellular region MF: sugar binding |
| 100 | 70 |  |  | NA |  | BP: sensory perception of smell BP: G-protein coupled receptor protein signaling pathway BP: signal transduction CC: integral to membrane MF: olfactory receptor activity |
| 101 | 71 |  |  | NA |  | BP: sensory perception of smell BP: G-protein coupled receptor protein signaling pathway BP: cell communication CC: integral to membrane MF: olfactory receptor activity |
| 102 | 77 |  |  | NA |  | BP: sensory perception of smell BP: signal transduction BP: cell communication CC: integral to membrane MF: olfactory receptor activity |
| 103 | 78 |  |  | NA |  | BP: sensory perception of smell BP: G-protein coupled receptor protein signaling pathway BP: signal transduction CC: integral to membrane MF: olfactory receptor activity |
| 104 | 80 |  |  | NA |  | BP: G-protein coupled receptor protein signaling pathway BP: sensory perception of smell BP: signal transduction CC: integral to membrane MF: olfactory receptor activity |
| 105 | 87 |  |  | NA |  | BP: negative regulation of transcription from RNA polymerase II promoter CC: transcription factor complex MF: ATP binding MF: zinc ion binding MF: transcription activator activity |
| 106 | 90 |  |  | NA |  | BP: transcription BP: protein amino acid phosphorylation MF: transcription factor activity MF: ATP binding MF: zinc ion binding |
| 107 | 92 |  |  | NA |  | BP: sensory perception of smell BP: G-protein coupled receptor protein signaling pathway BP: signal transduction CC: integral to membrane MF: olfactory receptor activity |
| 108 | 93 |  |  | NA |  | BP: sodium ion transport CC: keratin filament MF: calcium ion binding MF: sodium ion binding MF: sodium channel activity |
| 109 | 94 |  |  | NA |  | BP: transcription CC: mitochondrial inner membrane MF: transcription factor activity MF: ATP binding MF: sequence-specific DNA binding |
| 110 | 95 |  |  | NA |  | BP: G-protein coupled receptor protein signaling pathway BP: sensory perception of smell BP: signal transduction CC: integral to membrane MF: olfactory receptor activity |
| 111 | 96 |  |  | NA |  | BP: sensory perception of smell BP: G-protein coupled receptor protein signaling pathway CC: integral to membrane CC: extracellular space MF: olfactory receptor activity |
| 112 | 97 |  |  | NA |  | BP: sensory perception of smell BP: G-protein coupled receptor protein signaling pathway BP: signal transduction BP: cell communication MF: olfactory receptor activity |
| 113 | 99 |  |  | NA |  | CC: extracellular region CC: myosin complex MF: serine-type peptidase activity MF: actin binding MF: endopeptidase inhibitor activity |
| 114 | 100 |  |  | NA |  | BP: sensory perception of smell BP: G-protein coupled receptor protein signaling pathway BP: signal transduction CC: integral to membrane MF: olfactory receptor activity |
| 115 | 106 |  |  | NA |  | BP: gene expression BP: oxidation reduction CC: nucleosome CC: male germ cell nucleus MF: binding |
| 116 | 107 |  |  | NA |  | BP: response to external stimulus CC: integral to membrane CC: myosin complex MF: serine-type endopeptidase activity MF: heme binding |
| 117 | 109 |  |  | NA |  | BP: sensory perception of smell BP: G-protein coupled receptor protein signaling pathway BP: defense response to bacterium CC: integral to membrane MF: olfactory receptor activity |
| 118 | 112 |  |  | NA |  | BP: nucleosome assembly BP: innate immune response CC: nucleosome CC: integral to membrane MF: serine-type endopeptidase activity |
| 119 | 113 |  |  | NA |  | BP: sensory perception of smell BP: G-protein coupled receptor protein signaling pathway BP: signal transduction CC: integral to membrane MF: olfactory receptor activity |
| 120 | 115 |  |  | NA |  | BP: regulation of production of small RNA involved in gene silencing by RNA BP: innate immune response CC: integral to membrane MF: serine-type endopeptidase activity MF: serine-type endopeptidase inhibitor activity |
| 121 | 116 |  |  | NA |  | BP: sensory perception of smell BP: G-protein coupled receptor protein signaling pathway BP: signal transduction CC: integral to membrane MF: olfactory receptor activity |
| 122 | 117 |  |  | NA |  | BP: sensory perception of smell BP: G-protein coupled receptor protein signaling pathway BP: signal transduction CC: integral to membrane MF: olfactory receptor activity |
| 123 | 119 |  |  | NA |  | BP: sensory perception of smell BP: G-protein coupled receptor protein signaling pathway BP: signal transduction CC: integral to membrane MF: olfactory receptor activity |
| 124 | 120 |  |  | NA |  | BP: sensory perception of smell BP: G-protein coupled receptor protein signaling pathway BP: signal transduction BP: cell communication MF: olfactory receptor activity |
| 125 | 122 |  |  | NA |  | BP: G-protein coupled receptor protein signaling pathway BP: sensory perception of smell BP: signal transduction CC: integral to membrane MF: olfactory receptor activity |
| 126 | 123 |  |  | NA |  | BP: G-protein coupled receptor protein signaling pathway BP: sensory perception of smell BP: signal transduction CC: integral to membrane MF: olfactory receptor activity |
| 127 | 124 |  |  | NA |  | BP: cell division BP: mitosis CC: spliceosomal complex CC: mitochondrial inner membrane MF: ATP binding |
| 128 | 125 |  |  | NA |  | BP: sensory perception of smell BP: G-protein coupled receptor protein signaling pathway BP: signal transduction BP: cell communication MF: olfactory receptor activity |
