# Supplementary material for: DeepCpG: accurate prediction of single-cell DNA methylation states using deep learning
Source: Genome Biol. 2017 Apr 11;18:67. doi: 10.1186/s13059-017-1189-z (PMC5387360; doi:10.1186/s13059-017-1189-z)
Supplement: Supplementary file 3 — Sequence motifs. HTML files with sequence logos and summary statistics for all cell types. (ZIP 19482 kb) [file 13059_2017_1189_MOESM3_ESM.zip › HCC.html]

Filter table


# Filter table

#### *Christof Angermueller*

#### *2016-12-17*

Nr | Label | Logo | Influence | q-value | Related known motifs | GO annotations || 1 | 70: SP3 |  |  | 0.001 | SP3 (0.001) EGR1 (0.008) SP4 (0.022) ZNF148 (0.048) ZBTB7B (0.048) SP2 (0.053) KLF6 (0.053) ZNF281 (0.053) EGR2 (0.053) WT1 (0.053) E2F1 (0.071) KLF5 (0.074) SP1 (0.074) ZNF740 (0.074) KLF7 (0.074) KLF4 (0.077) KLF12 (0.077) EGR4 (0.077) ARNT2 (0.077) PURA (0.077) KLF2 (0.081) RREB1 (0.088) MAZ (0.088) SP5 (0.088) ZNF219 (0.140) | CC: cytosol CC: transcription factor complex CC: nucleolus MF: ATP binding MF: magnesium ion binding |
| 2 | 108: ZSCAN4 |  |  | 0.002 | ZSCAN4 (0.002) KLF8 (0.684) SOX10 (0.684) KLF1 (0.684) | BP: sensory perception of smell BP: G-protein coupled receptor protein signaling pathway BP: response to external stimulus CC: extracellular space MF: olfactory receptor activity |
| 3 | 93: KLF6 |  |  | 0.005 | KLF6 (0.005) SP1 (0.018) ZNF148 (0.021) SP3 (0.031) WT1 (0.031) ZNF281 (0.031) SP2 (0.031) EGR4 (0.031) DNMT1 (0.058) EGR1 (0.058) MAZ (0.075) KLF5 (0.086) E2F6 (0.098) KLF16 (0.114) SP4 (0.144) ZBTB7B (0.216) SP5 (0.226) ZBTB7A (0.232) ZFY (0.245) TFAP2C (0.277) ZNF263 (0.295) MBD2 (0.325) ZNF740 (0.328) PURA (0.328) SP8 (0.328) ZIC1 (0.328) KLF14 (0.328) KLF4 (0.328) SMARCC1 (0.328) | BP: negative regulation of signal transduction BP: embryonic limb morphogenesis CC: transcription factor complex MF: transcription activator activity MF: GTPase activity |
| 4 | 49: HINFP |  |  | 0.006 | HINFP (0.006) CGBP (0.457) | CC: nucleolus CC: cytosol CC: transcription factor complex MF: ATP binding MF: zinc ion binding |
| 5 | 126: E2F6 |  |  | 0.014 | E2F6 (0.014) TFDP1 (0.042) E2F7 (0.066) E2F4 (0.125) E2F2 (0.148) E2F8 (0.162) MZF1 (0.167) MLL (0.347) ZNF263 (0.504) ZBTB7B (0.504) MAZ (0.817) ZNF202 (0.817) NR0B1 (0.817) HLTF (0.817) MECP2 (0.817) E2F5 (0.817) ZSCAN10 (0.817) ETV2 (0.817) BCL11A (0.817) SP1 (0.817) RBPJ (0.817) E2F3 (0.817) ETS2 (0.817) SP2 (0.817) NR1I3 (0.817) TBR1 (0.817) ENSG00000235187 (0.817) MBD2 (0.817) IRF8 (0.817) ZNF350 (0.817) ZNF148 (0.817) ZNF75A (0.817) | BP: axon guidance CC: transcription factor complex CC: nucleolus MF: chromatin binding MF: RNA polymerase II transcription factor activity, enhancer binding |
| 6 | 30: ZNF740 |  |  | 0.017 | ZNF740 (0.017) MAZ (0.049) ZNF202 (0.049) ZNF148 (0.192) KLF6 (0.192) ZNF219 (0.192) ZNF281 (0.192) MZF1 (0.192) SP2 (0.192) PLAGL1 (0.192) SP1 (0.227) SP3 (0.227) SP4 (0.275) KLF5 (0.315) EGR1 (0.357) ZIC1 (0.357) KLF15 (0.357) INSM1 (0.357) SMARCC1 (0.357) ZIC2 (0.357) ZBTB7B (0.357) KLF2 (0.357) PURA (0.357) GLIS3 (0.357) NR0B1 (0.357) YY2 (0.357) ARNT2 (0.357) ZNF263 (0.357) HIC2 (0.357) GLIS1 (0.357) SREBF1 (0.357) | BP: negative regulation of signal transduction BP: axon guidance BP: potassium ion transport CC: transcription factor complex MF: potassium ion binding |
| 7 | 35: SP3 |  |  | 0.017 | SP3 (0.017) EGR2 (0.206) WT1 (0.225) E2F1 (0.225) SP2 (0.225) EGR1 (0.257) SP4 (0.380) ZBTB7B (0.596) KLF6 (0.596) ZNF219 (0.892) | BP: negative regulation of signal transduction CC: transcription factor complex CC: cytosol MF: ATP binding MF: zinc ion binding |
| 8 | 75: TFAP2D |  |  | 0.022 | TFAP2D (0.022) | BP: negative regulation of signal transduction BP: axon guidance CC: cytosol MF: ATP binding MF: zinc ion binding |
| 9 | 1: ZFY |  |  | 0.023 | ZFY (0.023) ZFX (0.091) ZBTB49 (0.588) SP3 (0.610) KLF12 (0.610) SP5 (0.610) ZKSCAN3 (0.610) EGR1 (0.610) NR0B1 (0.610) DNMT1 (0.622) KLF7 (0.622) WT1 (0.622) SP8 (0.699) SP1 (0.699) KLF2 (0.699) ARNT2 (0.699) KLF5 (0.699) TFAP2B (0.710) SMAD4 (0.710) KLF4 (0.710) TFAP2D (0.710) SP4 (0.710) EGR2 (0.710) PLAGL1 (0.710) MECP2 (0.710) USF2 (0.710) | BP: axon guidance BP: actin cytoskeleton organization CC: transcription factor complex CC: cytosol MF: ATP binding |
| 10 | 43: SP4 |  |  | 0.023 | SP4 (0.023) ZFX (0.110) THRB (0.110) MXI1 (0.110) USF2 (0.110) ESR1 (0.110) CTCFL (0.135) CTCF (0.135) SNAI2 (0.145) CREB3L2 (0.174) EGR4 (0.187) SNAI1 (0.187) MYCL1 (0.188) WT1 (0.214) MBD2 (0.233) ARNTL (0.267) HEY2 (0.290) RARB (0.316) ZFY (0.318) ATF3 (0.374) SP3 (0.374) TFEB (0.374) ID4 (0.374) MYC (0.404) KLF15 (0.404) TWIST1 (0.404) | BP: anterior/posterior pattern formation BP: Wnt receptor signaling pathway, calcium modulating pathway BP: negative regulation of signal transduction MF: transcription factor activity MF: transcription repressor activity |
| 11 | 89: EGR1 |  |  | 0.034 | EGR1 (0.034) ZNF281 (0.034) ZNF148 (0.034) ZNF740 (0.034) GLIS3 (0.042) RREB1 (0.048) SP4 (0.048) WT1 (0.053) SP3 (0.082) ZBTB7B (0.091) KLF15 (0.091) GLIS1 (0.091) PURA (0.091) MAZ (0.091) EGR4 (0.099) KLF5 (0.099) EGR2 (0.111) SP8 (0.139) ZBTB1 (0.139) ZNF524 (0.141) KLF4 (0.141) KLF2 (0.141) SP2 (0.141) E2F3 (0.148) KLF16 (0.149) GLI2 (0.149) ZIC5 (0.149) ZNF219 (0.149) SP1 (0.149) KLF1 (0.149) | BP: negative regulation of signal transduction BP: axon guidance BP: spleen development CC: transcription factor complex MF: potassium ion binding |
| 12 | 102: ELK4 |  |  | 0.038 | ELK4 (0.038) DNMT1 (0.308) GABPA (0.308) ETV6 (0.308) E2F1 (0.308) SP3 (0.308) ETV3 (0.308) ERF (0.308) CTCFL (0.308) ZFX (0.308) TFAP4 (0.308) MBD2 (0.308) FLI1 (0.308) ETS2 (0.341) EGR4 (0.341) WT1 (0.341) ZFY (0.341) ELK3 (0.341) EGR2 (0.341) ERG (0.419) ELF4 (0.419) ETV1 (0.425) ELK1 (0.430) ZBTB4 (0.481) MAFB (0.543) SP4 (0.543) KLF6 (0.543) ZBTB3 (0.543) CREM (0.543) TFAP2D (0.543) TCF12 (0.543) CTCF (0.543) MYOG (0.543) MYOD1 (0.543) | CC: transcription factor complex CC: nucleolus CC: cytosol MF: zinc ion binding MF: ATP binding |
| 13 | 2: SP1 |  |  | 0.050 | SP1 (0.050) EGR1 (0.050) KLF14 (0.050) SP4 (0.050) SP3 (0.050) ZFY (0.050) DNMT1 (0.050) EGR4 (0.050) KLF7 (0.050) ZBTB7B (0.050) SP2 (0.050) SP8 (0.050) KLF12 (0.052) WT1 (0.053) KLF16 (0.053) KLF6 (0.059) EGR2 (0.059) ZFX (0.066) KLF4 (0.091) KLF2 (0.105) E2F1 (0.107) KLF5 (0.107) ARNT2 (0.115) SP5 (0.181) ZFP161 (0.203) HES1 (0.203) TFAP2B (0.203) | CC: cytosol CC: nucleolus CC: transcription factor complex MF: ATP binding MF: zinc ion binding |
| 14 | 124 |  |  | 0.058 | ATF1 (0.058) CREB1 (0.146) CREM (0.164) E4F1 (0.313) CREB3L2 (0.347) ATF6 (0.372) XBP1 (0.475) NR2C2 (0.475) RXRB (0.475) ATF3 (0.475) THRA (0.475) RARA (0.475) USF1 (0.568) NR2E1 (0.568) CBFB (0.568) RXRA (0.568) USF2 (0.568) ATF2 (0.568) CREB3L1 (0.568) RARG (0.568) CREB3 (0.591) ESR1 (0.591) VDR (0.591) TBX21 (0.638) CREB5 (0.748) TGIF1 (0.748) NR4A3 (0.748) | BP: nuclear mRNA splicing, via spliceosome CC: nucleolus CC: spliceosomal complex MF: ATP binding MF: translation regulator activity |
| 15 | 106 |  |  | 0.070 | TFAP2D (0.070) TFAP2B (0.146) ZFX (0.204) MECP2 (0.204) ZFY (0.222) HSF1 (0.222) NR2C1 (0.243) NFIA (0.336) CTCFL (0.358) TFAP2C (0.427) ZIC5 (0.427) DNMT1 (0.427) SP4 (0.443) TFAP2E (0.457) SP3 (0.457) NFIX (0.558) GLIS2 (0.576) ATF3 (0.576) USF1 (0.609) ZIC4 (0.609) ZBTB7A (0.666) TFAP2A (0.666) NFIB (0.666) CTCF (0.709) ARNTL (0.709) SP1 (0.709) TWIST1 (0.709) ELF4 (0.709) USF2 (0.709) MYC (0.709) MYCL1 (0.709) MYCN (0.709) NEUROD1 (0.709) KLF8 (0.709) HES1 (0.709) GLIS3 (0.709) MNT (0.709) MBD2 (0.709) | BP: negative regulation of signal transduction CC: transcription factor complex CC: cytosol MF: zinc ion binding MF: ATP binding |
| 16 | 50 |  |  | 0.074 | ETV6 (0.074) ETV2 (0.441) ENSG00000235187 (0.441) ELF1 (0.580) ELF3 (0.729) ZNF263 (0.761) SPIB (0.761) SPIC (0.858) BCL11A (0.858) RFX5 (0.858) ATF5 (0.858) SP1 (0.858) PAX6 (0.858) IRF3 (0.858) SCRT2 (0.858) ELK4 (0.858) ETV7 (0.881) IRF8 (0.881) SCRT1 (0.886) | BP: positive regulation of transcription from RNA polymerase II promoter BP: negative regulation of cell migration CC: axon MF: transcription factor activity MF: sequence-specific DNA binding |
| 17 | 115 |  |  | 0.082 | REST (0.082) ZNF691 (0.666) | BP: mating BP: cell-cell signaling CC: integral to membrane CC: plasma membrane part CC: extracellular region part |
| 18 | 101 |  |  | 0.101 | KLF7 (0.101) NRF1 (0.101) KLF12 (0.113) KLF14 (0.113) KLF2 (0.143) KLF4 (0.143) KLF1 (0.290) TFAP2C (0.290) SP5 (0.290) YBX1 (0.290) SP8 (0.290) KLF16 (0.290) KLF5 (0.340) SMAD3 (0.388) EGR2 (0.486) MAX (0.507) KLF13 (0.507) ZFX (0.507) SP3 (0.507) SP1 (0.519) TFAP2A (0.539) BHLHE41 (0.539) NEUROD1 (0.539) PURA (0.539) KLF3 (0.542) | BP: regulation of signal transduction BP: cell development MF: transcription factor activity MF: sequence-specific DNA binding MF: protein domain specific binding |
| 19 | 87 |  |  | 0.107 | DNMT1 (0.107) TFAP2B (0.107) CTCFL (0.107) ZFX (0.169) TFAP2D (0.169) MECP2 (0.169) EGR4 (0.169) PLAGL1 (0.169) MBD2 (0.169) ZIC5 (0.169) SP4 (0.313) SP3 (0.403) EBF1 (0.403) GLIS2 (0.412) CTCF (0.412) E2F1 (0.472) ETS1 (0.472) ATF6 (0.472) ZFY (0.490) SNAI1 (0.490) WT1 (0.514) SMARCC2 (0.532) NRF1 (0.555) ZIC4 (0.555) EGR1 (0.555) SP1 (0.555) ELK3 (0.555) | CC: transcription factor complex CC: cytosol CC: nucleolus MF: zinc ion binding MF: ATP binding |
| 20 | 74 |  |  | 0.108 | HMG20B (0.108) FOXJ2 (0.108) CPEB1 (0.108) FOXN3 (0.135) MSX1 (0.135) FOXJ1 (0.135) VAX1 (0.135) FOXF1 (0.135) MNX1 (0.135) LHX1 (0.135) FOXO3 (0.135) VAX2 (0.135) HOXD12 (0.135) HOXC12 (0.135) FOXJ3 (0.136) LHX5 (0.137) ARID3B (0.137) FOXP2 (0.139) ONECUT3 (0.171) PROP1 (0.171) FOXB2 (0.171) ZFHX3 (0.171) FOXL2 (0.171) ARID3C (0.186) FOXK2 (0.194) FOXA1 (0.194) HNF1A (0.194) FOXQ1 (0.194) FOXD2 (0.194) | BP: sensory perception of smell BP: G-protein coupled receptor protein signaling pathway BP: DNA damage checkpoint CC: condensed chromosome kinetochore MF: olfactory receptor activity |
| 21 | 26 |  |  | 0.130 | MAF (0.130) NRL (0.216) MAFK (0.361) CIC (0.361) MAFB (0.361) | BP: anterior/posterior pattern formation CC: nucleolus MF: RNA binding MF: transcription factor activity MF: sequence-specific DNA binding |
| 22 | 94 |  |  | 0.132 | KLF6 (0.132) ZBTB7B (0.164) MAZ (0.164) E2F1 (0.232) ZNF263 (0.232) SP3 (0.232) SP2 (0.232) WT1 (0.232) ZNF219 (0.232) SP4 (0.232) SP1 (0.234) ARNT2 (0.234) ZIC2 (0.234) ZIC1 (0.295) ZNF148 (0.327) MBD2 (0.327) ZNF350 (0.393) ZFX (0.468) EGR1 (0.487) ZNF740 (0.500) ZNF281 (0.512) PLAGL1 (0.519) ZNF691 (0.594) KLF15 (0.760) NR0B1 (0.763) | BP: negative regulation of signal transduction CC: transcription factor complex MF: transcription activator activity MF: zinc ion binding MF: GTPase activity |
| 23 | 3 |  |  | 0.141 | FOXN2 (0.141) FOXP1 (0.509) FOXJ3 (0.509) HNF1B (0.619) FOXO1 (0.619) SRY (0.619) MEF2A (0.619) ONECUT3 (0.619) FUBP1 (0.619) MEF2C (0.619) ZNF35 (0.713) ONECUT1 (0.746) GATA6 (0.746) TCF7L1 (0.746) CPEB1 (0.746) GATA4 (0.747) FOXF1 (0.787) HNF1A (0.796) IRF1 (0.829) ONECUT2 (0.829) HMX1 (0.829) ZFHX3 (0.878) LHX3 (0.878) HMGA1 (0.878) FOXF2 (0.878) HOXC13 (0.878) FOXD3 (0.878) SOX5 (0.878) SOX1 (0.878) FOXJ2 (0.878) HMX3 (0.878) | BP: sensory perception of smell BP: cellular macromolecule biosynthetic process BP: RNA splicing MF: olfactory receptor activity MF: RNA binding |
| 24 | 122 |  |  | 0.147 | PLAGL1 (0.147) MBD2 (0.335) | BP: anterior/posterior pattern formation CC: nucleolus CC: cytosol MF: ATP binding MF: zinc ion binding |
| 25 | 58 |  |  | 0.149 | TFAP2D (0.149) MECP2 (0.724) GLIS2 (0.724) TFAP2B (0.724) | CC: cytosol CC: nucleolus MF: transcription activator activity MF: zinc ion binding MF: ATP binding |
| 26 | 42 |  |  | 0.168 | MECP2 (0.168) ELF1 (0.199) ETV6 (0.199) ELF4 (0.199) HSF1 (0.260) WT1 (0.304) TFAP2B (0.319) SP4 (0.319) SP1 (0.319) SP2 (0.319) E2F6 (0.319) ELK4 (0.319) GABPA (0.319) ELK1 (0.319) MAZ (0.319) MBD2 (0.319) EGR1 (0.326) ZNF263 (0.335) SP3 (0.335) KLF15 (0.363) ZNF148 (0.363) MLL (0.397) ENSG00000235187 (0.397) NR5A2 (0.425) ARNTL (0.425) | CC: nucleolus CC: cytosol MF: transcription factor activity MF: sequence-specific DNA binding MF: zinc ion binding |
| 27 | 119 |  |  | 0.170 | MYPOP (0.170) E2F2 (0.224) E2F4 (0.324) | BP: nuclear mRNA splicing, via spliceosome CC: nucleolus CC: spliceosomal complex MF: transcription factor activity MF: RNA polymerase II transcription factor activity |
| 28 | 46 |  |  | 0.193 | TBX21 (0.193) RXRB (0.193) | BP: sensory perception of smell BP: G-protein coupled receptor protein signaling pathway BP: defense response to bacterium CC: integral to membrane MF: olfactory receptor activity |
| 29 | 95 |  |  | 0.199 | ARNT2 (0.199) USF2 (0.378) SP4 (0.378) ZNF219 (0.378) HINFP (0.378) KLF6 (0.378) HES1 (0.378) GLIS1 (0.378) WT1 (0.504) ZNF740 (0.537) SP3 (0.537) SP1 (0.546) TFAP2D (0.567) ZNF148 (0.658) CREB3L2 (0.658) E2F2 (0.658) E2F1 (0.658) PLAGL1 (0.658) EGR1 (0.658) GLIS2 (0.658) ZNF263 (0.658) GLIS3 (0.658) ZNF281 (0.658) HESX1 (0.658) MYCL1 (0.658) SP2 (0.658) SMAD3 (0.658) | BP: negative regulation of signal transduction CC: nucleolus CC: cytosol MF: translation regulator activity MF: ATP binding |
| 30 | 76 |  |  | 0.202 | E2F2 (0.202) E2F3 (0.248) TFDP1 (0.498) E2F4 (0.498) GLIS2 (0.498) CGBP (0.498) E2F5 (0.498) E2F8 (0.822) | CC: nucleolus CC: transcription factor complex CC: spliceosomal complex CC: cytosol MF: ATP binding |
| 31 | 64 |  |  | 0.214 | E2F1 (0.214) EGR2 (0.216) MBD2 (0.494) ZBTB7B (0.564) MNT (0.564) ESR2 (0.564) ZFY (0.564) ESR1 (0.564) WT1 (0.564) E2F6 (0.564) EGR1 (0.564) DNMT1 (0.564) SP4 (0.564) ZFX (0.587) ZIC4 (0.690) GLIS3 (0.690) CTCFL (0.690) TFAP2E (0.737) SOHLH2 (0.746) ZBTB4 (0.844) SP3 (0.844) TFAP2D (0.875) KLF6 (0.899) ZNF281 (0.899) TERF2 (0.899) | BP: negative regulation of signal transduction CC: transcription factor complex CC: nucleolus CC: cytosol MF: ATP binding |
| 32 | 16 |  |  | 0.230 | PURA (0.230) | BP: anterior/posterior pattern formation CC: integral to plasma membrane MF: sequence-specific DNA binding MF: transcription factor activity MF: calcium ion binding |
| 33 | 77 |  |  | 0.240 | MECP2 (0.240) E2F6 (0.240) NR0B1 (0.240) CTCFL (0.240) TFAP2B (0.240) EBF1 (0.240) SP4 (0.323) EGR1 (0.323) TFAP2C (0.323) TFAP2D (0.350) ZBTB7B (0.363) CTCF (0.363) ARNT2 (0.611) ETS1 (0.611) GABPA (0.611) PLAGL1 (0.611) INSM1 (0.611) ERF (0.611) ETV3 (0.611) MBD2 (0.611) PLAG1 (0.611) KLF15 (0.611) SP2 (0.611) ZIC4 (0.611) ZNF350 (0.611) SMARCC2 (0.611) ZBTB7A (0.611) | CC: nucleolus CC: cytosol MF: zinc ion binding MF: ubiquitin-protein ligase activity MF: ATP binding |
| 34 | 97 |  |  | 0.242 | HOXA1 (0.242) PBX1 (0.432) HOXB1 (0.432) PBX2 (0.432) FOXH1 (0.657) | BP: sensory perception of smell BP: G-protein coupled receptor protein signaling pathway BP: visual perception CC: extracellular region MF: olfactory receptor activity |
| 35 | 4 |  |  | 0.255 | NRF1 (0.255) | BP: nuclear mRNA splicing, via spliceosome CC: nucleolus CC: transcription factor complex MF: ATP binding MF: zinc ion binding |
| 36 | 88 |  |  | 0.262 | HNF4A (0.262) | BP: immune response CC: extracellular region |
| 37 | 10 |  |  | 0.264 | RFX2 (0.264) RFX4 (0.314) NFIC (0.337) RFX3 (0.584) TP73 (0.601) CREB3L1 (0.601) RFX6 (0.724) | BP: associative learning BP: positive regulation of immune response BP: glucose catabolic process CC: extracellular space MF: carbohydrate kinase activity |
| 38 | 5 |  |  | 0.297 | SMARCC2 (0.297) ZNF143 (0.297) ELK4 (0.340) ELK1 (0.506) ETS1 (0.521) KLF15 (0.521) | BP: Wnt receptor signaling pathway CC: nucleolus CC: cytosol MF: zinc ion binding MF: ubiquitin-protein ligase activity |
| 39 | 28 |  |  | 0.298 | NKX2-5 (0.298) GABPA (0.638) ETV3 (0.726) ERF (0.726) FLI1 (0.726) ETV1 (0.726) NKX2-8 (0.726) ELK1 (0.832) IRF8 (0.832) TCF12 (0.832) NKX2-3 (0.832) ZBTB3 (0.832) ELK4 (0.832) IRF5 (0.832) ETV4 (0.832) SNAI2 (0.832) ETV6 (0.851) ELF1 (0.851) ELK3 (0.851) ETV7 (0.870) NKX2-1 (0.888) SCRT1 (0.888) SCRT2 (0.889) | BP: sensory perception of smell BP: G-protein coupled receptor protein signaling pathway CC: integral to membrane MF: olfactory receptor activity |
| 40 | 84 |  |  | 0.350 | CTCFL (0.350) MBD2 (0.350) ZIC4 (0.350) DNMT1 (0.350) EGR1 (0.350) WT1 (0.350) MECP2 (0.350) SP4 (0.350) ZBTB7B (0.350) ZIC5 (0.350) CTCF (0.395) ZNF263 (0.546) GLIS2 (0.546) EGR2 (0.546) TFAP2E (0.546) EGR4 (0.546) ATF6 (0.546) SMARCC2 (0.546) TFAP2B (0.546) ETS1 (0.546) MAFB (0.546) ELF2 (0.546) ZNF740 (0.546) KLF15 (0.546) RFX1 (0.546) E2F8 (0.546) ZFY (0.546) SPDEF (0.546) KLF8 (0.546) E2F4 (0.546) ETV6 (0.546) SP1 (0.546) GABPA (0.546) ARNT2 (0.546) GLIS3 (0.546) EBF1 (0.546) | BP: negative regulation of signal transduction BP: negative regulation of gene-specific transcription from RNA polymerase II promoter CC: transcription factor complex MF: transcription activator activity MF: potassium ion binding |
| 41 | 82 |  |  | 0.372 | MBD2 (0.372) MECP2 (0.780) | BP: anterior/posterior pattern formation CC: nucleolus CC: cytosol CC: transcription factor complex MF: ATP binding |
| 42 | 99 |  |  | 0.393 | CREB3 (0.393) ATF7 (0.393) ZIC3 (0.393) CBFB (0.393) SREBF2 (0.393) SREBF1 (0.393) RORB (0.393) BATF3 (0.393) PRDM4 (0.393) ZIC5 (0.393) CTCF (0.393) RARG (0.405) CREB5 (0.476) VDR (0.496) RXRB (0.496) GLIS3 (0.537) CREB1 (0.537) ZIC2 (0.582) ESRRA (0.582) ZIC1 (0.582) RUNX1 (0.640) RARA (0.640) PLAG1 (0.640) ARID2 (0.640) HIC2 (0.640) | BP: peptidyl-citrulline biosynthetic process from peptidyl-arginine MF: sequence-specific DNA binding MF: transcription factor activity MF: potassium ion binding MF: protein-arginine deiminase activity |
| 43 | 14 |  |  | 0.399 | POU2F2 (0.399) POU3F3 (0.399) POU3F4 (0.594) POU2F3 (0.594) POU5F1B (0.594) POU2F1 (0.594) FOXO6 (0.594) DMRTC2 (0.594) | BP: sensory perception of smell BP: G-protein coupled receptor protein signaling pathway BP: defense response to bacterium CC: integral to membrane MF: olfactory receptor activity |
| 44 | 25 |  |  | 0.413 | GATA2 (0.413) GATA4 (0.413) GATA1 (0.868) | BP: sensory perception of smell BP: G-protein coupled receptor protein signaling pathway BP: immune response MF: olfactory receptor activity MF: serine-type endopeptidase inhibitor activity |
| 45 | 7 |  |  | 0.418 | EPAS1 (0.418) | BP: axon guidance BP: protein homooligomerization BP: neurotransmitter transport BP: regulation of heart contraction CC: extracellular space |
| 46 | 105 |  |  | 0.425 | SP3 (0.425) TFAP2D (0.425) SP4 (0.425) ZNF219 (0.425) SP1 (0.425) SP2 (0.425) E2F1 (0.425) ZBTB7A (0.425) KLF16 (0.425) EGR1 (0.425) WT1 (0.425) DNMT1 (0.425) KLF5 (0.431) KLF4 (0.554) KLF12 (0.554) EGR4 (0.554) KLF7 (0.554) KLF2 (0.563) SP5 (0.578) ZFX (0.652) SP8 (0.652) ZFY (0.652) ZBTB7B (0.652) PLAGL1 (0.652) GLIS3 (0.864) | CC: nucleolus CC: cytosol CC: transcription factor complex MF: ATP binding MF: magnesium ion binding |
| 47 | 125 |  |  | 0.439 | SMAD1 (0.439) SMARCC1 (0.519) RARG (0.519) MAF (0.519) | BP: associative learning BP: inner ear morphogenesis MF: sequence-specific DNA binding MF: transcription factor activity MF: potassium ion binding |
| 48 | 59 |  |  | 0.447 | ARID3B (0.447) ONECUT1 (0.447) ONECUT3 (0.596) FOXP2 (0.596) HOXC6 (0.596) FOXN2 (0.596) FOXP1 (0.596) POU1F1 (0.596) HOMEZ (0.596) LHX3 (0.596) FOXQ1 (0.596) NKX3-1 (0.596) DMRTC2 (0.596) FOXO1 (0.596) HMG20B (0.596) FOXJ1 (0.596) FOXF1 (0.596) DMRT1 (0.596) POU3F2 (0.596) SOX1 (0.596) POU4F1 (0.596) DMRTA2 (0.596) CPEB1 (0.596) TBP (0.615) SRF (0.615) | BP: sensory perception of smell BP: G-protein coupled receptor protein signaling pathway BP: gene expression BP: DNA damage checkpoint MF: olfactory receptor activity |
| 49 | 40 |  |  | 0.449 | E2F6 (0.449) MAZ (0.486) DNMT1 (0.486) E2F4 (0.486) ZNF263 (0.486) SP1 (0.543) MBD2 (0.543) EGR1 (0.543) EGR4 (0.573) E2F8 (0.602) KLF6 (0.602) ETS2 (0.602) E2F2 (0.668) ZBTB7B (0.668) NR0B1 (0.668) SP2 (0.686) ZFY (0.686) TFDP1 (0.686) THAP1 (0.686) ZNF148 (0.686) TFAP2B (0.686) KLF15 (0.686) TFAP2E (0.686) SP3 (0.686) ZNF350 (0.686) KLF5 (0.686) | BP: potassium ion transport BP: negative regulation of neuron differentiation CC: transcription factor complex MF: RNA polymerase II transcription factor activity, enhancer binding MF: promoter binding |
| 50 | 78 |  |  | 0.463 | RARB (0.463) TFAP2C (0.463) RARA (0.668) THAP1 (0.668) RXRB (0.668) | BP: regulation of transport BP: alcohol metabolic process BP: muscle contraction CC: extracellular region MF: calcium ion binding |
| 51 | 85 |  |  | 0.529 | NKX2-1 (0.529) SMAD1 (0.529) | BP: water transport MF: sequence-specific DNA binding MF: structural constituent of eye lens MF: calcium ion binding MF: transcription factor activity |
| 52 | 12 |  |  | 0.586 | EOMES (0.586) STAT3 (0.586) FEV (0.586) | BP: tissue development BP: positive regulation of transcription from RNA polymerase II promoter MF: transcription factor activity MF: sequence-specific DNA binding MF: inward rectifier potassium channel activity |
| 53 | 86 |  |  | 0.658 | PAX5 (0.658) ZFX (0.888) | BP: negative regulation of signal transduction BP: protein amino acid phosphorylation CC: transcription factor complex CC: cytosol MF: ATP binding |
| 54 | 29 |  |  | 0.672 | PITX2 (0.672) CRX (0.672) PITX3 (0.672) DUX4 (0.672) PITX1 (0.672) DUXA (0.722) ISX (0.722) ALX1 (0.722) PHOX2B (0.722) DRGX (0.722) PHOX2A (0.722) | BP: sensory perception of smell BP: G-protein coupled receptor protein signaling pathway MF: olfactory receptor activity MF: eukaryotic cell surface binding MF: serine-type endopeptidase inhibitor activity |
| 55 | 121 |  |  | 0.682 | EGR4 (0.682) TFAP4 (0.682) SP4 (0.682) DNMT1 (0.682) MBD2 (0.682) ZBTB4 (0.682) ZBTB7B (0.682) KLF8 (0.682) KLF15 (0.691) | BP: negative regulation of signal transduction CC: transcription factor complex CC: nucleolus CC: cytosol MF: zinc ion binding |
| 56 | 11 |  |  | 0.694 | NHLH1 (0.694) | BP: axon guidance BP: negative regulation of signal transduction CC: nucleolus MF: transcription activator activity MF: ATP binding |
| 57 | 90 |  |  | 0.696 | HINFP (0.696) | BP: negative regulation of signal transduction CC: nucleolus CC: cytosol MF: RNA binding MF: zinc ion binding |
| 58 | 118 |  |  | 0.742 | ARNT2 (0.742) ETV6 (0.832) KLF6 (0.832) CTCFL (0.832) NR0B1 (0.832) | BP: negative regulation of signal transduction CC: transcription factor complex CC: cytosol MF: zinc ion binding MF: ATP binding |
| 59 | 44 |  |  | 0.746 | SMAD1 (0.746) SMAD3 (0.753) E2F6 (0.868) DNMT1 (0.868) | BP: negative regulation of transcription from RNA polymerase II promoter BP: axon guidance BP: Wnt receptor signaling pathway, calcium modulating pathway MF: sequence-specific DNA binding MF: transcription factor activity |
| 60 | 96 |  |  | 0.774 | NHLH1 (0.774) VDR (0.774) ZBTB7A (0.774) KLF6 (0.774) PLAGL1 (0.774) RARG (0.774) PRDM4 (0.822) ZNF350 (0.822) THRB (0.822) NR2C1 (0.822) PLAG1 (0.822) RXRB (0.822) GLIS1 (0.822) SP1 (0.822) SIX2 (0.822) RARB (0.822) GLIS3 (0.822) RARA (0.822) RXRA (0.822) ZFX (0.822) NFE2L2 (0.822) MAFA (0.822) ZIC5 (0.822) CTCFL (0.822) ZIC3 (0.822) THRA (0.822) ATF1 (0.822) | BP: muscle contraction BP: negative regulation of interleukin-6 biosynthetic process CC: integral to plasma membrane CC: extracellular space MF: potassium ion binding |
| 61 | 117 |  |  | 0.775 | TFAP2B (0.775) SOHLH2 (0.775) | BP: axon guidance CC: nucleolus CC: transcription factor complex MF: transcription activator activity MF: magnesium ion binding |
| 62 | 116 |  |  | 0.803 | UBP1 (0.803) | BP: sensory perception of smell BP: G-protein coupled receptor protein signaling pathway BP: defense response to bacterium CC: extracellular region MF: olfactory receptor activity |
| 63 | 65 |  |  | 0.834 | MYCN (0.834) HINFP (0.834) BHLHE41 (0.834) KLF3 (0.834) EPAS1 (0.834) NRF1 (0.834) GLI2 (0.834) KLF12 (0.834) MYC (0.834) GLIS2 (0.834) ZIC3 (0.834) PAX1 (0.834) ENO1 (0.834) GLIS3 (0.834) ARNT (0.834) PAX9 (0.834) GLI1 (0.834) GLI3 (0.834) KLF7 (0.834) | BP: potassium ion transport BP: axon guidance MF: transcription factor activity MF: sequence-specific DNA binding MF: potassium ion binding |
| 64 | 67 |  |  | 0.836 | NRF1 (0.836) EGR1 (0.836) MBD2 (0.874) ZNF148 (0.874) DNMT1 (0.874) SP4 (0.874) ZNF281 (0.874) ATF6 (0.874) SP3 (0.874) MECP2 (0.874) TFAP2B (0.874) EGR4 (0.874) SP1 (0.874) HESX1 (0.893) | BP: negative regulation of signal transduction BP: anterior/posterior pattern formation CC: transcription factor complex CC: cytosol MF: ATP binding |
| 65 | 57 |  |  | 0.851 | TP73 (0.851) | BP: digestion BP: ion transport CC: extracellular space MF: serine-type endopeptidase activity MF: calcium ion binding |
| 66 | 83 |  |  | 0.885 | HMGA1 (0.885) BCL6 (0.885) | BP: sensory perception of smell BP: G-protein coupled receptor protein signaling pathway BP: inflammatory response CC: chromosome, centromeric region MF: olfactory receptor activity |
| 67 | 18 |  |  | 0.886 | ZIC4 (0.886) | BP: humoral immune response BP: cell-cell signaling CC: integral to plasma membrane MF: calcium ion binding MF: transcription factor activity |
| 68 | 0 |  |  | NA |  | BP: defense response to bacterium BP: calcium-mediated signaling CC: keratin filament CC: extracellular space CC: cell surface |
| 69 | 6 |  |  | NA |  | BP: rRNA processing CC: nuclear speck MF: ATP binding MF: structural constituent of ribosome MF: transcription factor activity |
| 70 | 8 |  |  | NA |  | BP: mitochondrion organization BP: branching morphogenesis of a tube BP: response to hypoxia MF: protein binding MF: potassium ion binding |
| 71 | 9 |  |  | NA |  | BP: positive regulation of glucose import BP: negative regulation of signal transduction MF: transcription factor activity MF: sequence-specific DNA binding MF: RNA polymerase II transcription factor activity |
| 72 | 13 |  |  | NA |  | BP: sensory perception of smell BP: signal transduction CC: integral to plasma membrane MF: olfactory receptor activity MF: calcium ion binding |
| 73 | 15 |  |  | NA |  | BP: regulation of transforming growth factor beta receptor signaling pathway BP: negative regulation of signal transduction BP: skeletal system morphogenesis CC: endoplasmic reticulum MF: ATP binding |
| 74 | 17 |  |  | NA |  | BP: sensory perception of smell BP: G-protein coupled receptor protein signaling pathway BP: immune response CC: integral to membrane MF: olfactory receptor activity |
| 75 | 19 |  |  | NA |  | BP: activation of protein kinase A activity BP: potassium ion transport CC: endoplasmic reticulum lumen MF: potassium ion binding MF: potassium channel activity |
| 76 | 20 |  |  | NA |  | BP: negative regulation of signal transduction CC: transcription factor complex CC: nucleolus MF: transcription activator activity MF: zinc ion binding |
| 77 | 21 |  |  | NA |  | BP: sensory perception of smell BP: regulation of intracellular pH BP: oxidative phosphorylation MF: olfactory receptor activity MF: glycosphingolipid binding |
| 78 | 22 |  |  | NA |  | CC: nucleolus CC: spliceosomal complex MF: transcription factor activity MF: RNA binding MF: sequence-specific DNA binding |
| 79 | 23 |  |  | NA |  | BP: leukotriene biosynthetic process BP: positive regulation of establishment of protein localization in plasma membrane CC: extracellular region CC: integral to membrane CC: plasma membrane part |
| 80 | 24 |  |  | NA |  | BP: sensory perception of smell BP: G-protein coupled receptor protein signaling pathway BP: defense response to bacterium CC: integral to membrane MF: olfactory receptor activity |
| 81 | 27 |  |  | NA |  | BP: epidermis development CC: mitochondrion CC: stress fiber MF: sequence-specific DNA binding MF: transcription factor activity |
| 82 | 31 |  |  | NA |  | BP: neuron fate commitment BP: negative regulation of signal transduction MF: ATP binding MF: magnesium ion binding MF: zinc ion binding |
| 83 | 32 |  |  | NA |  | BP: sensory perception of smell BP: G-protein coupled receptor protein signaling pathway BP: inflammatory response MF: olfactory receptor activity MF: taste receptor activity |
| 84 | 33 |  |  | NA |  | BP: negative regulation of signal transduction BP: axon guidance CC: transcription factor complex MF: ATP binding MF: zinc ion binding |
| 85 | 34 |  |  | NA |  | BP: Wnt receptor signaling pathway CC: nucleolus CC: cytosol MF: RNA binding MF: zinc ion binding |
| 86 | 36 |  |  | NA |  |  |
| 87 | 37 |  |  | NA |  | BP: muscle contraction BP: hexose metabolic process CC: proteinaceous extracellular matrix CC: lysosome MF: metal ion transmembrane transporter activity |
| 88 | 38 |  |  | NA |  |  |
| 89 | 39 |  |  | NA |  | BP: G-protein coupled receptor protein signaling pathway BP: sensory perception of smell CC: extracellular space CC: integral to membrane MF: olfactory receptor activity |
| 90 | 41 |  |  | NA |  | CC: extracellular region |
| 91 | 45 |  |  | NA |  | BP: cell division CC: nucleolus CC: transcription factor complex CC: cytosol MF: ATP binding |
| 92 | 47 |  |  | NA |  | BP: G-protein coupled receptor protein signaling pathway BP: defense response to bacterium BP: immune response CC: integral to plasma membrane MF: solute:sodium symporter activity |
| 93 | 48 |  |  | NA |  | BP: nuclear mRNA splicing, via spliceosome BP: anterior/posterior pattern formation CC: nucleolus CC: chromatin MF: transcription factor activity |
| 94 | 51 |  |  | NA |  | BP: immune response BP: T cell proliferation CC: extracellular space MF: peptide receptor activity, G-protein coupled |
| 95 | 52 |  |  | NA |  | BP: immune response BP: sensory perception of smell CC: integral to membrane MF: olfactory receptor activity MF: guanylate cyclase activator activity |
| 96 | 53 |  |  | NA |  | BP: negative regulation of transcription from RNA polymerase II promoter BP: potassium ion transport MF: transcription factor activity MF: sequence-specific DNA binding MF: potassium ion binding |
| 97 | 54 |  |  | NA |  | BP: sensory perception of smell BP: G-protein coupled receptor protein signaling pathway BP: defense response to bacterium CC: extracellular space MF: olfactory receptor activity |
| 98 | 55 |  |  | NA |  | BP: neuron fate specification BP: negative regulation of signal transduction CC: transcription factor complex CC: nucleolus MF: zinc ion binding |
| 99 | 56 |  |  | NA |  | BP: muscle contraction BP: regulation of membrane potential CC: sarcomere MF: serine-type endopeptidase activity MF: cation channel activity |
| 100 | 60 |  |  | NA |  | CC: nucleolus CC: cytosol MF: transcription activator activity MF: zinc ion binding MF: ATP binding |
| 101 | 61 |  |  | NA |  | BP: regulation of lipid transport CC: extracellular region CC: endoplasmic reticulum |
| 102 | 62 |  |  | NA |  | CC: nucleolus CC: cytosol MF: transcription activator activity MF: RNA binding MF: ATP binding |
| 103 | 63 |  |  | NA |  | BP: sensory perception of smell BP: G-protein coupled receptor protein signaling pathway BP: inflammatory response BP: immune response MF: olfactory receptor activity |
| 104 | 66 |  |  | NA |  | BP: cellular carbohydrate catabolic process MF: structural molecule activity |
| 105 | 68 |  |  | NA |  | BP: RNA splicing BP: negative regulation of translational initiation in response to stress BP: cellular biosynthetic process CC: cytosolic part CC: nuclear part |
| 106 | 69 |  |  | NA |  | CC: extracellular space CC: integral to plasma membrane MF: calcium ion binding MF: serine-type endopeptidase activity MF: cytokine receptor activity |
| 107 | 71 |  |  | NA |  | CC: nucleolus CC: transcription factor complex CC: spliceosomal complex MF: ATP binding MF: protein serine/threonine kinase activity |
| 108 | 72 |  |  | NA |  | BP: Wnt receptor signaling pathway, calcium modulating pathway BP: cell development BP: positive regulation of glucose import MF: transcription factor activity MF: sequence-specific DNA binding |
| 109 | 73 |  |  | NA |  | BP: nuclear mRNA splicing, via spliceosome CC: nucleolus MF: ATP binding MF: zinc ion binding MF: structural constituent of ribosome |
| 110 | 79 |  |  | NA |  | BP: small GTPase mediated signal transduction CC: nucleolus CC: spliceosomal complex MF: RNA binding MF: ATP binding |
| 111 | 80 |  |  | NA |  | BP: axon guidance BP: small GTPase mediated signal transduction CC: transcription factor complex MF: transcription activator activity MF: transcription corepressor activity |
| 112 | 81 |  |  | NA |  | BP: sensory perception of smell BP: G-protein coupled receptor protein signaling pathway BP: immune response CC: extracellular region MF: olfactory receptor activity |
| 113 | 91 |  |  | NA |  | CC: cytosol CC: nucleolus MF: zinc ion binding MF: ATP binding MF: GTPase activity |
| 114 | 92 |  |  | NA |  | BP: muscle contraction CC: extracellular region CC: keratin filament CC: voltage-gated calcium channel complex CC: integral to plasma membrane |
| 115 | 98 |  |  | NA |  | BP: anterior/posterior pattern formation CC: nucleolus CC: transcription factor complex MF: ATP binding MF: translation regulator activity |
| 116 | 100 |  |  | NA |  | BP: nuclear mRNA splicing, via spliceosome CC: nucleolus MF: sequence-specific DNA binding MF: transcription factor activity MF: RNA polymerase II transcription factor activity |
| 117 | 103 |  |  | NA |  | BP: anterior/posterior pattern formation CC: nucleolus CC: cytosol MF: transcription factor activity MF: ATP binding |
| 118 | 104 |  |  | NA |  |  |
| 119 | 107 |  |  | NA |  | BP: negative regulation of transcription from RNA polymerase II promoter BP: negative regulation of signal transduction BP: inner ear morphogenesis CC: transcription factor complex MF: transcription activator activity |
| 120 | 109 |  |  | NA |  | BP: sensory perception of smell BP: G-protein coupled receptor protein signaling pathway CC: spliceosomal complex MF: olfactory receptor activity MF: RNA binding |
| 121 | 110 |  |  | NA |  | BP: sensory perception of smell BP: G-protein coupled receptor protein signaling pathway MF: olfactory receptor activity |
| 122 | 111 |  |  | NA |  | BP: negative regulation of signal transduction CC: transcription factor complex CC: cytosol MF: transcription activator activity MF: ATP binding |
| 123 | 112 |  |  | NA |  | BP: positive regulation of transcription from RNA polymerase II promoter BP: protein amino acid phosphorylation BP: embryonic limb morphogenesis CC: transcription factor complex CC: Golgi membrane |
| 124 | 113 |  |  | NA |  | CC: transcription factor complex CC: cytosol MF: zinc ion binding MF: GTPase activity MF: ATP binding |
| 125 | 114 |  |  | NA |  | CC: nucleolus CC: cytosol MF: RNA binding MF: ATP binding MF: zinc ion binding |
| 126 | 120 |  |  | NA |  | BP: Wnt receptor signaling pathway, calcium modulating pathway BP: Ras protein signal transduction CC: cytoplasm MF: transcription factor activity MF: sequence-specific DNA binding |
| 127 | 123 |  |  | NA |  | BP: sensory perception of smell BP: G-protein coupled receptor protein signaling pathway CC: plasma membrane CC: integral to membrane MF: olfactory receptor activity |
| 128 | 127 |  |  | NA |  | BP: nuclear mRNA splicing, via spliceosome BP: DNA repair CC: nucleolus CC: spliceosomal complex MF: ATP binding |
