# Supplementary material for: DeepCpG: accurate prediction of single-cell DNA methylation states using deep learning
Source: Genome Biol. 2017 Apr 11;18:67. doi: 10.1186/s13059-017-1189-z (PMC5387360; doi:10.1186/s13059-017-1189-z)
Supplement: Supplementary file 3 — Sequence motifs. HTML files with sequence logos and summary statistics for all cell types. (ZIP 19482 kb) [file 13059_2017_1189_MOESM3_ESM.zip › Hep.html]

Filter table


# Filter table

#### *Christof Angermueller*

#### *2016-12-17*

Nr | Label | Logo | Influence | q-value | Related known motifs | GO annotations || 1 | 78: NR3C2 |  |  | 0.004 | NR3C2 (0.004) ATOH8 (0.009) FIGLA (0.022) SNAI1 (0.039) SNAI2 (0.101) SCRT1 (0.124) MESP1 (0.134) TWIST1 (0.139) MXI1 (0.152) MYCL1 (0.254) MYF6 (0.296) KLF8 (0.297) MNT (0.304) MSC (0.304) TGIF1 (0.333) ATOH1 (0.333) SCRT2 (0.358) TBX4 (0.358) CLOCK (0.358) ZNF350 (0.358) MYCN (0.358) KLF1 (0.375) MYBL2 (0.392) TBX5 (0.397) TBX18 (0.412) | BP: immune response CC: lysosome MF: cofactor binding MF: serine-type endopeptidase activity MF: hormone activity |
| 2 | 55: NAIF1 |  |  | 0.011 | NAIF1 (0.011) HLF (0.143) HIVEP2 (0.186) MYPOP (0.396) GMEB1 (0.396) GMEB2 (0.396) HIC1 (0.396) ATF4 (0.422) CEBPE (0.795) EOMES (0.795) ZHX1 (0.795) CREB1 (0.795) CEBPD (0.795) ATF2 (0.795) SREBF1 (0.795) CEBPA (0.795) DBP (0.795) CEBPB (0.795) TCFL5 (0.795) ATF1 (0.795) AHR (0.795) HES7 (0.795) OTX1 (0.795) ZFY (0.795) HES5 (0.795) EGR2 (0.795) | BP: nuclear mRNA splicing, via spliceosome CC: spliceosomal complex CC: nucleolus CC: mitochondrial inner membrane MF: structural constituent of ribosome |
| 3 | 4: GLIS2 |  |  | 0.014 | GLIS2 (0.014) ZIC5 (0.045) PLAGL1 (0.045) ZIC4 (0.045) GLIS3 (0.068) GLIS1 (0.090) NRF1 (0.090) E2F1 (0.201) ZIC3 (0.297) SOHLH2 (0.406) ZFP161 (0.554) GLI2 (0.637) ZBTB7B (0.664) SP4 (0.664) TFAP2B (0.664) EGR2 (0.664) KLF8 (0.664) EGR1 (0.769) ZNF219 (0.784) ZBTB7A (0.842) PLAG1 (0.857) | CC: nucleolus CC: cytosol MF: zinc ion binding MF: RNA binding MF: ATP binding |
| 4 | 86: ZNF219 |  |  | 0.017 | ZNF219 (0.017) SP2 (0.017) SP3 (0.017) KLF6 (0.034) ZBTB7B (0.037) ZNF263 (0.051) SP4 (0.051) MAZ (0.052) WT1 (0.056) ZNF148 (0.085) ZBTB7A (0.089) ZNF281 (0.089) EOMES (0.129) SP1 (0.138) EGR2 (0.138) EGR1 (0.154) ZNF740 (0.154) PURA (0.154) ETV6 (0.162) KLF15 (0.208) MLL (0.208) MZF1 (0.349) E2F6 (0.451) MBD2 (0.459) PLAGL1 (0.464) ZBTB49 (0.464) E2F1 (0.464) NRF1 (0.464) SP5 (0.464) ELK4 (0.464) ZNF350 (0.464) | BP: small GTPase mediated signal transduction BP: Wnt receptor signaling pathway CC: nucleolus CC: transcription factor complex MF: ATP binding |
| 5 | 43: ETV6 |  |  | 0.023 | ETV6 (0.023) ELK4 (0.037) MECP2 (0.172) TFAP2D (0.172) ZIC4 (0.339) KLF6 (0.339) EGR1 (0.339) GABPA (0.339) TFAP2E (0.339) GLIS2 (0.339) FLI1 (0.339) ERF (0.349) ETV3 (0.349) ETS2 (0.349) SP4 (0.407) WT1 (0.407) TFAP2A (0.407) ZBTB7A (0.407) SP2 (0.407) ZFY (0.407) TFAP2B (0.407) ZBTB7B (0.408) USF2 (0.419) MBD2 (0.485) EGR2 (0.485) ELF4 (0.485) CTCFL (0.485) MLL (0.485) | CC: nucleolus CC: transcription factor complex CC: cytosol MF: ATP binding MF: zinc ion binding |
| 6 | 74: HMG20B |  |  | 0.027 | HMG20B (0.027) ARID3B (0.084) ARID3A (0.328) DMRT2 (0.328) ZFHX3 (0.425) ARX (0.445) PHOX2A (0.445) LHX3 (0.445) UNCX (0.445) CDC5L (0.460) FOXQ1 (0.460) DRGX (0.460) NKX3-1 (0.460) PHOX2B (0.460) PROP1 (0.477) HOXD9 (0.477) POU4F3 (0.489) FOXF1 (0.489) ALX4 (0.548) DUX4 (0.548) POU4F1 (0.548) CPEB1 (0.548) DMRTA2 (0.548) POU3F2 (0.548) LHX1 (0.560) LHX5 (0.560) ISX (0.560) | BP: sensory perception of smell BP: G-protein coupled receptor protein signaling pathway BP: DNA damage checkpoint CC: proteasome complex MF: olfactory receptor activity |
| 7 | 27: TFAP2B |  |  | 0.029 | TFAP2B (0.029) ZFY (0.289) ZFX (0.363) TFAP2C (0.770) | CC: transcription factor complex CC: stress fiber MF: transcription factor activity MF: sequence-specific DNA binding MF: ATP binding |
| 8 | 35: TFAP2D |  |  | 0.044 | TFAP2D (0.044) WT1 (0.114) ZFX (0.114) ZFY (0.114) TWIST1 (0.128) PAX5 (0.323) EGR1 (0.323) TFAP4 (0.327) NRF1 (0.348) SP3 (0.348) SNAI1 (0.348) DNMT1 (0.348) SP2 (0.459) MESP1 (0.487) PAX8 (0.487) TP73 (0.487) TFAP2E (0.487) ARNT2 (0.487) KLF6 (0.487) ZIC4 (0.487) KLF3 (0.487) SP1 (0.487) TFAP2B (0.487) CTCFL (0.530) SP4 (0.553) | BP: positive regulation of transcription from RNA polymerase II promoter CC: transcription factor complex CC: cytosol MF: transcription activator activity MF: ATP binding |
| 9 | 95: ZFX |  |  | 0.045 | ZFX (0.045) TFAP2D (0.252) PLAG1 (0.252) PLAGL1 (0.252) CTCF (0.359) EBF1 (0.359) ZNF350 (0.392) TFAP2B (0.392) HINFP (0.485) KLF6 (0.565) TWIST1 (0.565) ARNT2 (0.600) TFAP2C (0.627) ZFY (0.643) CTCFL (0.656) EGR1 (0.672) PPARD (0.672) SP3 (0.672) ZNF148 (0.672) SMAD1 (0.672) MBD2 (0.672) ZIC5 (0.672) GLIS1 (0.672) ESR1 (0.672) SNAI1 (0.672) USF2 (0.672) NFE2L2 (0.672) SP4 (0.672) KLF16 (0.672) EGR4 (0.672) | BP: cell development BP: regulation of transforming growth factor beta receptor signaling pathway MF: transcription factor activity MF: sequence-specific DNA binding MF: identical protein binding |
| 10 | 119: ZFP161 |  |  | 0.048 | ZFP161 (0.048) E2F1 (0.377) HIF1A (0.404) TFAP2D (0.723) NRF1 (0.723) HES5 (0.737) HES7 (0.826) DNMT1 (0.839) ZBTB7C (0.839) | CC: transcription factor complex CC: nucleolus CC: cytosol MF: ATP binding MF: zinc ion binding |
| 11 | 99 |  |  | 0.053 | OSR2 (0.053) OSR1 (0.074) INSM1 (0.229) TCF4 (0.229) ZNF691 (0.229) ETV1 (0.437) SIX6 (0.475) ZNF652 (0.489) MESP1 (0.759) VDR (0.759) | BP: muscle contraction BP: pyruvate metabolic process BP: myofibril assembly CC: integral to plasma membrane CC: extracellular space |
| 12 | 89 |  |  | 0.055 | ZBTB7B (0.055) DNMT1 (0.193) E2F1 (0.193) SP3 (0.201) SP4 (0.211) WT1 (0.331) TFAP2D (0.331) EGR1 (0.331) MAZ (0.367) SP1 (0.367) HES1 (0.367) ZFP161 (0.367) E2F6 (0.367) SP2 (0.367) PLAGL1 (0.367) KLF5 (0.367) ZFX (0.458) NHLH1 (0.478) ZBTB7C (0.478) EGR2 (0.492) PURA (0.534) ZNF263 (0.538) MBD2 (0.599) KLF4 (0.599) TFAP4 (0.601) CTCFL (0.601) CTCF (0.601) | BP: negative regulation of signal transduction CC: transcription factor complex CC: nucleolus MF: chromatin binding MF: zinc ion binding |
| 13 | 102 |  |  | 0.061 | NRF1 (0.061) | BP: nuclear mRNA splicing, via spliceosome BP: cell division BP: small GTPase mediated signal transduction MF: ATP binding MF: zinc ion binding |
| 14 | 17 |  |  | 0.071 | DMRTC2 (0.071) FOXO6 (0.071) | BP: sensory perception of smell BP: G-protein coupled receptor protein signaling pathway BP: inflammatory response MF: olfactory receptor activity MF: taste receptor activity |
| 15 | 126 |  |  | 0.072 | NRF1 (0.072) TFAP2D (0.094) ZFX (0.318) ZFY (0.318) TFAP2E (0.318) E2F1 (0.318) TFAP2B (0.408) EGR2 (0.408) CTCFL (0.408) NHLH1 (0.425) MBD2 (0.425) DNMT1 (0.425) MYC (0.425) HEY2 (0.425) ZIC4 (0.425) ENO1 (0.425) WT1 (0.425) GLIS2 (0.425) MESP1 (0.425) MYCL1 (0.425) EGR1 (0.425) ZBTB7C (0.425) TCF4 (0.425) SP4 (0.433) MYCN (0.433) EGR4 (0.433) | CC: transcription factor complex CC: nucleolus CC: cytosol MF: RNA binding MF: ATP binding |
| 16 | 117 |  |  | 0.073 | TFAP2B (0.073) TFAP2E (0.774) CTCFL (0.786) ZNF423 (0.786) | BP: anterior/posterior pattern formation BP: negative regulation of signal transduction CC: transcription factor complex MF: RNA binding MF: zinc ion binding |
| 17 | 50 |  |  | 0.074 | ZFY (0.074) SP4 (0.128) SP3 (0.128) KLF12 (0.128) KLF4 (0.128) KLF7 (0.128) KLF2 (0.128) KLF14 (0.128) ZFX (0.141) KLF5 (0.150) PLAGL1 (0.172) KLF16 (0.201) MTF1 (0.201) SP5 (0.201) EGR4 (0.201) SP8 (0.256) SP1 (0.256) E2F1 (0.256) ZIC5 (0.256) MBD2 (0.332) EGR1 (0.365) KLF6 (0.411) SP2 (0.419) TFAP2D (0.419) ZBTB49 (0.419) | BP: negative regulation of signal transduction CC: cytosol CC: transcription factor complex MF: ATP binding MF: GTPase activity |
| 18 | 15 |  |  | 0.079 | ZFP161 (0.079) SP1 (0.339) NRF1 (0.339) EGR1 (0.339) WT1 (0.339) SP2 (0.339) E2F1 (0.339) ZBTB7B (0.339) SP3 (0.339) GLIS2 (0.339) PLAGL1 (0.339) ZIC5 (0.339) SP4 (0.339) KLF6 (0.339) ZIC4 (0.354) EGR4 (0.424) HIF1A (0.455) E2F6 (0.493) EGR2 (0.564) GLIS3 (0.564) KLF16 (0.564) TCF4 (0.564) GCM1 (0.564) ZFX (0.564) SP5 (0.564) | CC: nucleolus CC: transcription factor complex CC: cytosol MF: zinc ion binding MF: ATP binding |
| 19 | 108 |  |  | 0.087 | ETS1 (0.087) SMARCC2 (0.087) GLIS1 (0.194) EBF1 (0.644) STAT4 (0.644) PLAG1 (0.644) ZNF76 (0.644) TWIST1 (0.644) BCL6B (0.724) BCL6 (0.724) SNAI1 (0.724) MECP2 (0.746) STAT6 (0.772) RBPJ (0.832) | BP: immune response CC: integral to plasma membrane CC: extracellular space MF: peptide receptor activity, G-protein coupled MF: receptor binding |
| 20 | 9 |  |  | 0.100 | E2F1 (0.100) ZFY (0.100) SP4 (0.100) ZFP161 (0.125) TFAP2D (0.154) SP3 (0.154) KLF14 (0.188) CTCFL (0.189) SP1 (0.189) KLF7 (0.189) KLF16 (0.189) DNMT1 (0.189) KLF12 (0.189) KLF5 (0.189) TCFL5 (0.189) KLF4 (0.189) KLF6 (0.189) ZFX (0.189) ZKSCAN3 (0.189) KLF2 (0.189) SP2 (0.189) HES1 (0.219) CTCF (0.229) ZBTB7A (0.229) KLF13 (0.229) | CC: nucleolus CC: transcription factor complex CC: cytosol MF: ATP binding MF: zinc ion binding |
| 21 | 36 |  |  | 0.105 | ESR1 (0.105) ESR2 (0.105) FOSL2 (0.121) | BP: transmembrane transport BP: cellular ion homeostasis BP: metal ion transport CC: myosin complex MF: serine-type endopeptidase activity |
| 22 | 40 |  |  | 0.108 | ZFY (0.108) KLF1 (0.820) ZFX (0.820) WT1 (0.820) EGR4 (0.820) MBD2 (0.820) KLF5 (0.820) TFAP4 (0.820) ZNF281 (0.820) KLF16 (0.820) YBX1 (0.820) SP1 (0.820) KLF6 (0.820) ZNF148 (0.820) EGR1 (0.820) NRF1 (0.820) TFAP2D (0.820) ELK4 (0.820) NHLH1 (0.820) ATF6 (0.820) KLF2 (0.820) KLF4 (0.820) SP2 (0.820) GSC2 (0.820) ZIC1 (0.820) DNMT1 (0.820) SP3 (0.820) ZBTB7B (0.820) ZNF219 (0.820) ZBTB7A (0.820) SP4 (0.820) | CC: nucleolus CC: transcription factor complex MF: ATP binding MF: zinc ion binding MF: GTPase activity |
| 23 | 97 |  |  | 0.109 | TBX20 (0.109) MYF6 (0.174) MGA (0.316) TBX18 (0.316) TBX4 (0.316) TBX5 (0.319) NHLH2 (0.526) MESP1 (0.526) SNAI1 (0.526) TCF4 (0.809) TBR1 (0.809) | BP: sensory perception of smell BP: immune response BP: G-protein coupled receptor protein signaling pathway CC: extracellular space MF: olfactory receptor activity |
| 24 | 118 |  |  | 0.111 | SP3 (0.111) CTCFL (0.111) WT1 (0.111) TFAP2B (0.111) MECP2 (0.189) ZFX (0.189) EGR1 (0.189) DNMT1 (0.199) ZFY (0.214) TFAP2D (0.234) CTCF (0.416) SP4 (0.416) KLF6 (0.416) MBD2 (0.473) EGR2 (0.555) ZBTB7A (0.581) E2F1 (0.625) ZBTB7B (0.625) PLAG1 (0.625) EGR4 (0.625) HSF1 (0.625) ARNT2 (0.625) PLAGL1 (0.625) ZIC5 (0.643) ZBTB49 (0.661) SP1 (0.661) GLIS2 (0.661) SP2 (0.661) | CC: cytosol CC: nucleolus MF: ATP binding MF: RNA binding MF: zinc ion binding |
| 25 | 84 |  |  | 0.121 | CGBP (0.121) | BP: negative regulation of signal transduction CC: nucleolus CC: transcription factor complex MF: ATP binding MF: zinc ion binding |
| 26 | 105 |  |  | 0.135 | SP1 (0.135) SP5 (0.135) KLF16 (0.147) SP3 (0.147) KLF5 (0.147) KLF7 (0.147) KLF12 (0.147) SP2 (0.147) KLF4 (0.161) ZBTB7B (0.161) WT1 (0.161) KLF2 (0.161) SP4 (0.161) SP8 (0.161) EGR1 (0.270) NHLH1 (0.273) ZNF219 (0.290) EGR4 (0.290) ZNF148 (0.346) RREB1 (0.346) KLF13 (0.364) MAZ (0.364) KLF14 (0.432) KLF6 (0.437) GMEB1 (0.437) | CC: cytosol CC: nucleolus CC: transcription factor complex MF: RNA binding MF: ATP binding |
| 27 | 91 |  |  | 0.139 | WT1 (0.139) E2F1 (0.139) SP3 (0.139) KLF6 (0.139) SP4 (0.139) TFAP2D (0.139) ELK4 (0.139) DNMT1 (0.139) EGR1 (0.139) SP1 (0.143) ZFY (0.143) ZFP161 (0.143) ZFX (0.156) SP2 (0.178) ETV6 (0.192) CTCFL (0.192) ZBTB7B (0.206) PLAGL1 (0.210) TFAP2B (0.269) MBD2 (0.366) ZBTB7A (0.375) HIF1A (0.375) EGR2 (0.376) USF2 (0.395) KLF16 (0.429) | CC: transcription factor complex CC: cytosol CC: nucleolus MF: ATP binding MF: zinc ion binding |
| 28 | 83 |  |  | 0.153 | MAX (0.153) MYC (0.153) SNAI1 (0.820) MYCN (0.896) ZIC5 (0.896) | CC: integral to plasma membrane CC: proteinaceous extracellular matrix CC: extracellular space MF: potassium ion binding MF: extracellular matrix structural constituent |
| 29 | 12 |  |  | 0.155 | EBF1 (0.155) ZFX (0.155) CTCFL (0.155) CTCF (0.155) TFAP2A (0.155) TFAP2D (0.155) TFAP2B (0.176) EGR4 (0.180) TFAP2C (0.200) ZNF148 (0.350) ARNT2 (0.363) DNMT1 (0.363) SP4 (0.411) ESR1 (0.411) ZIC5 (0.411) EGR2 (0.411) TWIST1 (0.411) ZBTB7A (0.411) PLAGL1 (0.411) NKX2-1 (0.433) ZNF281 (0.458) SNAI1 (0.458) ZNF423 (0.458) ZBTB7B (0.535) THRB (0.566) ZFY (0.566) KLF6 (0.566) ZNF589 (0.566) SMAD1 (0.566) | BP: anterior/posterior pattern formation BP: negative regulation of signal transduction BP: heart morphogenesis CC: extrinsic to membrane CC: mitochondrion |
| 30 | 88 |  |  | 0.172 | KLF8 (0.172) ZIC5 (0.360) FLI1 (0.360) ETV4 (0.360) SCRT2 (0.360) ATOH8 (0.360) KLF1 (0.360) TCF12 (0.360) ENO1 (0.360) SP2 (0.360) TCF4 (0.360) PLAGL1 (0.360) ETS2 (0.360) ELK3 (0.360) SP4 (0.360) ELK4 (0.360) ZBTB7A (0.375) KLF15 (0.375) FIGLA (0.408) ELF2 (0.425) EHF (0.425) NR2C1 (0.457) ZIC4 (0.457) ELF1 (0.457) ZNF219 (0.457) ATOH1 (0.457) EGR1 (0.457) MYF6 (0.457) SNAI1 (0.457) ZSCAN4 (0.457) MYC (0.457) ZBTB7B (0.457) NEUROD1 (0.457) RARG (0.457) RARA (0.457) ZSCAN10 (0.457) TWIST1 (0.457) E2F1 (0.457) SPI1 (0.457) ZNF238 (0.457) MYBL2 (0.457) MYOD1 (0.457) SCRT1 (0.457) SMARCC2 (0.457) NR3C1 (0.457) GLIS2 (0.457) SNAI2 (0.457) RORC (0.457) SP1 (0.457) RARB (0.457) | BP: regulation of transcription from RNA polymerase II promoter CC: nucleus CC: mitochondrion MF: insulin-like growth factor binding MF: magnesium ion binding |
| 31 | 85 |  |  | 0.190 | MBD2 (0.190) CTCFL (0.190) ZFX (0.190) WT1 (0.261) ZFY (0.261) PLAGL1 (0.261) TFAP2D (0.261) DNMT1 (0.261) EGR1 (0.261) SP4 (0.286) TFAP2B (0.313) CTCF (0.322) EGR4 (0.322) KLF6 (0.324) E2F1 (0.345) NRF1 (0.347) ZBTB7C (0.347) SP3 (0.347) ZBTB7A (0.347) USF2 (0.352) ZIC5 (0.372) ZBTB7B (0.372) HES1 (0.440) ELK4 (0.472) ARNT2 (0.474) | CC: transcription factor complex CC: nucleolus CC: cytosol MF: zinc ion binding MF: ATP binding |
| 32 | 33 |  |  | 0.195 | TCFL5 (0.195) SP4 (0.195) E2F1 (0.195) HES5 (0.195) HES7 (0.236) SP1 (0.306) ZFP161 (0.306) SP3 (0.306) HES1 (0.306) SP2 (0.306) ZIC4 (0.306) HEY2 (0.306) E2F2 (0.306) ZBTB1 (0.315) KLF5 (0.315) HESX1 (0.315) CTCFL (0.315) KLF7 (0.315) HEY1 (0.315) ZFX (0.315) KLF16 (0.347) ARNT (0.347) EGR1 (0.347) KLF4 (0.347) ZBTB7B (0.347) AHR (0.347) | BP: negative regulation of signal transduction CC: nucleolus CC: transcription factor complex MF: ATP binding MF: zinc ion binding |
| 33 | 48 |  |  | 0.196 | USF2 (0.196) ETV6 (0.774) ELK4 (0.774) ATF6 (0.774) MBD2 (0.774) CREB3L1 (0.774) TFAP2D (0.774) TCFL5 (0.774) ARNT (0.774) FLI1 (0.881) MYCN (0.881) SP3 (0.882) | CC: nucleolus CC: transcription factor complex CC: cytosol MF: RNA binding MF: ATP binding |
| 34 | 18 |  |  | 0.209 | MECP2 (0.209) TFAP2B (0.376) TFAP2D (0.376) EBF1 (0.376) GLIS2 (0.382) ZIC5 (0.382) TFAP2C (0.421) TFAP2A (0.521) SMARCC2 (0.608) CTCFL (0.772) INSM1 (0.772) TFAP2E (0.772) ZIC4 (0.772) BRCA1 (0.772) ZBTB33 (0.772) DNMT1 (0.772) ETS1 (0.772) PLAGL1 (0.772) MBD2 (0.772) GLIS1 (0.781) SP4 (0.781) GLIS3 (0.795) GABPA (0.821) | BP: negative regulation of signal transduction BP: negative regulation of cell migration CC: transcription factor complex CC: cytosol MF: zinc ion binding |
| 35 | 31 |  |  | 0.209 | SP3 (0.209) ZFX (0.209) WT1 (0.209) TFAP2D (0.209) ZFY (0.209) EGR1 (0.391) EGR4 (0.391) SP4 (0.391) USF2 (0.417) SP1 (0.417) MBD2 (0.417) KLF16 (0.417) TFAP2B (0.417) E2F1 (0.472) KLF6 (0.472) PLAGL1 (0.504) ETV6 (0.566) ZBTB7A (0.598) CTCFL (0.619) EGR2 (0.619) SP2 (0.619) ZIC5 (0.619) KLF5 (0.619) DNMT1 (0.619) TFAP4 (0.625) | CC: transcription factor complex CC: cytosol CC: nucleolus MF: ATP binding MF: zinc ion binding |
| 36 | 45 |  |  | 0.219 | MITF (0.219) BHLHE40 (0.230) BACH1 (0.230) ATF3 (0.230) THRB (0.230) TFEC (0.230) MBD2 (0.230) SREBF1 (0.230) SNAI2 (0.230) TCFL5 (0.348) MLX (0.423) HES2 (0.423) TFAP2E (0.430) USF2 (0.434) USF1 (0.434) TFEB (0.434) ARNT (0.441) NR1I3 (0.485) ZEB1 (0.516) MAFB (0.542) THRA (0.542) AHR (0.542) E4F1 (0.542) SREBF2 (0.574) TFAP2D (0.574) | BP: anterior/posterior pattern formation CC: cytosol CC: nucleolus MF: RNA binding MF: ATP binding |
| 37 | 60 |  |  | 0.247 | HES1 (0.247) TCFL5 (0.247) BRCA1 (0.247) ZBTB33 (0.293) BHLHE40 (0.617) E2F1 (0.760) MXI1 (0.760) ZFP161 (0.760) ZNF524 (0.884) NRF1 (0.884) EPAS1 (0.884) TBX20 (0.884) E2F6 (0.884) | CC: nucleolus CC: transcription factor complex CC: spliceosomal complex MF: zinc ion binding MF: ATP binding |
| 38 | 32 |  |  | 0.253 | PHOX2A (0.253) PHOX2B (0.253) ARID3B (0.253) ARX (0.253) LHX3 (0.253) DRGX (0.253) LHX1 (0.253) UNCX (0.253) LIN54 (0.253) ISX (0.253) ALX1 (0.253) LHX5 (0.253) PROP1 (0.280) ALX4 (0.293) FOXL2 (0.348) ARID3A (0.359) AC012531.1 (0.359) GSX2 (0.359) POU3F3 (0.359) HLX (0.387) MNX1 (0.396) GSX1 (0.445) LMX1A (0.445) POU1F1 (0.445) MIXL1 (0.445) ARID3C (0.445) VAX1 (0.445) PRRX2 (0.445) LHX2 (0.445) HMG20B (0.445) HOXD9 (0.445) CDX1 (0.445) VAX2 (0.445) LMX1B (0.445) VENTX (0.445) CUX2 (0.445) | BP: sensory perception of smell BP: G-protein coupled receptor protein signaling pathway BP: double-strand break repair MF: olfactory receptor activity MF: taste receptor activity |
| 39 | 71 |  |  | 0.261 | ETV6 (0.261) ELK4 (0.799) | CC: integral to membrane |
| 40 | 114 |  |  | 0.269 | ZIC4 (0.269) | BP: small GTPase mediated signal transduction BP: Wnt receptor signaling pathway, calcium modulating pathway MF: transcription factor activity MF: sequence-specific DNA binding MF: potassium ion binding |
| 41 | 121 |  |  | 0.307 | MBD2 (0.307) ZBTB49 (0.307) SP3 (0.396) ZFX (0.758) WT1 (0.758) ZFP161 (0.758) MECP2 (0.758) | CC: transcription factor complex CC: cytosol CC: nucleolus MF: ATP binding MF: magnesium ion binding |
| 42 | 69 |  |  | 0.309 | PLAGL1 (0.309) ZNF202 (0.331) HEY2 (0.331) MLXIPL (0.331) ZNF691 (0.331) HIC2 (0.331) MYF5 (0.421) NHLH1 (0.439) PLAG1 (0.494) MZF1 (0.494) MYC (0.494) GLIS3 (0.494) BHLHE40 (0.591) FOSL1 (0.591) PURA (0.591) THRB (0.591) BHLHE41 (0.609) HEY1 (0.609) MXI1 (0.609) ZNF350 (0.609) ARNTL (0.613) ZNF219 (0.613) INSM1 (0.684) KLF6 (0.684) MAX (0.733) SIM1 (0.733) TFEB (0.733) MYCL1 (0.733) HIC1 (0.733) ZFP161 (0.733) USF1 (0.733) ZIC5 (0.733) NR4A1 (0.733) ZBTB7A (0.733) KLF16 (0.733) ID2 (0.733) CTCFL (0.733) VDR (0.733) ATF3 (0.733) NR0B1 (0.733) NPAS2 (0.733) MAZ (0.733) MNT (0.733) ZNF740 (0.733) NEUROD1 (0.733) SMARCC1 (0.733) SP4 (0.733) RORB (0.733) E2F1 (0.733) HIF1A (0.733) ARNT2 (0.733) RARG (0.733) ZNF784 (0.733) NR4A2 (0.733) SP3 (0.733) USF2 (0.733) KLF14 (0.733) TFAP2D (0.733) ZIC2 (0.733) PRDM4 (0.733) CTCF (0.733) HES7 (0.733) ZBTB7B (0.733) MLXIP (0.733) EGR4 (0.733) SMAD2 (0.733) HES5 (0.733) PPARA (0.733) SP2 (0.733) ZIC4 (0.733) MYCN (0.733) CREB3L2 (0.733) TCF12 (0.733) XBP1 (0.733) FERD3L (0.733) KLF5 (0.733) GLIS2 (0.733) BACH1 (0.733) ZIC1 (0.733) SP8 (0.733) PTF1A (0.733) | BP: potassium ion transport BP: epidermis development MF: potassium ion binding MF: sequence-specific DNA binding MF: transcription factor activity |
| 43 | 56 |  |  | 0.320 | NR3C2 (0.320) GLI1 (0.320) ESR2 (0.320) ESR1 (0.320) KLF6 (0.320) ZIC5 (0.320) ZNF219 (0.403) CTCF (0.403) SNAI1 (0.403) CTCFL (0.403) GLI2 (0.403) GLIS1 (0.482) GLI3 (0.482) NFKB2 (0.482) ESRRA (0.482) RELB (0.482) MXI1 (0.619) HOXB1 (0.658) KLF8 (0.705) PLAGL1 (0.732) ZBTB7A (0.732) REL (0.732) KLF15 (0.753) ZNF354C (0.753) GLIS3 (0.753) ZIC2 (0.753) | BP: cell-cell signaling CC: integral to plasma membrane CC: extracellular space MF: serine-type endopeptidase activity MF: calcium ion binding |
| 44 | 16 |  |  | 0.327 | SMAD1 (0.327) ZBTB3 (0.327) | BP: potassium ion transport BP: regulation of transforming growth factor beta receptor signaling pathway MF: potassium ion binding MF: transcription factor activity MF: sequence-specific DNA binding |
| 45 | 116 |  |  | 0.337 | SNAI1 (0.337) TWIST1 (0.338) ETV2 (0.338) ERF (0.338) MYBL2 (0.338) ELK3 (0.338) ETV3 (0.338) FIGLA (0.338) SNAI2 (0.338) ETV7 (0.338) ELF4 (0.338) ERG (0.338) ATOH8 (0.338) ETV1 (0.343) GABPA (0.347) ELK1 (0.375) MECP2 (0.444) ZNF238 (0.459) SPDEF (0.571) EBF1 (0.571) PRKRIR (0.614) ZBTB4 (0.619) ENSG00000235187 (0.621) CREB3L2 (0.680) NFAT5 (0.680) TFAP2B (0.680) CTCF (0.680) | BP: embryonic heart tube development BP: axon guidance CC: nucleoplasm MF: transcription factor activity MF: sequence-specific DNA binding |
| 46 | 106 |  |  | 0.380 | NFIA (0.380) NFIX (0.380) | BP: small GTPase mediated signal transduction CC: nucleolus CC: cytosol MF: sequence-specific DNA binding MF: transcription factor activity |
| 47 | 92 |  |  | 0.384 | LHX8 (0.384) CEBPZ (0.384) ENSG00000229544 (0.384) VSX2 (0.384) MAFB (0.384) EN1 (0.384) CIC (0.384) NKX1-1 (0.384) GBX1 (0.384) POU3F3 (0.384) GBX2 (0.393) ESX1 (0.555) MYBL2 (0.555) NFYA (0.564) MAX (0.564) POU2F3 (0.564) ELF3 (0.564) LHX6 (0.564) POU3F4 (0.564) POU5F1B (0.564) DLX3 (0.564) NFYB (0.564) YBX1 (0.564) CEBPG (0.564) ALX3 (0.564) EVX2 (0.564) OTP (0.564) POU2F1 (0.564) HOXB3 (0.564) | BP: sensory perception of smell BP: G-protein coupled receptor protein signaling pathway CC: extracellular space CC: plasma membrane MF: olfactory receptor activity |
| 48 | 58 |  |  | 0.419 | NHLH2 (0.419) MYOD1 (0.419) MXI1 (0.419) ZFX (0.419) TCF4 (0.419) MYF5 (0.469) MYF6 (0.469) MYCL1 (0.589) MYOG (0.589) MAFA (0.589) TCF12 (0.589) GCM1 (0.589) EGR4 (0.688) MYC (0.767) MTF1 (0.851) ARNTL (0.851) ZFP161 (0.864) TFAP4 (0.864) AHR (0.864) CREB3L2 (0.895) | BP: regulation of transforming growth factor beta receptor signaling pathway BP: negative regulation of signal transduction BP: Wnt receptor signaling pathway, calcium modulating pathway MF: transcription factor activity MF: potassium ion binding |
| 49 | 47 |  |  | 0.427 | MAX (0.427) MXI1 (0.427) TCF3 (0.427) NKX2-1 (0.427) SOHLH2 (0.427) CEBPZ (0.427) MESP1 (0.427) CLOCK (0.427) TAL1 (0.427) ATOH1 (0.427) FOXI1 (0.477) NEUROD1 (0.477) TWIST1 (0.507) NFYB (0.562) HEY2 (0.562) TWIST2 (0.562) NEUROG2 (0.562) ZNF238 (0.562) SNAI1 (0.599) SMAD1 (0.604) ENO1 (0.604) TCF21 (0.604) MYCN (0.604) ATOH8 (0.604) NFYC (0.604) BHLHE23 (0.604) BHLHE41 (0.604) | BP: sensory perception of smell BP: G-protein coupled receptor protein signaling pathway CC: extracellular space CC: keratin filament MF: olfactory receptor activity |
| 50 | 57 |  |  | 0.448 | SOHLH2 (0.448) | BP: potassium ion transport BP: neuron fate determination CC: integral to plasma membrane MF: potassium ion binding MF: cation channel activity |
| 51 | 14 |  |  | 0.450 | POU3F3 (0.450) ZNF232 (0.479) OTP (0.479) HLX (0.479) POU2F3 (0.479) NRL (0.479) POU5F1B (0.479) POU3F4 (0.479) POU2F2 (0.479) POU6F1 (0.566) MAFK (0.566) TBP (0.585) CUX2 (0.608) POU2F1 (0.608) ZNF32 (0.608) LHX5 (0.624) LHX1 (0.624) VSX1 (0.640) NKX3-1 (0.665) PROP1 (0.672) PAX4 (0.672) ARID3B (0.672) VAX1 (0.672) FUBP1 (0.672) FOXO1 (0.672) LHX4 (0.672) ZNF187 (0.672) NR1D1 (0.672) DMRTC2 (0.672) VAX2 (0.672) HOXD1 (0.672) HOXA9 (0.672) NOTO (0.672) RAX (0.672) HOXD8 (0.672) NOBOX (0.672) KDM2B (0.672) FOXL2 (0.672) LBX1 (0.672) SHOX (0.672) ALX3 (0.672) BARHL1 (0.672) RORC (0.672) POU3F2 (0.672) FOXP1 (0.672) LMX1B (0.672) FOXF2 (0.672) SHOX2 (0.672) ZFHX2 (0.672) EMX2 (0.672) LHX9 (0.672) LMX1A (0.672) RAX2 (0.672) PRRX2 (0.672) ZFHX3 (0.672) PDX1 (0.672) AC012531.1 (0.672) GBX1 (0.672) VENTX (0.672) LHX3 (0.672) EMX1 (0.672) HMG20B (0.672) ARX (0.672) GBX2 (0.672) RORA (0.672) TLX2 (0.672) GSX2 (0.672) MEOX1 (0.672) DBX2 (0.672) VTN (0.672) FOXB2 (0.672) GSX1 (0.672) ENSG00000229544 (0.672) POU4F1 (0.672) MSX1 (0.672) MEF2B (0.672) FOXD2 (0.672) HMGA1 (0.672) NKX1-1 (0.672) BCL6B (0.672) ISX (0.672) POU4F2 (0.672) MEF2D (0.672) CIC (0.672) ESX1 (0.672) ALX4 (0.672) DRGX (0.672) ZNF384 (0.672) SPIB (0.672) HAND1 (0.672) HOXB3 (0.672) HOXB5 (0.672) EN1 (0.672) PRRX1 (0.672) | BP: sensory perception of smell BP: G-protein coupled receptor protein signaling pathway BP: defense response BP: modification by symbiont of host morphology or physiology MF: olfactory receptor activity |
| 52 | 64 |  |  | 0.450 | SP2 (0.450) SP1 (0.450) SP3 (0.614) WT1 (0.614) EGR1 (0.614) ZNF148 (0.614) KLF5 (0.662) PRDM4 (0.662) RARB (0.662) MECP2 (0.662) STAT1 (0.662) E2F6 (0.662) ZBTB7A (0.662) STAT5A (0.662) PURA (0.750) ZNF281 (0.773) TFDP1 (0.773) SP5 (0.773) NR1I3 (0.773) GCM2 (0.773) ZFX (0.773) E2F8 (0.773) HSF1 (0.773) SP4 (0.773) CGBP (0.773) EGR4 (0.773) MBD2 (0.773) | CC: transcription factor complex CC: nucleolus CC: cytosol MF: zinc ion binding MF: ATP binding |
| 53 | 67 |  |  | 0.453 | WT1 (0.453) EGR1 (0.453) SP5 (0.453) EGR4 (0.453) ZNF148 (0.453) NR0B1 (0.453) SP1 (0.453) KLF16 (0.453) DMBX1 (0.453) NRF1 (0.453) ZBTB7B (0.453) TFAP2D (0.453) ZNF281 (0.453) NHLH1 (0.453) SP3 (0.453) KLF7 (0.453) MAZ (0.453) SP4 (0.453) DPRX (0.490) KLF5 (0.490) GSC (0.490) SP8 (0.508) SP2 (0.593) E2F1 (0.629) GLIS2 (0.629) | CC: nucleolus CC: cytosol MF: RNA binding MF: ATP binding MF: zinc ion binding |
| 54 | 49 |  |  | 0.506 | BHLHE40 (0.506) USF2 (0.506) TFAP2B (0.506) ATF3 (0.506) ETV6 (0.506) CREM (0.506) EGR4 (0.506) ELK4 (0.506) MECP2 (0.506) TFEB (0.506) HEY2 (0.506) ARNTL (0.506) BHLHE41 (0.506) HSF1 (0.506) MTF1 (0.506) CTCFL (0.638) ZFY (0.638) RFX2 (0.638) SP1 (0.638) E2F1 (0.638) USF1 (0.638) ELF3 (0.706) MBD2 (0.706) TFEC (0.706) THRB (0.706) KLF8 (0.706) SOHLH2 (0.706) EBF1 (0.706) MAFB (0.706) ZBTB7A (0.706) BACH1 (0.706) NEUROD1 (0.706) TLX1 (0.706) ZFX (0.706) PLAGL1 (0.706) INSM1 (0.706) ATF6 (0.706) TFAP2D (0.706) | BP: G1/S transition of mitotic cell cycle CC: nucleolus CC: cytosol MF: ATP binding MF: zinc ion binding |
| 55 | 68 |  |  | 0.508 | REST (0.508) | BP: regulation of blood pressure BP: organ morphogenesis BP: neuron differentiation CC: integral to plasma membrane MF: potassium ion binding |
| 56 | 3 |  |  | 0.510 | CREB3L2 (0.510) ENO1 (0.652) CLOCK (0.652) TWIST2 (0.652) TWIST1 (0.652) HEY1 (0.652) PKNOX2 (0.652) BACH1 (0.652) MITF (0.652) MYCN (0.652) MXI1 (0.652) HES2 (0.652) NEUROG2 (0.652) ZNF238 (0.652) ZEB1 (0.652) TGIF2LX (0.652) MLXIP (0.652) TBX18 (0.652) HES5 (0.652) NEUROD1 (0.652) ID2 (0.652) ID4 (0.652) MGA (0.652) PAX9 (0.652) TGIF2 (0.652) ATOH8 (0.652) ATF3 (0.652) TFE3 (0.652) HIF1A (0.652) ARNTL (0.652) TBX4 (0.652) PAX1 (0.652) MAX (0.652) USF2 (0.652) MYCL1 (0.652) | BP: cell-cell signaling BP: cell differentiation CC: extracellular space CC: integral to plasma membrane MF: tumor necrosis factor receptor activity |
| 57 | 107 |  |  | 0.521 | SPIC (0.521) SPIB (0.521) ELF1 (0.551) IRF2 (0.551) ETV6 (0.551) SPI1 (0.551) IRF9 (0.551) GABPA (0.551) ELF4 (0.551) PRDM1 (0.622) ZNF683 (0.622) IRF7 (0.741) IRF1 (0.741) | BP: anterior/posterior pattern formation BP: positive regulation of transcription from RNA polymerase II promoter BP: negative regulation of transcription from RNA polymerase II promoter BP: transmembrane receptor protein tyrosine kinase signaling pathway CC: transcription factor complex |
| 58 | 20 |  |  | 0.527 | ZBTB7B (0.527) ZNF263 (0.527) E2F6 (0.527) ZNF274 (0.527) MAZ (0.527) EOMES (0.527) ZBTB7A (0.527) ATF5 (0.527) ZNF148 (0.527) SP2 (0.527) E2F1 (0.527) ETV6 (0.527) ERF (0.551) ZNF281 (0.551) FLI1 (0.551) ETV2 (0.551) SP3 (0.613) SP1 (0.613) ZNF350 (0.613) ETV3 (0.613) MECP2 (0.613) IRF3 (0.613) ETS2 (0.613) ELK4 (0.613) ETV1 (0.613) CTCFL (0.613) KLF6 (0.613) | BP: inner ear morphogenesis BP: negative regulation of signal transduction CC: transcription factor complex CC: dendrite MF: transcription activator activity |
| 59 | 22 |  |  | 0.537 | ESR2 (0.537) FOXI1 (0.537) PGR (0.537) CEBPZ (0.537) ESR1 (0.613) SMAD1 (0.624) SMAD4 (0.649) HIC2 (0.649) GLI1 (0.723) YBX1 (0.723) SMAD3 (0.723) NEUROD1 (0.763) ZKSCAN3 (0.805) PBX3 (0.805) TFCP2L1 (0.805) GLI3 (0.805) ESRRA (0.805) TP63 (0.805) NFYB (0.805) MTF1 (0.805) | BP: cell volume homeostasis BP: cellular carbohydrate catabolic process BP: alcohol catabolic process BP: muscle contraction CC: extracellular space |
| 60 | 29 |  |  | 0.545 | RFX2 (0.545) RFX3 (0.545) MEIS3 (0.551) MAFK (0.551) MAFF (0.551) RFX4 (0.551) RFX6 (0.754) | CC: extracellular region |
| 61 | 13 |  |  | 0.572 | MAX (0.572) ID4 (0.572) NEUROD1 (0.572) SNAI1 (0.745) HESX1 (0.745) SMAD1 (0.745) NPAS2 (0.745) MYB (0.745) ATOH1 (0.745) MYOD1 (0.821) MYF6 (0.857) HEY1 (0.857) MYCN (0.857) DNMT1 (0.857) KLF15 (0.857) TFAP2B (0.857) HES5 (0.857) MNT (0.857) HES7 (0.857) | BP: immune response CC: extracellular space MF: phospholipase A2 activity MF: serine-type endopeptidase activity MF: bacterial cell surface binding |
| 62 | 59 |  |  | 0.587 | CEBPA (0.587) CEBPE (0.693) | BP: sensory perception of smell BP: G-protein coupled receptor protein signaling pathway BP: inflammatory response MF: olfactory receptor activity MF: taste receptor activity |
| 63 | 80 |  |  | 0.609 | EOMES (0.609) USF2 (0.609) | CC: transcription factor complex CC: cytosol CC: nucleolus MF: ATP binding MF: zinc ion binding |
| 64 | 10 |  |  | 0.619 | CXXC1 (0.619) | BP: small GTPase mediated signal transduction MF: RNA binding MF: transcription factor activity MF: ATP binding MF: sequence-specific DNA binding |
| 65 | 101 |  |  | 0.704 | NR1H4 (0.704) EGR2 (0.704) KLF7 (0.704) KLF12 (0.704) KLF14 (0.704) PURA (0.704) NRF1 (0.814) SRF (0.814) TFAP2C (0.814) SP5 (0.814) KLF2 (0.814) YBX1 (0.814) EGR1 (0.814) NKX2-8 (0.814) SIM1 (0.814) SP1 (0.814) ZIC2 (0.823) KLF4 (0.841) SP8 (0.891) NR5A2 (0.894) KLF5 (0.894) ZNF281 (0.894) ESR1 (0.894) TCF15 (0.894) BHLHE40 (0.894) | BP: regulation of cell communication BP: small GTPase mediated signal transduction BP: prostaglandin metabolic process CC: lysosome MF: potassium ion binding |
| 66 | 6 |  |  | 0.706 | MBD2 (0.706) | BP: nuclear mRNA splicing, via spliceosome BP: transcription from RNA polymerase II promoter BP: protein transport CC: nucleolus MF: structural constituent of ribosome |
| 67 | 53 |  |  | 0.711 | ETV6 (0.711) MBD2 (0.711) DNMT1 (0.711) | CC: nucleolus CC: cytosol CC: transcription factor complex MF: ATP binding MF: zinc ion binding |
| 68 | 39 |  |  | 0.714 | MAF (0.714) ARNT2 (0.714) MTF1 (0.714) | BP: regulation of transcription, DNA-dependent BP: neuron fate commitment CC: nucleus MF: transcription factor activity MF: sequence-specific DNA binding |
| 69 | 122 |  |  | 0.731 | MBD2 (0.731) | CC: transcription factor complex CC: nucleolus CC: cytosol MF: ATP binding MF: zinc ion binding |
| 70 | 125 |  |  | 0.772 | PLAGL1 (0.772) NEUROD1 (0.874) SMAD1 (0.874) ZIC4 (0.874) | BP: alcohol metabolic process BP: regulation of signal transduction BP: organ morphogenesis CC: stress fiber MF: sequence-specific DNA binding |
| 71 | 109 |  |  | 0.787 | MZF1 (0.787) HIC2 (0.787) ZNF202 (0.787) GLIS3 (0.787) PRDM4 (0.787) NR0B1 (0.787) KLF1 (0.787) ZNF219 (0.787) REL (0.787) PURA (0.787) KLF3 (0.787) PLAG1 (0.787) ZBTB7B (0.787) | BP: calcium ion transport BP: muscle contraction CC: integral to plasma membrane CC: extracellular space MF: potassium ion binding |
| 72 | 111 |  |  | 0.791 | MBD2 (0.791) ZBED1 (0.791) USF2 (0.791) BRCA1 (0.791) ATF3 (0.791) HES1 (0.791) E4F1 (0.791) CREB1 (0.791) ZBTB33 (0.791) TCFL5 (0.791) TFEC (0.791) MTF1 (0.791) ATF1 (0.791) SMAD4 (0.791) MLX (0.791) E2F3 (0.791) TFDP1 (0.791) ATF6 (0.791) BACH1 (0.791) GLIS2 (0.791) BHLHE40 (0.791) SMAD3 (0.822) E2F2 (0.822) E2F8 (0.822) CREB3L1 (0.891) | BP: DNA repair CC: nucleolus CC: transcription factor complex MF: ATP binding MF: protein serine/threonine kinase activity |
| 73 | 103 |  |  | 0.795 | SP4 (0.795) SP3 (0.795) DNMT1 (0.795) PLAGL1 (0.795) ZFP161 (0.795) NRF1 (0.795) SP1 (0.795) GLIS2 (0.795) MBD2 (0.795) E2F3 (0.795) KLF5 (0.795) E2F6 (0.795) ZBTB7B (0.795) MECP2 (0.795) E2F2 (0.795) EGR4 (0.795) TFAP2B (0.795) HES1 (0.795) EGR1 (0.795) NFIA (0.795) NFIX (0.795) USF2 (0.795) ZIC5 (0.795) SP2 (0.795) KLF4 (0.842) | BP: negative regulation of signal transduction CC: transcription factor complex CC: nucleolus CC: cytosol MF: ATP binding |
| 74 | 44 |  |  | 0.811 | WT1 (0.811) | BP: nuclear mRNA splicing, via spliceosome CC: nucleolus CC: transcription factor complex MF: ATP binding MF: structural constituent of ribosome |
| 75 | 124 |  |  | 0.844 | ZNF75A (0.844) | CC: nucleolus CC: transcription factor complex MF: ATP binding MF: structural constituent of ribosome MF: zinc ion binding |
| 76 | 42 |  |  | 0.853 | ZBTB6 (0.853) PLAGL1 (0.853) NR1H4 (0.853) | BP: small GTPase mediated signal transduction BP: cell development BP: actin cytoskeleton organization CC: cytoplasmic part MF: potassium channel activity |
| 77 | 76 |  |  | 0.882 | HLTF (0.882) MAFA (0.882) E2F6 (0.882) | BP: Wnt receptor signaling pathway, calcium modulating pathway CC: integral to plasma membrane CC: proteinaceous extracellular matrix MF: transcription factor activity MF: sequence-specific DNA binding |
| 78 | 54 |  |  | 0.892 | SOHLH2 (0.892) | BP: negative regulation of blood pressure CC: plasma membrane part CC: cytoplasm MF: potassium ion binding MF: calcium ion binding |
| 79 | 0 |  |  | NA |  | BP: cell volume homeostasis CC: cytoplasm CC: extracellular space MF: potassium ion binding MF: symporter activity |
| 80 | 1 |  |  | NA |  | BP: cell division BP: rRNA processing CC: nucleolus CC: spliceosomal complex MF: translation regulator activity |
| 81 | 2 |  |  | NA |  | BP: axon guidance CC: transcription factor complex CC: cytosol CC: nucleolus MF: ATP binding |
| 82 | 5 |  |  | NA |  | CC: nucleolus CC: transcription factor complex CC: cytosol MF: ATP binding MF: zinc ion binding |
| 83 | 7 |  |  | NA |  | BP: G-protein coupled receptor protein signaling pathway CC: extracellular space CC: myosin complex MF: cytokine activity MF: calcium ion binding |
| 84 | 8 |  |  | NA |  |  |
| 85 | 11 |  |  | NA |  | BP: muscle contraction BP: metal ion transport CC: anchored to membrane MF: receptor binding MF: hydrolase activity, acting on carbon-nitrogen (but not peptide) bonds, in linear amidines |
| 86 | 19 |  |  | NA |  | CC: extracellular region |
| 87 | 21 |  |  | NA |  | BP: sensory perception of smell BP: immune response CC: integral to membrane CC: nucleosome MF: olfactory receptor activity |
| 88 | 23 |  |  | NA |  | BP: sensory perception of smell BP: G-protein coupled receptor protein signaling pathway CC: plasma membrane MF: olfactory receptor activity MF: serine-type endopeptidase activity |
| 89 | 24 |  |  | NA |  | BP: pattern specification process MF: transcription factor activity MF: sequence-specific DNA binding MF: protein homodimerization activity MF: potassium ion binding |
| 90 | 25 |  |  | NA |  | BP: sensory perception of smell BP: G-protein coupled receptor protein signaling pathway BP: inflammatory response CC: extracellular region MF: olfactory receptor activity |
| 91 | 26 |  |  | NA |  | BP: sensory perception of smell BP: G-protein coupled receptor protein signaling pathway BP: cellular macromolecule biosynthetic process BP: RNA splicing MF: olfactory receptor activity |
| 92 | 28 |  |  | NA |  | BP: sensory perception of smell MF: olfactory receptor activity |
| 93 | 30 |  |  | NA |  | BP: transport BP: monosaccharide metabolic process BP: alcohol catabolic process CC: plasma membrane part MF: potassium ion binding |
| 94 | 34 |  |  | NA |  | BP: cell division BP: anterior/posterior pattern formation CC: nucleolus CC: spliceosomal complex MF: ATP binding |
| 95 | 37 |  |  | NA |  | CC: keratin filament CC: integral to plasma membrane MF: sequence-specific DNA binding MF: transcription factor activity MF: potassium ion binding |
| 96 | 38 |  |  | NA |  | BP: small GTPase mediated signal transduction BP: anterior/posterior pattern formation MF: transcription factor activity MF: sequence-specific DNA binding MF: transcription activator activity |
| 97 | 41 |  |  | NA |  | BP: sensory perception of smell BP: G-protein coupled receptor protein signaling pathway BP: defense response to bacterium MF: olfactory receptor activity MF: serine-type endopeptidase inhibitor activity |
| 98 | 46 |  |  | NA |  | CC: mitochondrion |
| 99 | 51 |  |  | NA |  | BP: cell-cell signaling MF: transcription factor activity MF: sequence-specific DNA binding MF: potassium ion binding MF: serine-type endopeptidase activity |
| 100 | 52 |  |  | NA |  |  |
| 101 | 61 |  |  | NA |  | BP: immune response BP: lipid catabolic process CC: extracellular space CC: integral to membrane MF: calcium ion binding |
| 102 | 62 |  |  | NA |  | BP: neuron fate commitment CC: cytosol CC: nucleolus MF: transcription activator activity MF: ATP binding |
| 103 | 63 |  |  | NA |  | BP: translation BP: ribonucleoprotein complex biogenesis BP: ncRNA processing BP: rRNA metabolic process BP: transcription from RNA polymerase III promoter |
| 104 | 65 |  |  | NA |  | BP: potassium ion transport BP: protein amino acid phosphorylation MF: transcription factor activity MF: potassium ion binding MF: sequence-specific DNA binding |
| 105 | 66 |  |  | NA |  | BP: positive regulation of transcription from RNA polymerase II promoter BP: potassium ion transport MF: transcription factor activity MF: sequence-specific DNA binding MF: potassium ion binding |
| 106 | 70 |  |  | NA |  | BP: glucose homeostasis BP: cellular metabolic process CC: Golgi apparatus MF: potassium ion binding MF: transcription factor activity |
| 107 | 72 |  |  | NA |  | BP: humoral immune response BP: innate immune response BP: regulation of acute inflammatory response MF: hormone activity MF: bacterial cell surface binding |
| 108 | 73 |  |  | NA |  | BP: cellular component biogenesis BP: RNA processing CC: nucleolus CC: chromosome CC: ribonucleoprotein complex |
| 109 | 75 |  |  | NA |  | BP: sensory perception of smell BP: translation BP: nuclear mRNA splicing, via spliceosome CC: nucleosome MF: olfactory receptor activity |
| 110 | 77 |  |  | NA |  | CC: nucleolus CC: cytosol CC: transcription factor complex MF: ATP binding MF: zinc ion binding |
| 111 | 79 |  |  | NA |  | CC: nucleolus CC: transcription factor complex CC: cytosol MF: ATP binding MF: zinc ion binding |
| 112 | 81 |  |  | NA |  | BP: small GTPase mediated signal transduction BP: muscle contraction CC: stress fiber CC: cytoplasmic part MF: potassium ion binding |
| 113 | 82 |  |  | NA |  | BP: negative regulation of signal transduction CC: nucleolus CC: transcription factor complex MF: ATP binding MF: zinc ion binding |
| 114 | 87 |  |  | NA |  | CC: nucleolus CC: cytosol CC: spliceosomal complex MF: zinc ion binding MF: ATP binding |
| 115 | 90 |  |  | NA |  | CC: transcription factor complex CC: cytosol CC: nucleolus MF: RNA binding MF: specific transcriptional repressor activity |
| 116 | 93 |  |  | NA |  | CC: nucleolus CC: transcription factor complex MF: ATP binding MF: zinc ion binding MF: double-stranded DNA binding |
| 117 | 94 |  |  | NA |  | BP: negative regulation of gene-specific transcription from RNA polymerase II promoter BP: negative regulation of signal transduction CC: transcription factor complex MF: ATP binding MF: potassium ion binding |
| 118 | 96 |  |  | NA |  | BP: muscle contraction CC: plasma membrane part CC: extracellular space MF: calcium ion binding MF: cation transmembrane transporter activity |
| 119 | 98 |  |  | NA |  | CC: nucleolus CC: cytosol MF: transcription factor activity MF: sequence-specific DNA binding MF: RNA binding |
| 120 | 100 |  |  | NA |  | BP: anterior/posterior pattern formation CC: cytosol MF: transcription factor activity MF: transcription activator activity MF: RNA polymerase II transcription factor activity |
| 121 | 104 |  |  | NA |  | BP: sensory perception of smell BP: G-protein coupled receptor protein signaling pathway BP: inflammatory response BP: immune response MF: olfactory receptor activity |
| 122 | 110 |  |  | NA |  | BP: peripheral nervous system development CC: transcription factor complex CC: cytosol CC: nucleolus MF: ATP binding |
| 123 | 112 |  |  | NA |  | BP: immune response CC: high-density lipoprotein particle CC: integral to plasma membrane MF: G-protein coupled receptor activity MF: serine-type endopeptidase activity |
| 124 | 113 |  |  | NA |  | BP: negative regulation of signal transduction CC: transcription factor complex MF: transcription activator activity MF: zinc ion binding MF: ATP binding |
| 125 | 115 |  |  | NA |  | CC: integral to membrane CC: plasma membrane part CC: extracellular region MF: aspartic-type endopeptidase activity MF: phospholipase A2 activity |
| 126 | 120 |  |  | NA |  | BP: potassium ion transport BP: fibroblast growth factor receptor signaling pathway MF: protein homodimerization activity MF: potassium ion binding MF: growth factor activity |
| 127 | 123 |  |  | NA |  | BP: sensory perception of smell BP: G-protein coupled receptor protein signaling pathway CC: extracellular space CC: plasma membrane MF: olfactory receptor activity |
| 128 | 127 |  |  | NA |  | BP: nuclear mRNA splicing, via spliceosome CC: nucleolus MF: ATP binding MF: structural constituent of ribosome MF: zinc ion binding |
