# Supplementary material for: DeepCpG: accurate prediction of single-cell DNA methylation states using deep learning
Source: Genome Biol. 2017 Apr 11;18:67. doi: 10.1186/s13059-017-1189-z (PMC5387360; doi:10.1186/s13059-017-1189-z)
Supplement: Supplementary file 3 — Sequence motifs. HTML files with sequence logos and summary statistics for all cell types. (ZIP 19482 kb) [file 13059_2017_1189_MOESM3_ESM.zip › mESC.html]

Filter table


# Filter table

#### *Christof Angermueller*

#### *2016-12-17*

Nr | Label | Logo | Influence | q-value | Related known motifs | GO annotations || 1 | 2: Zfp161 |  |  | 0.003 | Zfp161 (0.003) E2f1 (0.003) Plagl1 (0.025) Zfp161 (0.027) E2F3 (0.048) Sp3 (0.070) Hes7 (0.070) Hes1 (0.070) Sp1 (0.070) Zbtb7a (0.070) Zfx (0.074) E2F2 (0.088) Wt1 (0.096) Tcfap2d (0.106) Tcfl5 (0.106) Hey1 (0.106) Ctcfl (0.118) Plagl1 (0.121) Klf6 (0.123) E2f3 (0.123) Zfp219 (0.136) Egr4 (0.137) Plagl1 (0.137) Klf16 (0.137) Hes5 (0.172) | CC: transcription factor complex MF: ATP binding MF: zinc ion binding MF: transcription activator activity MF: magnesium ion binding |
| 2 | 102: Tcfap2d |  |  | 0.004 | Tcfap2d (0.004) Zic1 (0.136) Zfp161 (0.136) Mbd2 (0.136) Zic2 (0.136) Zic3 (0.136) Tcfap2c (0.136) Tcfap2a (0.136) Zic2 (0.136) Nr2f2 (0.136) Sp3 (0.139) Zfx (0.139) Zic4 (0.139) Wt1 (0.151) Zic1 (0.151) Tcfap2b (0.151) Zic3 (0.169) Ctcfl (0.172) Tcfap2e (0.175) Zic2 (0.204) Zbtb49 (0.230) E2f3 (0.242) Egr3 (0.242) Tcfap2b (0.250) Sp1 (0.258) E2f1 (0.258) | CC: transcription factor complex MF: ATP binding MF: zinc ion binding MF: magnesium ion binding MF: manganese ion binding |
| 3 | 64: Zfp410 |  |  | 0.009 | Zfp410 (0.009) Mbd2 (0.009) Wt1 (0.054) Zfp281 (0.070) Sp3 (0.070) Bcl6b (0.073) Zfp740 (0.095) Maz (0.100) E2f4 (0.100) Tcfap2c (0.100) Egr4 (0.100) E2f1 (0.100) Sp1 (0.100) E2f3 (0.107) Tcfap2d (0.107) E2f6 (0.107) Zfp281 (0.125) Zfp524 (0.125) Zfp148 (0.125) Nr2f2 (0.202) Klf6 (0.209) Zbtb7a (0.209) Egr2 (0.228) Klf15 (0.228) Zfx (0.228) Klf5 (0.228) | BP: transcription BP: negative regulation of transcription from RNA polymerase II promoter MF: ATP binding MF: zinc ion binding MF: transcription activator activity |
| 4 | 20: Zic1 |  |  | 0.013 | Zic1 (0.013) Zic3 (0.013) Zic2 (0.013) Ets2 (0.015) Elf1 (0.015) Gabpa (0.015) Zic5 (0.039) Nr2c2 (0.039) Zfp281 (0.039) Tcfap2c (0.044) Tcfap2a (0.044) Tcfap2a (0.044) Tcfap2c (0.045) Tcfap2d (0.067) Wt1 (0.069) Ctcfl (0.071) Ehf (0.104) Tcfap2b (0.105) Klf15 (0.105) Tcfap2b (0.106) Egr1 (0.106) Mbd2 (0.107) Erf (0.107) E2f4 (0.128) Egr1 (0.139) Egr4 (0.139) E2f6 (0.139) Tcfap2e (0.139) | BP: negative regulation of transcription from RNA polymerase II promoter CC: transcription factor complex MF: ATP binding MF: zinc ion binding MF: chromatin binding |
| 5 | 116: Zscan4 |  |  | 0.017 | Zscan4 (0.017) Zscan4 (0.027) Gm397 (0.036) Zscan4c (0.068) Zfp691 (0.418) Zkscan1 (0.518) | BP: sensory perception of smell CC: extracellular space CC: external side of plasma membrane MF: olfactory receptor activity MF: calcium ion binding |
| 6 | 42: Sp3 |  |  | 0.018 | Sp3 (0.018) Mbd2 (0.048) Wt1 (0.074) Ctcfl (0.090) Tcfap2d (0.090) E2f4 (0.090) Zfx (0.090) Zic1 (0.090) E2f1 (0.090) Klf5 (0.090) Bcl6b (0.090) Zic3 (0.090) Zic2 (0.090) Egr4 (0.090) Zfp410 (0.090) Zbtb49 (0.090) Zfp219 (0.096) Smad3 (0.096) E2f3 (0.098) Zfp281 (0.098) Klf4 (0.099) Klf2 (0.099) Klf7 (0.099) Tcfap2c (0.101) Maz (0.117) E2f6 (0.117) Klf12 (0.117) Zic5 (0.117) Sp1 (0.117) | BP: transcription BP: protein amino acid phosphorylation CC: transcription factor complex MF: ATP binding MF: zinc ion binding |
| 7 | 75: Sp3 |  |  | 0.022 | Sp3 (0.022) Klf5 (0.022) Bcl6b (0.022) Klf6 (0.022) Smad3 (0.022) Sp5 (0.022) Nhlh1 (0.054) Klf16 (0.056) Klf7 (0.061) Maz (0.069) Klf4 (0.069) Egr4 (0.069) Zfp410 (0.069) Wt1 (0.069) Klf12 (0.069) Sp4 (0.069) Zfp148 (0.069) Klf8 (0.074) Plagl1 (0.075) Zfp219 (0.075) Sp1 (0.081) Zfp281 (0.099) E2f4 (0.099) Sp4 (0.099) E2f1 (0.099) Sp8 (0.099) | BP: transcription MF: ATP binding MF: zinc ion binding MF: magnesium ion binding MF: manganese ion binding |
| 8 | 62: Tcfap2c |  |  | 0.023 | Tcfap2c (0.023) Zic4 (0.031) Zic1 (0.031) Zic3 (0.040) Zic2 (0.040) Zfp740 (0.064) Zfx (0.064) Tcfap2a (0.098) Tcfap2d (0.142) Ctcfl (0.142) Glis3 (0.142) Wt1 (0.142) Egr4 (0.142) Zic1 (0.142) Tcfap2a (0.142) Mbd2 (0.142) Zic2 (0.142) Sp3 (0.142) Gcm1 (0.171) Zic5 (0.178) Zfp161 (0.184) Glis1 (0.187) Tcfap2c (0.188) Zic3 (0.189) Bcl6b (0.189) Glis2 (0.189) Ebf1 (0.189) | BP: mRNA processing BP: RNA splicing CC: transcription factor complex MF: ATP binding MF: zinc ion binding |
| 9 | 122: Mbd2 |  |  | 0.023 | Mbd2 (0.023) Klf6 (0.348) Sp2 (0.401) Wt1 (0.401) Sp3 (0.401) Tcfap2d (0.401) Bcl6b (0.403) Zfx (0.403) Plagl1 (0.403) Plagl1 (0.450) E2f1 (0.450) Sp1 (0.450) Zbtb49 (0.469) Smad3 (0.469) Smad2 (0.522) E2f3 (0.522) Egr4 (0.610) Zfp219 (0.615) Myf6 (0.617) Zfp161 (0.653) Gli3 (0.678) Ctcfl (0.678) Nr2f2 (0.678) E2f4 (0.678) Zbtb7a (0.756) Klf5 (0.756) Zic5 (0.756) Sp4 (0.756) Gli1 (0.756) Ybx1 (0.756) Smad4 (0.756) Egr1 (0.756) E2f6 (0.756) Pbx3 (0.756) Klf16 (0.756) Klf1 (0.756) Myod1 (0.756) Etv4 (0.756) Zfp161 (0.756) Glis2 (0.756) Egr3 (0.756) Mtf1 (0.756) Zbtb7c (0.756) Zfp410 (0.756) Etv1 (0.756) Gm5454 (0.756) Zkscan4 (0.756) Glis1 (0.756) E2F3 (0.756) Hes7 (0.756) E2f5 (0.756) Gli2 (0.756) Klf2 (0.756) Maz (0.756) | BP: negative regulation of transcription from RNA polymerase II promoter CC: transcription factor complex CC: endoplasmic reticulum MF: ATP binding MF: zinc ion binding |
| 10 | 121: Ctcfl |  |  | 0.026 | Ctcfl (0.026) Tcfap2d (0.026) Mbd2 (0.026) Sp3 (0.030) Egr4 (0.035) Wt1 (0.035) Klf6 (0.037) Zfx (0.043) Zbtb7a (0.053) Klf16 (0.085) E2f1 (0.085) Ctcf (0.087) Tcfap2a (0.087) Sp2 (0.106) Zic1 (0.112) Zfp219 (0.123) Tcfap2c (0.123) Egr1 (0.123) Zic3 (0.126) Sp5 (0.126) Zic2 (0.136) Zic4 (0.148) Nr2c2 (0.148) E2f3 (0.148) Egr1 (0.157) Klf5 (0.157) Sp1 (0.157) Sp8 (0.157) Maz (0.157) Nhlh1 (0.157) Egr2 (0.157) Elf1 (0.157) Zic1 (0.157) Zic2 (0.157) Ets2 (0.157) | BP: transcription BP: negative regulation of transcription from RNA polymerase II promoter CC: transcription factor complex MF: ATP binding MF: zinc ion binding |
| 11 | 40: Klf5 |  |  | 0.029 | Klf5 (0.029) Klf15 (0.029) Sp3 (0.042) Zbtb7a (0.046) Maz (0.112) Klf16 (0.112) Smad3 (0.112) Klf4 (0.123) Zfp281 (0.124) Klf7 (0.124) Zfp740 (0.124) Zfp148 (0.124) Klf8 (0.124) Klf12 (0.124) Bcl6b (0.124) Ctcfl (0.124) Zfp281 (0.124) Tcfap2d (0.131) Sp5 (0.139) Egr4 (0.139) Sp8 (0.149) Klf2 (0.169) Sp4 (0.179) Zfp410 (0.214) Ybx1 (0.244) Sp1 (0.244) | BP: transcription BP: protein amino acid phosphorylation BP: negative regulation of transcription from RNA polymerase II promoter MF: zinc ion binding MF: ATP binding |
| 12 | 67: Wt1 |  |  | 0.030 | Wt1 (0.030) E2f3 (0.030) Tcfap2d (0.035) Nr2f2 (0.036) Ctcfl (0.038) E2f1 (0.043) Egr1 (0.074) Plagl1 (0.095) Zfp148 (0.095) Zfx (0.095) Insm1 (0.095) Sp1 (0.095) Klf6 (0.095) Sp3 (0.095) Rara (0.121) Egr1 (0.126) Egr2 (0.139) Smad3 (0.163) Klf16 (0.163) Zfp161 (0.197) Zfp281 (0.197) Egr3 (0.235) Zfp161 (0.235) Zbtb7a (0.268) E2F3 (0.268) | BP: negative regulation of transcription from RNA polymerase II promoter CC: transcription factor complex MF: ATP binding MF: zinc ion binding MF: magnesium ion binding |
| 13 | 26: E2f3 |  |  | 0.037 | E2f3 (0.037) Mbd2 (0.180) Sp3 (0.180) Maz (0.234) E2f6 (0.234) Wt1 (0.234) Klf6 (0.239) Hnf4g (0.293) Nhlh1 (0.293) Sp1 (0.293) Zfp219 (0.329) Sp4 (0.329) Egr1 (0.329) Bcl6b (0.329) Zfp281 (0.353) Sp5 (0.353) E2f4 (0.384) Zfp410 (0.433) Elk3 (0.433) Elk4 (0.433) Elk1 (0.438) Etv4 (0.438) Gm5454 (0.438) Zfp148 (0.438) Etv1 (0.438) Fli1 (0.438) Etv6 (0.438) E2f1 (0.438) Zfp281 (0.438) | BP: mRNA processing BP: RNA splicing CC: transcription factor complex MF: ATP binding MF: zinc ion binding |
| 14 | 35: Sp2 |  |  | 0.037 | Sp2 (0.037) Sp3 (0.050) Wt1 (0.149) E2f3 (0.176) Egr1 (0.176) Zic3 (0.375) Egr3 (0.375) Bcl6b (0.375) Mbd2 (0.375) Klf6 (0.399) E2f1 (0.399) Egr1 (0.420) Zfp410 (0.454) Smad3 (0.454) Nhlh1 (0.471) Zic4 (0.602) Zfx (0.613) Tcfap2b (0.613) Spdef (0.613) Sp1 (0.613) Klf7 (0.702) Pgr (0.702) Sp4 (0.702) Cutl1 (0.702) Nhlh2 (0.702) Scrt1 (0.702) | BP: transcription BP: negative regulation of transcription from RNA polymerase II promoter MF: ATP binding MF: zinc ion binding MF: GTP binding |
| 15 | 86: Sp3 |  |  | 0.043 | Sp3 (0.043) Egr4 (0.325) E2f4 (0.500) Zfx (0.500) E2f6 (0.500) Gabpa (0.630) Zbtb49 (0.630) Mbd2 (0.630) E2f3 (0.630) Wt1 (0.630) Zfp161 (0.630) Gm98 (0.630) E2f1 (0.630) Egr1 (0.636) Klf7 (0.712) Nr2f2 (0.712) Ctcfl (0.712) Tcf15 (0.712) Klf6 (0.712) Sp1 (0.712) Smad3 (0.712) Sohlh2 (0.712) Klf12 (0.712) Tcfap2d (0.712) Klf4 (0.712) Gcm1 (0.712) Mtf1 (0.712) Klf5 (0.712) Zic1 (0.712) Klf16 (0.712) Klf14 (0.712) Sp2 (0.712) Atf6 (0.712) Zfp161 (0.712) Zic5 (0.712) Klf2 (0.712) Zfp219 (0.712) Zfp281 (0.712) Zfp128 (0.712) Bcl6b (0.712) Klf7 (0.712) Zic2 (0.712) Hes2 (0.712) Sp5 (0.712) Zbtb3 (0.712) Egr3 (0.712) Klf8 (0.712) Sp4 (0.712) Zfp161 (0.712) Maz (0.712) Zfp148 (0.712) Zic3 (0.712) Zfp740 (0.712) Plagl1 (0.712) Sp4 (0.712) Zfp410 (0.712) | BP: transcription BP: negative regulation of transcription from RNA polymerase II promoter CC: transcription factor complex MF: ATP binding MF: zinc ion binding |
| 16 | 16: Ebf1 |  |  | 0.044 | Ebf1 (0.044) Rfx4 (0.044) Rfx6 (0.044) Rfx3 (0.243) Rfxdc2 (0.244) Rfx3 (0.371) Rfx4 (0.371) Ctcfl (0.393) Tcfap2c (0.676) Smad1 (0.694) Cenpb (0.694) Tcfap2a (0.694) Klf3 (0.694) Rfx2 (0.694) Rfx7 (0.694) Sp2 (0.694) Tbx2 (0.694) Zic3 (0.694) Zic1 (0.694) Zic2 (0.694) Zfp524 (0.694) Tcfap2d (0.694) Arid2 (0.694) Smarcc2 (0.694) E2f6 (0.698) | BP: transcription BP: negative regulation of transcription from RNA polymerase II promoter BP: angiogenesis BP: neuron fate commitment BP: protein amino acid phosphorylation |
| 17 | 34 |  |  | 0.052 | Zfp161 (0.052) Mafa (0.286) Zfx (0.425) | BP: transcription MF: transcription factor activity MF: sequence-specific DNA binding MF: ATP binding MF: zinc ion binding |
| 18 | 109 |  |  | 0.078 | Esr2 (0.078) Zfp369 (0.078) | BP: defense response to bacterium CC: extracellular space MF: peptide receptor activity, G-protein coupled MF: cytokine activity MF: calcium ion binding |
| 19 | 94 |  |  | 0.080 | Klf7 (0.080) Eomes (0.119) Zbtb7a (0.139) Sp3 (0.243) Sp4 (0.271) Klf6 (0.271) Zfp410 (0.271) Zfp263 (0.335) Ascl2 (0.335) Gmeb1 (0.335) Ctcfl (0.439) Klf2 (0.512) Zfp148 (0.512) Nhlh1 (0.512) Sp5 (0.512) Sp1 (0.512) Esrra (0.512) Fos (0.512) Egr1 (0.512) Klf7 (0.512) Neurod1 (0.512) Elf1 (0.512) Klf12 (0.512) Zfx (0.512) Zfp281 (0.512) Id4 (0.512) Klf8 (0.512) Klf5 (0.512) Gabpa (0.512) | BP: negative regulation of transcription from RNA polymerase II promoter BP: modification-dependent protein catabolic process MF: zinc ion binding MF: ATP binding MF: transcription activator activity |
| 20 | 5 |  |  | 0.086 | Egr4 (0.086) Zfx (0.086) Egr1 (0.086) Wt1 (0.095) Egr1 (0.152) Mbd2 (0.200) Egr3 (0.214) Sp3 (0.214) Klf5 (0.238) Tcfap2d (0.241) E2f3 (0.243) Egr2 (0.243) Plagl1 (0.243) Klf16 (0.243) Smad3 (0.243) Klf6 (0.258) Zfp219 (0.383) Zfp161 (0.383) Tcfap2b (0.429) Nr2f2 (0.429) Tcfap2c (0.429) Egr1 (0.429) Sp5 (0.429) Smad2 (0.429) Nhlh1 (0.429) | BP: cell division BP: negative regulation of transcription from RNA polymerase II promoter MF: transcription factor activity MF: ATP binding MF: zinc ion binding |
| 21 | 50 |  |  | 0.090 | Sp3 (0.090) Wt1 (0.169) Klf6 (0.169) Eomes (0.169) Zfx (0.169) Egr4 (0.169) Klf15 (0.169) Sp4 (0.169) E2f3 (0.169) Egr1 (0.210) Smad3 (0.292) Klf16 (0.300) Ctcfl (0.300) Gabpa (0.300) Zfp219 (0.300) Egr1 (0.300) Plagl1 (0.300) Zbtb7a (0.300) Zfp148 (0.300) Mafa (0.300) Mbd2 (0.300) Sp1 (0.300) Egr3 (0.300) Tcfap2b (0.325) E2f1 (0.362) Zfp281 (0.362) Myog (0.362) Gm98 (0.362) Sp4 (0.362) | BP: transcription BP: positive regulation of transcription from RNA polymerase II promoter BP: negative regulation of transcription from RNA polymerase II promoter MF: zinc ion binding MF: ATP binding |
| 22 | 52 |  |  | 0.093 | Esrrb (0.093) Esr2 (0.206) Rorb (0.357) Rarb (0.360) Zbtb7b (0.487) Rxrg (0.558) Rxrb (0.558) Esrra (0.558) Rfx2 (0.558) Rara (0.558) Nr2f1 (0.558) Rarg (0.558) Rara (0.558) Gli1 (0.558) Atf3 (0.558) Esrrg (0.593) Arid2 (0.593) Zbtb7b (0.593) Nr2f2 (0.593) Plagl1 (0.593) Esrra (0.596) Gli3 (0.607) Nr2c1 (0.617) Creb3 (0.617) Mbtps2 (0.617) Nr5a2 (0.617) | BP: icosanoid biosynthetic process CC: lysosome CC: cytoskeletal part MF: neutral amino acid transmembrane transporter activity MF: serine-type endopeptidase activity |
| 23 | 89 |  |  | 0.094 | E2f4 (0.094) Maz (0.094) Sp3 (0.116) E2f6 (0.116) Zfp740 (0.134) Klf15 (0.224) Zfx (0.224) Zfp281 (0.224) Tcfap2d (0.224) Egr4 (0.224) Zic4 (0.315) Mbd2 (0.324) Wt1 (0.324) Zfp281 (0.324) Zic2 (0.324) E2f3 (0.335) Zfp148 (0.338) Zic1 (0.372) Bcl6b (0.382) Thrb (0.382) Tcfap2a (0.382) Zic3 (0.382) Smad3 (0.411) Zfp410 (0.411) Nr2f2 (0.419) Ctcfl (0.419) | BP: transcription BP: protein amino acid phosphorylation CC: transcription factor complex MF: transcription activator activity MF: zinc ion binding |
| 24 | 14 |  |  | 0.095 | Ahctf1 (0.095) Sox13 (0.739) Hlx1 (0.739) Lmx1b (0.739) Isl2 (0.739) Msx3 (0.739) Sox15 (0.739) Pou3f2 (0.739) Sox5 (0.739) Hoxc6 (0.739) Hoxb13 (0.739) Barhl1 (0.739) Otp (0.739) Sox12 (0.739) Arid3b (0.739) Sox30 (0.739) Pax6 (0.739) Dmrtc2 (0.739) Lhx1 (0.739) Msx1 (0.739) Sox17 (0.739) Pou3f4 (0.739) Cdx4 (0.739) Uncx4.1 (0.739) Prop1 (0.739) Foxd4 (0.739) Sebox (0.739) Phf21a (0.739) Nkx6-3 (0.739) Pou1f1 (0.739) Sox7 (0.739) Pax7 (0.739) Nkx2-5 (0.739) Elf3 (0.739) Nkx6-1 (0.739) Sox8 (0.739) Hoxa4 (0.739) Barhl2 (0.739) Dbx2 (0.739) Dmrt2 (0.739) Dbx1 (0.739) Hoxd10 (0.739) Cart1 (0.739) | BP: G-protein coupled receptor protein signaling pathway BP: sensory perception of smell BP: signal transduction BP: cell communication MF: olfactory receptor activity |
| 25 | 49 |  |  | 0.096 | Wt1 (0.096) Smad3 (0.096) Sp3 (0.096) Zbtb1 (0.365) Rfx6 (0.392) Nr2c2 (0.392) Zfp524 (0.392) Elf1 (0.392) Sp1 (0.392) Maz (0.483) Egr4 (0.483) Zfp740 (0.483) Egr1 (0.483) Egr1 (0.483) Klf16 (0.483) Zic5 (0.501) Gabpa (0.526) Gcm1 (0.561) Rfx4 (0.597) Klf15 (0.597) Zfp740 (0.597) Cenpb (0.602) Mbd2 (0.625) Sp5 (0.641) Ets1 (0.668) Gcm2 (0.668) Egr2 (0.668) E2f3 (0.668) Zfp281 (0.668) Spdef (0.668) Rfx4 (0.668) | BP: small GTPase mediated signal transduction CC: transcription factor complex MF: ATP binding MF: zinc ion binding MF: magnesium ion binding |
| 26 | 90 |  |  | 0.099 | Nfia (0.099) Ctcfl (0.099) Tcfap2c (0.099) Sp3 (0.112) Zfx (0.112) Max (0.124) E2F3 (0.124) Hey1 (0.124) Nfib (0.124) Tcfap2d (0.124) Plagl1 (0.126) Clock (0.126) Neurod1 (0.162) E2F2 (0.162) Hes5 (0.197) Hes7 (0.197) Max (0.197) Klf5 (0.207) Mbd2 (0.207) Zic5 (0.229) Mycn (0.236) Npas2 (0.236) Egr4 (0.259) Klf16 (0.268) Hes1 (0.285) Klf6 (0.285) | BP: negative regulation of transcription from RNA polymerase II promoter CC: transcription factor complex MF: ATP binding MF: zinc ion binding MF: transcription activator activity |
| 27 | 105 |  |  | 0.099 | Sp3 (0.099) E2f1 (0.099) Sp2 (0.099) Egr3 (0.099) Wt1 (0.099) Tcfap2d (0.099) Nr2f2 (0.104) Egr1 (0.104) Zic4 (0.136) Smad3 (0.136) Zic1 (0.154) Sp1 (0.157) Zic2 (0.157) Zic3 (0.162) Glis3 (0.175) Zic2 (0.175) Klf6 (0.232) Zic3 (0.242) Egr4 (0.267) Max (0.267) Zfp161 (0.267) Zic1 (0.267) Zic5 (0.267) Zfx (0.267) Zbtb7a (0.267) | CC: transcription factor complex MF: ATP binding MF: zinc ion binding MF: transcription activator activity MF: magnesium ion binding |
| 28 | 10 |  |  | 0.114 | Zbtb3 (0.114) Zbtb3 (0.721) Zscan10 (0.721) Zfp161 (0.721) Prdm4 (0.721) Nfib (0.721) Foxh1 (0.839) | BP: G-protein coupled receptor protein signaling pathway BP: cell adhesion CC: extracellular space CC: integral to membrane MF: olfactory receptor activity |
| 29 | 58 |  |  | 0.114 | Tcf3 (0.114) Zscan4 (0.144) Tcf12 (0.144) Id4 (0.192) Ptf1a (0.192) Myog (0.192) Myod1 (0.249) Ascl2 (0.288) Zic3 (0.288) Bhlhe40 (0.288) Myf6 (0.288) Zscan4c (0.288) Zscan4 (0.318) Nhlh2 (0.318) Ferd3l (0.318) Tcfe2a (0.373) Mlxip (0.644) Zic1 (0.678) Hey2 (0.720) Tcfap4 (0.737) Zfp691 (0.737) Myf6 (0.737) Tcf4 (0.737) Npas2 (0.737) Myf6 (0.738) Zfp354c (0.738) | BP: leukotriene biosynthetic process CC: extracellular space MF: serine-type endopeptidase activity MF: calcium ion binding MF: cytokine activity |
| 30 | 30 |  |  | 0.117 | E2f3 (0.117) Mbd2 (0.179) Zic2 (0.238) Ctcfl (0.238) Tcfap2a (0.238) Zic1 (0.238) Atf6 (0.238) Zic3 (0.238) Tcfap2c (0.238) Nr2f2 (0.238) Tcfap2d (0.238) Zfp740 (0.238) Zfp263 (0.238) Ebf1 (0.238) Zfp161 (0.238) Plagl1 (0.238) Zic5 (0.238) Ctcf (0.259) Tcf3 (0.267) Egr4 (0.291) Zfp148 (0.295) Wt1 (0.324) E2f4 (0.353) Zfx (0.353) E2f1 (0.369) Zfp524 (0.369) Tcfap2b (0.369) Sp3 (0.369) Plagl1 (0.369) Sp1 (0.369) Egr2 (0.369) Zfp281 (0.369) | BP: transcription CC: transcription factor complex MF: ATP binding MF: zinc ion binding MF: transcription activator activity |
| 31 | 48 |  |  | 0.121 | E2f1 (0.121) Mbd1 (0.546) Mbtps2 (0.546) Yy1 (0.546) Zfp161 (0.546) Npas4 (0.546) Zbtb7a (0.546) Rxrb (0.546) Nr2c1 (0.546) Esr1 (0.546) E2f3 (0.546) Wt1 (0.546) Smad3 (0.546) Gmeb1 (0.546) Rxrg (0.546) Nr2f1 (0.546) E2F3 (0.624) Rarb (0.632) Plagl1 (0.740) Rara (0.759) Sp1 (0.759) Nr2f2 (0.801) Nr2f6 (0.801) Rxra (0.801) Egr1 (0.801) E2F2 (0.801) | BP: negative regulation of transcription from RNA polymerase II promoter CC: transcription factor complex MF: ATP binding MF: zinc ion binding MF: magnesium ion binding |
| 32 | 1 |  |  | 0.126 | Wt1 (0.126) Sp3 (0.273) Egr1 (0.273) Sp1 (0.341) Nr2f2 (0.341) Zbtb7a (0.386) Max (0.505) Zfp219 (0.505) E2f3 (0.505) Zfp148 (0.505) Egr3 (0.505) Klf6 (0.505) Klf5 (0.505) Bcl6b (0.505) Sp4 (0.505) Zbtb7c (0.505) Egr2 (0.505) Klf7 (0.505) Mbd2 (0.505) Rara (0.507) Glis2 (0.507) Plagl1 (0.507) Zfp281 (0.513) Klf2 (0.532) Klf4 (0.532) Plagl1 (0.532) Egr1 (0.532) Klf16 (0.532) Klf15 (0.532) | BP: transcription MF: ATP binding MF: zinc ion binding MF: transcription activator activity MF: magnesium ion binding |
| 33 | 91 |  |  | 0.127 | Mbd2 (0.127) Egr4 (0.127) Wt1 (0.127) Sp3 (0.232) Zfx (0.233) Plagl1 (0.275) Sp2 (0.275) Tcfap2d (0.275) E2f1 (0.275) Zbtb7a (0.275) Gm98 (0.275) Zic5 (0.275) Ctcfl (0.316) Klf16 (0.316) E2f3 (0.316) Tcf3 (0.318) Plagl1 (0.318) Sp1 (0.321) Smad3 (0.321) Nr2f2 (0.441) Zfp219 (0.501) Klf6 (0.501) E2f4 (0.502) Smad1 (0.571) Prdm4 (0.576) Egr1 (0.576) | BP: transcription CC: transcription factor complex MF: ATP binding MF: zinc ion binding MF: magnesium ion binding |
| 34 | 117 |  |  | 0.141 | Tcfap2b (0.141) Irf6 (0.141) Tcfap2a (0.141) Tcfap2c (0.141) Sohlh2 (0.163) Tcfap2c (0.163) Tcfap2a (0.163) Ctcfl (0.163) Max (0.235) Egr4 (0.304) Tcfap2e (0.462) Zfx (0.482) Nr2f2 (0.482) Zfp161 (0.482) Pax5 (0.482) E2f1 (0.482) Tcfap2d (0.482) Klf5 (0.482) Elf1 (0.486) Wt1 (0.486) Mbd2 (0.494) Tcfap2b (0.552) Egr1 (0.552) E2f3 (0.552) Trp63 (0.552) Zic1 (0.552) Sp1 (0.552) | BP: transcription CC: transcription factor complex MF: ATP binding MF: zinc ion binding MF: transcription activator activity |
| 35 | 33 |  |  | 0.157 | E2f1 (0.157) Zfp161 (0.157) Zfp161 (0.157) Plagl1 (0.157) Klf16 (0.157) Hes7 (0.157) Hes1 (0.157) E2F2 (0.157) E2F3 (0.157) Tcfl5 (0.157) Zbtb7a (0.157) Sp3 (0.157) Hey1 (0.157) Tcfap2d (0.182) Max (0.182) Bcl6b (0.182) Ctcfl (0.182) Hes5 (0.182) Egr4 (0.183) Sp1 (0.183) Wt1 (0.183) Klf7 (0.183) Klf6 (0.193) Sp5 (0.216) Sp8 (0.229) | BP: negative regulation of transcription from RNA polymerase II promoter MF: transcription factor activity MF: sequence-specific DNA binding MF: ATP binding MF: zinc ion binding |
| 36 | 77 |  |  | 0.159 | Myog (0.159) Jundm2 (0.159) Tcf12 (0.301) Atf1 (0.301) Egr4 (0.301) Zfx (0.301) Creb3 (0.324) Plagl1 (0.324) Plag1 (0.324) Gm98 (0.324) Nhlh1 (0.324) Ctcfl (0.324) Mafa (0.324) Sp2 (0.469) Jdp2 (0.469) Zbtb7a (0.469) Myod1 (0.469) Fosl2 (0.469) Batf3 (0.481) Sp3 (0.519) Atf3 (0.526) Gata6 (0.526) Tcfap2d (0.526) Tcfap2c (0.526) Tcfap2a (0.583) | BP: positive regulation of transcription from RNA polymerase II promoter BP: Wnt receptor signaling pathway BP: negative regulation of transcription from RNA polymerase II promoter MF: ATP binding MF: zinc ion binding |
| 37 | 110 |  |  | 0.193 | Egr1 (0.193) Zic5 (0.193) Zic1 (0.193) Tcfap2d (0.193) Tcfap2c (0.193) Zfp740 (0.193) Rara (0.282) Zic4 (0.508) Zic3 (0.528) Tcfap2b (0.528) Zic2 (0.528) Insm1 (0.528) Twist1 (0.575) Glis3 (0.575) Zfx (0.575) Nr2c2 (0.710) Tcfap2c (0.725) Tcfap2a (0.730) Myf6 (0.766) Zfp219 (0.773) Spdef (0.773) Tcfap2a (0.773) Zfp740 (0.773) Tcfap2e (0.773) Clock (0.773) Hes1 (0.773) Creb3l2 (0.773) E2f3 (0.773) Zbtb7a (0.773) Gcm1 (0.773) Nr2f2 (0.773) Sohlh2 (0.773) Bach2 (0.773) Tcf4 (0.773) Ctcfl (0.773) Smad3 (0.773) Plagl1 (0.773) Ebf1 (0.773) Mlxipl (0.773) Usf2 (0.773) E2f5 (0.773) Wt1 (0.773) Sp2 (0.773) Sp4 (0.773) Hes7 (0.773) Hes5 (0.773) Tcfap2b (0.773) | BP: transcription CC: transcription factor complex MF: zinc ion binding MF: ATP binding MF: transcription activator activity |
| 38 | 74 |  |  | 0.201 | Gsc2 (0.201) Gsc (0.201) Pitx3 (0.201) Pitx3 (0.201) Pitx2 (0.379) Otx2 (0.379) Obox5 (0.379) Pitx1 (0.379) Gm4830 (0.379) Otx1 (0.402) Dmbx1 (0.402) Gsc (0.443) Crx (0.443) Tcf1 (0.551) AC189028.1 (0.551) Dnajc21 (0.883) | BP: RNA splicing CC: photoreceptor connecting cilium CC: spliceosomal complex MF: G-protein beta/gamma-subunit binding MF: NADH dehydrogenase (ubiquinone) activity |
| 39 | 127 |  |  | 0.238 | Gli2 (0.238) Glis2 (0.238) Plagl1 (0.238) Gli3 (0.238) Sp1 (0.251) Glis2 (0.251) Sp4 (0.251) Egr3 (0.251) Klf6 (0.251) Glis3 (0.281) Sp3 (0.281) Zic4 (0.281) Klf16 (0.281) Irf6 (0.281) Gli1 (0.305) Glis1 (0.310) Wt1 (0.310) Zbtb7a (0.310) Sp8 (0.322) Ybx1 (0.362) Plagl1 (0.378) Eomes (0.399) Relb (0.399) Max (0.423) Zic5 (0.458) | BP: transcription BP: negative regulation of transcription from RNA polymerase II promoter CC: transcription factor complex MF: ATP binding MF: zinc ion binding |
| 40 | 88 |  |  | 0.258 | Irf3 (0.258) Nr4a2 (0.391) Nr1i2 (0.596) Pparg (0.855) Ppara (0.855) Myf6 (0.892) Esrra (0.892) Nr1i3 (0.892) Nr2f6 (0.892) Mafk (0.892) Ppard (0.892) | BP: sensory perception of smell BP: G-protein coupled receptor protein signaling pathway BP: defense response CC: extracellular region MF: olfactory receptor activity |
| 41 | 101 |  |  | 0.267 | Neurod1 (0.267) Zfp187 (0.267) Mtf1 (0.267) Nfib (0.267) Tcfap2c (0.304) Hey2 (0.512) Myc (0.512) Nr5a1 (0.512) Tlx1 (0.512) Atoh1 (0.512) Arntl (0.512) Twist2 (0.512) Tal1 (0.512) Mycn (0.512) Clock (0.512) Bhlhe22 (0.512) Nfia (0.551) Zscan4 (0.551) Creb3l2 (0.561) Id4 (0.561) Neurod2 (0.561) Id2 (0.585) Tcf15 (0.585) Nr5a2 (0.585) Rest (0.585) | BP: defense response to bacterium CC: extracellular space CC: integral to membrane MF: serine-type endopeptidase activity MF: calcium ion binding |
| 42 | 83 |  |  | 0.283 | Tcfap2d (0.283) Smad1 (0.307) Zfx (0.307) Tcfap2a (0.307) Egr4 (0.307) Tcfap2a (0.307) Tcfap2c (0.310) Neurod1 (0.353) Tcfap2c (0.353) Zbtb7a (0.518) Nfib (0.631) Tcfap2b (0.670) Gm397 (0.670) Ppard (0.782) Zfp187 (0.782) Tcfap2b (0.804) Clock (0.804) Klf16 (0.863) Zfp281 (0.863) Esrra (0.863) Irf6 (0.863) Sp3 (0.863) Zfp148 (0.863) | BP: transcription BP: positive regulation of transcription from RNA polymerase II promoter BP: negative regulation of transcription from RNA polymerase II promoter BP: morphogenesis of an epithelium CC: transcription factor complex |
| 43 | 78 |  |  | 0.300 | Zscan20 (0.300) Tcfcp2l1 (0.300) | BP: response to external stimulus BP: defense response to bacterium BP: innate immune response BP: icosanoid metabolic process CC: extracellular space |
| 44 | 43 |  |  | 0.303 | Zfp161 (0.303) Zfp740 (0.495) Elf1 (0.779) Klf6 (0.779) Stat3 (0.892) Zfp219 (0.892) Smad3 (0.892) E2f1 (0.892) Egr4 (0.892) Sohlh2 (0.892) Zic4 (0.892) Irf6 (0.892) Thra (0.892) Ctcfl (0.892) Zbtb3 (0.892) Tcfap2a (0.892) Mbd2 (0.892) Zic1 (0.892) Tcfap2c (0.892) Sp4 (0.892) Zfp410 (0.892) Gabpa (0.892) Sp5 (0.892) Zic5 (0.892) Elf5 (0.892) Zfp143 (0.892) Zic2 (0.892) Egr3 (0.892) Ehf (0.892) Tcfap2e (0.892) Tcf1 (0.892) Zbtb3 (0.892) Mtf1 (0.892) Figla (0.892) Sp3 (0.892) Ets2 (0.892) Foxi1 (0.892) Insm1 (0.892) Egr1 (0.892) Zic3 (0.892) Ctcf (0.892) Zic3 (0.892) Gli3 (0.892) Gli1 (0.892) Eomes (0.892) Tcfap2a (0.892) Zic1 (0.892) Tcfap2c (0.892) Stat1 (0.892) Hic2 (0.892) Sp4 (0.892) Gcm2 (0.892) | CC: transcription factor complex MF: ATP binding MF: zinc ion binding MF: magnesium ion binding MF: manganese ion binding |
| 45 | 107 |  |  | 0.304 | Plagl1 (0.304) Plagl1 (0.304) Zic5 (0.338) Zfp161 (0.338) Sp2 (0.338) Gmeb1 (0.338) Zic4 (0.368) Glis2 (0.372) Yy1 (0.372) Klf6 (0.372) E2f1 (0.379) Gli3 (0.379) E2f3 (0.379) Ctcfl (0.379) Sp1 (0.379) Mlxipl (0.379) Glis3 (0.379) Mbtps2 (0.385) Gli2 (0.496) E2F3 (0.538) Plag1 (0.552) Egr3 (0.561) Zbtb7a (0.670) Ctcf (0.673) Mtf1 (0.673) | BP: protein amino acid phosphorylation BP: negative regulation of transcription from RNA polymerase II promoter MF: ATP binding MF: zinc ion binding MF: transcription activator activity |
| 46 | 124 |  |  | 0.315 | Tcfap2d (0.315) | BP: cell division BP: negative regulation of transcription from RNA polymerase II promoter MF: transcription factor activity MF: ATP binding MF: zinc ion binding |
| 47 | 119 |  |  | 0.317 | Zfx (0.317) Zic5 (0.317) Tcfap2d (0.317) Sp3 (0.817) Wt1 (0.817) | BP: protein amino acid phosphorylation CC: transcription factor complex MF: ATP binding MF: zinc ion binding MF: transcription activator activity |
| 48 | 85 |  |  | 0.321 | Nhlh2 (0.321) Tcfe2a (0.321) Gata1 (0.498) | BP: G-protein coupled receptor protein signaling pathway BP: sensory perception of smell BP: cell communication MF: olfactory receptor activity MF: calcium ion binding |
| 49 | 79 |  |  | 0.323 | Nr2c2 (0.323) Zfx (0.323) E2f3 (0.323) Klf6 (0.323) Nr2f2 (0.323) Gabpa (0.323) Zic1 (0.323) Sp3 (0.323) Egr1 (0.323) Zfp161 (0.342) E2f1 (0.342) Zfp161 (0.342) Tcfap2d (0.342) Ctcfl (0.342) Spdef (0.342) Mbd2 (0.342) Sp5 (0.342) Zic2 (0.342) Zic3 (0.342) Tcfap2a (0.342) Bcl6b (0.342) Ascl2 (0.342) Myf6 (0.361) Mtf1 (0.373) Mtf1 (0.373) Nhlh1 (0.373) Wt1 (0.373) | CC: transcription factor complex MF: ATP binding MF: zinc ion binding MF: magnesium ion binding MF: transcription activator activity |
| 50 | 70 |  |  | 0.362 | Tcfap2d (0.362) Wt1 (0.362) Nr2f2 (0.430) Sp3 (0.430) Tcf3 (0.430) Sp1 (0.432) Prdm4 (0.432) E2f1 (0.432) Zbtb7a (0.432) Hes7 (0.432) Mbd2 (0.432) Neurod1 (0.432) Klf6 (0.432) Ctcfl (0.444) Klf16 (0.482) Zfx (0.482) Hes1 (0.513) Egr4 (0.519) Plagl1 (0.578) Tcf4 (0.714) Rest (0.723) Sp4 (0.723) Sp2 (0.723) Zfp161 (0.723) Zfp263 (0.723) Gmeb1 (0.723) Gm98 (0.723) Tcfl5 (0.723) Smad3 (0.723) | BP: transcription CC: transcription factor complex MF: ATP binding MF: zinc ion binding MF: magnesium ion binding |
| 51 | 100 |  |  | 0.370 | Rest (0.370) | BP: response to external stimulus CC: cell junction MF: calcium ion binding MF: transcription factor activity MF: cation channel activity |
| 52 | 24 |  |  | 0.403 | Plag1 (0.403) Max (0.403) Clock (0.403) Mycn (0.501) Tcfe3 (0.501) Creb3l2 (0.501) Mitf (0.501) Ctcfl (0.501) Myc (0.501) Rest (0.711) Ctcf (0.711) Tcfap2b (0.711) Tcfap2c (0.711) Npas2 (0.717) Runx2 (0.853) Zfx (0.853) Tcfap2a (0.853) Zic1 (0.853) Tcfe2a (0.858) Max (0.865) Mlxip (0.865) Hey1 (0.865) Nr4a3 (0.865) Runx1 (0.865) Hes5 (0.866) Klf6 (0.866) Klf16 (0.866) Thra (0.866) Usf2 (0.866) | BP: anatomical structure formation involved in morphogenesis CC: plasma membrane CC: proteinaceous extracellular matrix MF: transcription factor activity MF: sequence-specific DNA binding |
| 53 | 111 |  |  | 0.408 | E2f1 (0.408) Mbd1 (0.875) | BP: transcription BP: protein amino acid phosphorylation BP: negative regulation of transcription from RNA polymerase II promoter MF: ATP binding MF: zinc ion binding |
| 54 | 87 |  |  | 0.429 | Egr4 (0.429) Zfx (0.429) Atf6 (0.429) Zic3 (0.429) Egr3 (0.429) Zic1 (0.429) Tcfap2c (0.447) Wt1 (0.447) Arid2 (0.481) Egr2 (0.593) E2F2 (0.593) Zic1 (0.593) Zic3 (0.593) Egr1 (0.593) E2f4 (0.593) Zic2 (0.654) Klf3 (0.748) Zic2 (0.748) Rfx2 (0.748) Sp5 (0.782) E2F3 (0.826) Rfx6 (0.833) E2f1 (0.833) Egr1 (0.833) Nr2c2 (0.833) Sp3 (0.833) Klf5 (0.833) Tcfap2b (0.833) Rfx4 (0.833) Tlx1 (0.833) Mlxipl (0.833) Ctcfl (0.833) Tcfap2d (0.833) Zeb1 (0.833) Nhlh2 (0.833) Mbd2 (0.833) Rfx1 (0.833) Zfp161 (0.833) Cxxc1 (0.833) Zic4 (0.833) Plagl1 (0.833) Tcfap2e (0.833) Bach1 (0.833) Klf6 (0.833) E2f6 (0.833) | BP: transcription BP: positive regulation of transcription from RNA polymerase II promoter BP: negative regulation of transcription from RNA polymerase II promoter MF: ATP binding MF: zinc ion binding |
| 55 | 51 |  |  | 0.435 | Prdm4 (0.435) Pknox2 (0.435) Tgif1 (0.435) Snai1 (0.435) Tgif2 (0.450) Meis1 (0.508) Mrg2 (0.508) Meis2 (0.508) Zfp369 (0.508) Meis3 (0.556) Pknox1 (0.556) Tgif2 (0.595) Mrg1 (0.642) Pknox1 (0.726) Zkscan4 (0.726) Nr5a1 (0.780) Tbx2 (0.780) Zfp691 (0.789) Hnf4a (0.789) Tgif1 (0.789) Pbx1 (0.882) Zbtb7b (0.882) | BP: sensory perception of smell BP: G-protein coupled receptor protein signaling pathway BP: signal transduction CC: integral to membrane MF: olfactory receptor activity |
| 56 | 82 |  |  | 0.439 | Egr4 (0.439) Mbd2 (0.439) E2f1 (0.511) Ctcfl (0.511) Zfx (0.511) Zic2 (0.511) Sp3 (0.511) Zbtb49 (0.511) Zic1 (0.511) Zic5 (0.511) E2f3 (0.511) Zic3 (0.511) Sp2 (0.748) Tcfap2d (0.748) Sohlh2 (0.748) Smad1 (0.748) Myf6 (0.748) Bcl6b (0.768) Smarcc2 (0.768) E2f4 (0.768) Pbx3 (0.768) Gabpa (0.768) Plagl1 (0.768) Wt1 (0.787) Egr3 (0.859) | BP: negative regulation of transcription from RNA polymerase II promoter CC: transcription factor complex MF: ATP binding MF: zinc ion binding MF: manganese ion binding |
| 57 | 27 |  |  | 0.446 | Tcf7l1 (0.446) | BP: immune response BP: inflammatory response BP: defense response to bacterium CC: integral to membrane MF: serine-type endopeptidase activity |
| 58 | 7 |  |  | 0.459 | Tcfap2c (0.459) Nfia (0.459) Sox14 (0.459) Nfib (0.459) Osr1 (0.459) Zic2 (0.459) Zic1 (0.459) Zic3 (0.459) Tcfap2c (0.459) Tcfap2a (0.503) Yy1 (0.544) Sp2 (0.544) Osr2 (0.544) Zfp740 (0.544) Tcfap2d (0.544) Ctcf (0.544) Twist1 (0.544) Gcm1 (0.544) Tcfap2b (0.544) Tcfap2a (0.544) Max (0.544) Tcfap2b (0.544) Nr2c2 (0.544) Myf6 (0.544) Tcfap2c (0.544) Tcfe2a (0.544) Smad3 (0.544) Gabpa (0.544) | BP: transcription BP: protein amino acid phosphorylation BP: negative regulation of transcription from RNA polymerase II promoter MF: potassium ion binding MF: calcium ion binding |
| 59 | 19 |  |  | 0.468 | Esrra (0.468) Rest (0.468) | BP: sensory perception of smell BP: G-protein coupled receptor protein signaling pathway BP: signal transduction CC: integral to membrane MF: olfactory receptor activity |
| 60 | 6 |  |  | 0.494 | Mbd1 (0.494) Zfp161 (0.539) Nfya (0.539) Tcfl5 (0.809) Nr2f2 (0.809) Ahr (0.809) Arnt2 (0.809) E2F2 (0.809) E2f1 (0.853) Tcf1 (0.874) Srebf2 (0.874) | CC: transcription factor complex MF: transcription factor activity MF: ATP binding MF: zinc ion binding MF: transcription activator activity |
| 61 | 47 |  |  | 0.503 | Nfat5 (0.503) Nfatc1 (0.899) | BP: G-protein coupled receptor protein signaling pathway BP: sensory perception of smell BP: signal transduction BP: cell communication MF: olfactory receptor activity |
| 62 | 28 |  |  | 0.514 | Lin54 (0.514) | BP: sensory perception of smell BP: G-protein coupled receptor protein signaling pathway BP: signal transduction BP: cell communication MF: olfactory receptor activity |
| 63 | 84 |  |  | 0.519 | Mbd2 (0.519) Smad3 (0.558) | BP: mRNA processing BP: RNA splicing CC: transcription factor complex MF: ATP binding MF: zinc ion binding |
| 64 | 114 |  |  | 0.545 | Klf3 (0.545) Zic2 (0.545) Zic3 (0.545) Ctcfl (0.545) Sox14 (0.545) Zic5 (0.545) Plagl1 (0.545) Zic1 (0.545) Twist1 (0.545) Gli1 (0.545) Esr2 (0.545) Zfp369 (0.545) Gli3 (0.611) Ctcf (0.611) Tcfap2b (0.611) Zkscan4 (0.634) Insm1 (0.674) Tcf4 (0.705) Zfp740 (0.705) Hbp1 (0.705) Zic2 (0.705) Plagl1 (0.731) Tcfap2a (0.765) | BP: transcription BP: potassium ion transport MF: transcription factor activity MF: sequence-specific DNA binding MF: potassium ion binding |
| 65 | 80 |  |  | 0.573 | Ets2 (0.573) E2f1 (0.573) Egr4 (0.573) E2f4 (0.573) Zfp219 (0.573) Zfp161 (0.658) Zfp263 (0.684) Elf1 (0.684) E2f6 (0.735) Sp3 (0.735) Maz (0.735) | BP: negative regulation of transcription from RNA polymerase II promoter CC: transcription factor complex MF: ATP binding MF: zinc ion binding MF: translation regulator activity |
| 66 | 93 |  |  | 0.590 | Creb3 (0.590) Atf3 (0.590) Batf3 (0.590) | BP: G-protein coupled receptor protein signaling pathway CC: integral to membrane CC: plasma membrane CC: nucleosome MF: serine-type endopeptidase activity |
| 67 | 31 |  |  | 0.676 | Rest (0.676) Trp53 (0.676) | CC: plasma membrane CC: proteinaceous extracellular matrix MF: transcription factor activity MF: calcium ion binding MF: sequence-specific DNA binding |
| 68 | 57 |  |  | 0.728 | Ppard (0.728) Nr1h2 (0.728) Npas2 (0.728) Hes7 (0.728) Nr4a3 (0.728) Hes1 (0.728) Clock (0.728) Hey2 (0.728) Hes5 (0.728) Tcfap2a (0.728) Ferd3l (0.728) Rxra (0.887) Pparg (0.887) Tcfap2c (0.887) Hnf4g (0.887) | BP: response to external stimulus CC: proteinaceous extracellular matrix CC: plasma membrane part MF: calcium ion binding MF: serine-type endopeptidase activity |
| 69 | 73 |  |  | 0.764 | Gmeb2 (0.764) Sp100 (0.764) | BP: cell division CC: transcription factor complex MF: ATP binding MF: zinc ion binding MF: transcription activator activity |
| 70 | 120 |  |  | 0.810 | Nfia (0.810) Zscan4 (0.810) | BP: G-protein coupled receptor protein signaling pathway BP: defense response to bacterium BP: chemotaxis BP: inflammatory response MF: serine-type endopeptidase activity |
