# Supplementary material for: DeepCpG: accurate prediction of single-cell DNA methylation states using deep learning
Source: Genome Biol. 2017 Apr 11;18:67. doi: 10.1186/s13059-017-1189-z (PMC5387360; doi:10.1186/s13059-017-1189-z)
Supplement: Supplementary file 3 — Sequence motifs. HTML files with sequence logos and summary statistics for all cell types. (ZIP 19482 kb) [file 13059_2017_1189_MOESM3_ESM.zip › serum.html]

Filter table


# Filter table

#### *Christof Angermueller*

#### *2017-01-13*

Nr | Label | Logo | Influence | q-value | Related known motifs | GO annotations || 1 | 30: Ctcf |  |  | 0.000 | Ctcf (0.000) Twist1 (0.013) Ctcfl (0.017) Neurod1 (0.017) Atoh8 (0.053) Scrt1 (0.053) Snai2 (0.056) Scrt2 (0.056) Neurod2 (0.056) Atoh1 (0.056) Neurog1 (0.056) Max (0.085) Msc (0.113) Tcfap4 (0.135) Yy1 (0.135) Myc (0.143) Twist2 (0.143) Bhlhe22 (0.143) Mycn (0.158) Tcf4 (0.158) Tcf12 (0.158) Tal1 (0.158) Myog (0.158) Tcf3 (0.158) Olig2 (0.174) |  |
| 2 | 33: Sp3 |  |  | 0.000 | Sp3 (0.000) Klf7 (0.013) E2f3 (0.013) Klf2 (0.013) Zbtb7a (0.015) Klf5 (0.015) E2f1 (0.016) Wt1 (0.016) Klf4 (0.016) Egr4 (0.017) Zfx (0.017) Klf7 (0.017) Zfp219 (0.020) Zfp281 (0.020) Egr1 (0.020) Klf12 (0.020) Klf16 (0.026) Egr1 (0.026) Plagl1 (0.027) Zfp740 (0.033) Egr2 (0.033) Klf8 (0.033) Zfp148 (0.034) Sp5 (0.034) Tcfap2d (0.034) | BP: transcription CC: transcription factor complex MF: ATP binding MF: zinc ion binding MF: magnesium ion binding |
| 3 | 79: Klf16 |  |  | 0.000 | Klf16 (0.000) Klf5 (0.001) Egr4 (0.001) Klf4 (0.001) Sp5 (0.001) Klf14 (0.001) Klf7 (0.001) Sp8 (0.001) Sp3 (0.001) Klf8 (0.001) Klf2 (0.002) Klf12 (0.002) Wt1 (0.002) Sp4 (0.003) Sp1 (0.003) Klf7 (0.004) Klf13 (0.007) Klf1 (0.009) Zfp148 (0.015) Clock (0.019) Zfp281 (0.019) Max (0.021) Sp4 (0.024) Zbtb7a (0.024) Klf6 (0.035) | BP: transcription BP: protein amino acid phosphorylation MF: transcription factor activity MF: sequence-specific DNA binding MF: ATP binding |
| 4 | 117: Klf7 |  |  | 0.000 | Klf7 (0.000) Sp8 (0.000) Sp5 (0.000) Sp1 (0.000) Klf14 (0.000) Sp3 (0.000) Klf5 (0.000) Klf16 (0.000) Sp4 (0.000) Klf7 (0.000) Sp4 (0.000) Klf8 (0.001) Klf4 (0.001) Klf12 (0.001) Zfp281 (0.001) Zfp148 (0.001) Zfp740 (0.001) Wt1 (0.001) Klf13 (0.001) Klf2 (0.002) Egr2 (0.002) Zfp410 (0.003) Bcl6b (0.004) Egr4 (0.004) Maz (0.004) Zfp281 (0.004) Klf6 (0.004) | BP: negative regulation of transcription from RNA polymerase II promoter BP: small GTPase mediated signal transduction CC: transcription factor complex CC: dendrite MF: zinc ion binding |
| 5 | 121: Ctcf |  |  | 0.000 | Ctcf (0.000) Ctcfl (0.002) Neurod1 (0.004) Zic4 (0.092) Twist1 (0.092) Zic1 (0.092) Atoh8 (0.092) Zic2 (0.092) Atoh1 (0.094) Zic3 (0.094) Scrt1 (0.094) Scrt2 (0.094) Neurod2 (0.094) Myc (0.095) Sp4 (0.100) Yy1 (0.117) Tcf3 (0.117) Tal1 (0.176) Klf13 (0.176) Neurog1 (0.176) Max (0.176) Bhlhe22 (0.176) Zic5 (0.176) Mbtps2 (0.176) Msc (0.176) | BP: peptidyl-amino acid modification BP: maternal placenta development CC: plasma membrane CC: photoreceptor inner segment CC: extracellular space |
| 6 | 27: Foxa2 |  |  | 0.001 | Foxa2 (0.001) Foxl1 (0.214) Foxk1 (0.714) Zfp105 (0.714) Arid3b (0.714) Foxd3 (0.714) Elf3 (0.714) Tcf3 (0.714) Hmg20b (0.714) Irx1 (0.714) Srf (0.714) Sox7 (0.714) Mef2c (0.714) Mtf1 (0.714) Sox18 (0.714) Mef2a (0.714) Onecut3 (0.714) Glis2 (0.714) Irx4 (0.714) Irx5 (0.714) Gm5294 (0.714) Sox21 (0.714) Hmga1 (0.714) Foxj1 (0.724) Mecom (0.724) Onecut1 (0.724) | BP: sensory perception of smell BP: G-protein coupled receptor protein signaling pathway BP: signal transduction MF: olfactory receptor activity MF: RNA binding |
| 7 | 53: Sp1 |  |  | 0.002 | Sp1 (0.002) Klf6 (0.011) Bcl6b (0.011) E2f3 (0.011) Zfp410 (0.011) Sp4 (0.018) Zfp148 (0.022) Klf5 (0.022) Klf16 (0.022) Zfp281 (0.022) Zfp740 (0.026) Wt1 (0.044) Sp5 (0.054) Klf7 (0.064) Klf7 (0.064) Sp8 (0.080) Klf8 (0.080) Klf4 (0.080) Egr4 (0.080) Zfp740 (0.080) Klf12 (0.080) E2f6 (0.080) E2f4 (0.082) Cenpb (0.091) Zfp281 (0.091) Sp4 (0.091) Sp3 (0.091) | BP: transcription BP: protein amino acid phosphorylation CC: transcription factor complex MF: zinc ion binding MF: ATP binding |
| 8 | 1: Zic1 |  |  | 0.003 | Zic1 (0.003) Zic2 (0.003) Sp3 (0.003) Zic3 (0.003) E2f3 (0.004) Wt1 (0.006) Tcfap2d (0.018) Zfx (0.021) Ctcfl (0.023) Egr1 (0.029) Tcfap2c (0.029) Zic4 (0.029) E2f1 (0.029) Egr4 (0.029) Zic2 (0.030) Tcfap2a (0.032) Sp1 (0.032) Mbd2 (0.041) Nr2f2 (0.053) Zbtb7a (0.053) Zfp281 (0.062) E2f4 (0.064) Egr2 (0.080) Egr3 (0.084) Klf6 (0.087) Zic5 (0.087) | CC: transcription factor complex MF: ATP binding MF: zinc ion binding MF: magnesium ion binding MF: manganese ion binding |
| 9 | 45: Klf6 |  |  | 0.003 | Klf6 (0.003) Sp3 (0.035) Wt1 (0.104) Zbtb7a (0.104) Zfp219 (0.104) Sp1 (0.156) Zbtb7b (0.156) Mafa (0.157) Egr4 (0.175) Maz (0.175) Klf16 (0.175) Nhlh1 (0.175) Zfp148 (0.175) Zfp281 (0.203) Klf5 (0.204) Myod1 (0.206) Smad3 (0.209) Myog (0.209) Zfp740 (0.209) E2f3 (0.209) Egr1 (0.209) Sp5 (0.209) Sp4 (0.243) Plagl1 (0.243) Mbd2 (0.243) | BP: transcription BP: negative regulation of transcription from RNA polymerase II promoter CC: transcription factor complex MF: ATP binding MF: zinc ion binding |
| 10 | 67: Sp4 |  |  | 0.003 | Sp4 (0.003) Sp1 (0.007) Klf14 (0.007) Maz (0.007) Sp4 (0.007) Sp3 (0.008) Zfp148 (0.011) Klf5 (0.011) Zfp281 (0.011) Klf7 (0.011) Klf6 (0.011) Wt1 (0.012) Sp8 (0.012) Klf13 (0.018) Sp5 (0.019) Klf16 (0.020) Egr1 (0.020) Zfp219 (0.039) Ascl2 (0.040) Klf7 (0.056) Sfpi1 (0.056) Klf8 (0.056) Klf4 (0.058) Zfp740 (0.064) Bcl11a (0.066) E2f1 (0.066) | BP: transcription BP: negative regulation of transcription from RNA polymerase II promoter BP: inner ear morphogenesis CC: transcription factor complex MF: transcription activator activity |
| 11 | 46: Hmg20b |  |  | 0.004 | Hmg20b (0.004) Sry (0.004) Sox21 (0.017) Sox14 (0.017) Tbp (0.697) Sox30 (0.766) Cdc5l (0.766) Tcf7 (0.800) Foxl1 (0.800) | BP: G-protein coupled receptor protein signaling pathway BP: sensory perception of smell BP: signal transduction CC: integral to membrane MF: olfactory receptor activity |
| 12 | 9: Klf5 |  |  | 0.007 | Klf5 (0.007) Klf4 (0.007) Sp5 (0.007) Klf7 (0.007) Klf16 (0.007) Klf12 (0.007) Klf7 (0.008) Sp8 (0.009) Klf8 (0.009) Sp3 (0.011) Klf14 (0.011) Klf2 (0.011) Klf13 (0.037) Sp1 (0.053) Egr4 (0.076) Wt1 (0.093) Zfp161 (0.093) Egr2 (0.093) Sp4 (0.093) Klf1 (0.098) Smad3 (0.104) Bcl6b (0.113) E2F3 (0.159) Gmeb1 (0.178) Zfx (0.183) Hes7 (0.183) | BP: transcription MF: transcription factor activity MF: ATP binding MF: magnesium ion binding MF: zinc ion binding |
| 13 | 43: Klf2 |  |  | 0.007 | Klf2 (0.007) Klf4 (0.013) Klf5 (0.013) Klf1 (0.013) Klf7 (0.016) Klf8 (0.016) Klf12 (0.016) Klf3 (0.031) Sp5 (0.031) Ascl2 (0.120) Klf7 (0.132) Klf16 (0.132) Sp4 (0.159) Sp4 (0.167) Klf14 (0.224) Sp8 (0.282) Sp1 (0.292) Sp4 (0.300) Egr4 (0.341) Zfp740 (0.341) Arid3a (0.351) Bcl6b (0.370) Mga (0.490) Egr1 (0.493) Tbx15 (0.507) Egr2 (0.507) Sp3 (0.507) | BP: protein amino acid phosphorylation MF: transcription factor activity MF: ATP binding MF: RNA binding MF: zinc ion binding |
| 14 | 126: Zfp161 |  |  | 0.015 | Zfp161 (0.015) E2f1 (0.035) Wt1 (0.048) E2f3 (0.048) E2F3 (0.048) Zfp161 (0.069) E2F2 (0.069) Tcfap2d (0.084) Egr1 (0.087) Sp1 (0.087) Plagl1 (0.087) Egr2 (0.107) Hes7 (0.117) Klf6 (0.120) Tcfl5 (0.120) Hif3a (0.127) Sp2 (0.129) Hes1 (0.137) Zfx (0.137) Egr3 (0.140) Sp3 (0.140) Mbd1 (0.142) Ctcfl (0.149) Mbd2 (0.149) Egr1 (0.199) Sp4 (0.199) Zfp263 (0.199) Nr2f2 (0.199) | CC: transcription factor complex MF: ATP binding MF: zinc ion binding MF: magnesium ion binding MF: transcription activator activity |
| 15 | 15: Zfp740 |  |  | 0.017 | Zfp740 (0.017) Zfp740 (0.021) Klf4 (0.025) Zfp281 (0.032) Sp8 (0.032) Klf8 (0.032) Klf2 (0.032) Sp5 (0.032) Klf5 (0.032) Klf12 (0.032) Zfp202 (0.032) Klf14 (0.032) Zfp148 (0.032) Klf1 (0.032) Klf15 (0.032) Klf7 (0.032) Zfp281 (0.046) Klf7 (0.053) Egr2 (0.062) Ascl2 (0.062) Rreb1 (0.062) Sp1 (0.062) Hif3a (0.062) Sp3 (0.062) Maz (0.087) | BP: transcription BP: anterior/posterior pattern formation BP: negative regulation of transcription from RNA polymerase II promoter BP: inner ear morphogenesis CC: transcription factor complex |
| 16 | 62: Zfx |  |  | 0.019 | Zfx (0.019) Tcfap2d (0.063) Sp3 (0.070) E2f1 (0.075) Nr2f2 (0.075) Plagl1 (0.075) E2F3 (0.091) Egr4 (0.098) Bcl6b (0.121) Mbd2 (0.121) E2f3 (0.124) Sp2 (0.135) Nfia (0.148) Wt1 (0.188) Klf6 (0.188) Klf5 (0.188) Ctcfl (0.222) Tcfap2c (0.251) Smad3 (0.255) Nhlh1 (0.296) Egr1 (0.296) Zfp161 (0.296) E2F2 (0.306) Myf6 (0.306) Egr1 (0.313) | BP: transcription BP: negative regulation of transcription from RNA polymerase II promoter CC: transcription factor complex MF: ATP binding MF: zinc ion binding |
| 17 | 36: Srf |  |  | 0.036 | Srf (0.036) Elf3 (0.445) | BP: sensory perception of smell CC: spliceosomal complex CC: replication fork MF: olfactory receptor activity MF: RNA binding |
| 18 | 118: Egr4 |  |  | 0.043 | Egr4 (0.043) | BP: transcription BP: protein amino acid phosphorylation MF: ATP binding MF: zinc ion binding MF: transcription activator activity |
| 19 | 60: Zfp637 |  |  | 0.047 | Zfp637 (0.047) Irx2 (0.165) Irx4 (0.165) Irx6 (0.165) Irx3 (0.165) Irx5 (0.165) Dmrtc2 (0.171) Irx3 (0.237) Tbp (0.352) Zfp128 (0.517) Tbp (0.605) Hmg20b (0.605) Six6 (0.713) Gm5294 (0.713) | BP: G-protein coupled receptor protein signaling pathway BP: sensory perception of smell BP: signal transduction BP: cell communication MF: olfactory receptor activity |
| 20 | 89: Egr2 |  |  | 0.048 | Egr2 (0.048) Klf1 (0.048) Sp4 (0.048) Sp3 (0.050) Klf2 (0.051) Zfp148 (0.051) Zfp281 (0.051) Klf16 (0.051) Wt1 (0.051) Ctcfl (0.051) Egr3 (0.051) Klf5 (0.051) Klf7 (0.051) Klf8 (0.051) Klf4 (0.051) Egr1 (0.051) Klf7 (0.051) Gm397 (0.054) Klf12 (0.054) Klf15 (0.069) Klf14 (0.069) Zbtb7a (0.094) Rreb1 (0.100) Sp8 (0.100) Egr1 (0.102) | BP: transcription BP: negative regulation of transcription from RNA polymerase II promoter BP: protein amino acid phosphorylation MF: transcription activator activity MF: zinc ion binding |
| 21 | 49 |  |  | 0.053 | Klf14 (0.053) Smad3 (0.053) Zfp281 (0.053) Atf1 (0.053) Sp8 (0.054) Klf16 (0.058) Sp5 (0.058) Sp4 (0.058) Sp3 (0.061) Sp1 (0.068) Sp4 (0.082) Zfp148 (0.085) Creb1 (0.099) Klf13 (0.099) Wt1 (0.099) Jdp2 (0.099) Atf6 (0.099) Klf7 (0.099) Atf7 (0.123) Klf6 (0.137) Egr4 (0.149) Mbtps2 (0.167) Maz (0.176) Xbp1 (0.180) Mitf (0.187) | BP: negative regulation of transcription from RNA polymerase II promoter CC: transcription factor complex MF: transcription factor activity MF: ATP binding MF: zinc ion binding |
| 22 | 17 |  |  | 0.055 | Ctcf (0.055) Plag1 (0.640) | CC: plasma membrane MF: carbohydrate kinase activity MF: growth factor activity MF: cation channel activity MF: potassium ion binding |
| 23 | 28 |  |  | 0.070 | Lin54 (0.070) Irx2 (0.862) Irx5 (0.862) | BP: sensory perception of smell BP: G-protein coupled receptor protein signaling pathway BP: signal transduction BP: cell communication MF: olfactory receptor activity |
| 24 | 90 |  |  | 0.092 | Zfp161 (0.092) E2f1 (0.114) Mtf1 (0.114) Zfp161 (0.126) Zfp263 (0.157) Zfp128 (0.178) Hey1 (0.196) Sp2 (0.196) Wt1 (0.196) Sohlh2 (0.196) E2f3 (0.196) Tcfap2d (0.237) Egr4 (0.352) Hif3a (0.452) E2f4 (0.469) Tcfap2c (0.544) Zfp691 (0.544) Maz (0.544) Zfp524 (0.545) Esrra (0.545) Hes7 (0.545) Sp1 (0.603) Sp3 (0.625) Hes1 (0.625) Plag1 (0.625) Plagl1 (0.625) Smad1 (0.625) Zfx (0.625) Zfp691 (0.625) E2f6 (0.625) Ahr (0.625) Tcfap2b (0.625) Tcfap2a (0.625) Atf6 (0.625) Tcfap2e (0.625) Gm397 (0.625) Nhlh1 (0.625) Zbtb7a (0.625) E2F3 (0.625) Id2 (0.625) Tcfl5 (0.625) Zic4 (0.625) E2F3 (0.625) Gmeb1 (0.625) Zscan4c (0.625) Max (0.625) Mbd2 (0.625) Mnt (0.625) Gm98 (0.625) | BP: mRNA processing BP: RNA splicing MF: ATP binding MF: zinc ion binding MF: transcription activator activity |
| 25 | 32 |  |  | 0.101 | Sox12 (0.101) Sox4 (0.101) Sox3 (0.101) Sox21 (0.340) Srf (0.340) Mtf1 (0.340) Sox8 (0.340) Sox7 (0.340) Pou5f1 (0.340) Sox18 (0.340) Isgf3g (0.357) Sox11 (0.411) Sox7 (0.414) Foxa2 (0.448) Sox30 (0.448) Sox6 (0.456) Foxp4 (0.456) Onecut3 (0.456) Sox5 (0.467) Sry (0.467) Sox15 (0.467) Spib (0.467) Zfp105 (0.472) Irf7 (0.484) Irf3 (0.484) | BP: sensory perception of smell BP: G-protein coupled receptor protein signaling pathway BP: signal transduction MF: olfactory receptor activity MF: serotonin binding |
| 26 | 122 |  |  | 0.105 | Prdm11 (0.105) Stat1 (0.556) Zbtb1 (0.556) Sp100 (0.558) Sox21 (0.642) | BP: RNA splicing BP: mRNA processing MF: ATP binding MF: zinc ion binding MF: structural constituent of ribosome |
| 27 | 94 |  |  | 0.108 | Yy1 (0.108) Atoh1 (0.108) Ctcf (0.133) Mbtps2 (0.133) Tcf15 (0.133) Meis1 (0.529) Gm239 (0.719) Rarb (0.719) Gm98 (0.719) Pknox1 (0.885) | BP: transcription CC: ribosome MF: transcription factor activity MF: sequence-specific DNA binding MF: ATP binding |
| 28 | 6 |  |  | 0.111 | Gm397 (0.111) Mnt (0.140) Sohlh2 (0.148) Zfp128 (0.761) Hif3a (0.761) Arnt (0.761) Bhlhe40 (0.789) Nkx2-9 (0.789) Nkx3-1 (0.827) | BP: mRNA processing BP: RNA splicing MF: ATP binding MF: structural constituent of ribosome MF: ATP-dependent helicase activity |
| 29 | 47 |  |  | 0.129 | Gm239 (0.129) Six2 (0.352) Six6 (0.352) Zfp263 (0.352) Six1 (0.352) Six6 (0.352) Gm98 (0.352) Six3 (0.352) Six3 (0.405) Zbtb7b (0.568) Zbtb7c (0.568) Six1 (0.568) Six4 (0.568) Six2 (0.651) Egr4 (0.688) Creb3 (0.742) Plagl1 (0.786) Six6 (0.839) Osr2 (0.839) Zfp691 (0.839) | CC: extracellular region CC: intermediate filament CC: microtubule-based flagellum |
| 30 | 18 |  |  | 0.150 | Klf15 (0.150) Maz (0.150) Ctcfl (0.150) Zfp410 (0.150) Esr2 (0.150) Zfp281 (0.150) Zic5 (0.150) Zbtb7a (0.335) Insm1 (0.335) Zic2 (0.336) Tcf4 (0.498) Rest (0.521) Hic1 (0.521) Sp4 (0.521) Zbtb3 (0.521) Tcfap2c (0.521) Klf3 (0.521) Zfp740 (0.521) Esrra (0.521) E2f6 (0.521) Max (0.521) Nfia (0.521) Zbtb4 (0.521) Wt1 (0.521) Prkrir (0.521) Nfib (0.521) E2f4 (0.521) Mtf1 (0.521) Plagl1 (0.521) Sp3 (0.521) Gli3 (0.521) Gli1 (0.521) Tcfe2a (0.521) Smad1 (0.521) | BP: positive regulation of transcription from RNA polymerase II promoter BP: transcription BP: negative regulation of transcription from RNA polymerase II promoter BP: protein amino acid phosphorylation BP: potassium ion transport |
| 31 | 70 |  |  | 0.151 | Hmx1 (0.151) Hmx2 (0.151) Hmx2 (0.151) Hmx3 (0.151) Mnx1 (0.159) Foxa3 (0.208) Hmx1 (0.208) Barhl1 (0.208) Msx3 (0.208) Hoxa3 (0.208) Barx2 (0.208) Hmx3 (0.208) Barhl2 (0.240) Hoxa6 (0.284) Dlx2 (0.318) Hoxc13 (0.318) Hoxd8 (0.318) Nkx3-1 (0.318) Barhl2 (0.318) Hoxb8 (0.318) Dlx3 (0.318) ENSMUSG00000044690 (0.318) Irx5 (0.318) Nanog (0.318) Vsx2 (0.318) Hoxa5 (0.318) Dlx1 (0.318) Barx2 (0.318) Elf3 (0.318) | BP: sensory perception of smell BP: G-protein coupled receptor protein signaling pathway BP: signal transduction CC: integral to membrane MF: olfactory receptor activity |
| 32 | 57 |  |  | 0.182 | Hbp1 (0.182) Gcm1 (0.356) | BP: sensory perception of smell BP: G-protein coupled receptor protein signaling pathway BP: signal transduction BP: cell communication MF: olfactory receptor activity |
| 33 | 14 |  |  | 0.207 | Klf5 (0.207) Bcl6b (0.246) Sp3 (0.290) Sp1 (0.290) Klf7 (0.378) Klf4 (0.378) Klf6 (0.378) Klf7 (0.558) Klf8 (0.558) Klf2 (0.558) Plagl1 (0.558) Klf12 (0.558) Mbd2 (0.558) Zfp410 (0.558) Egr4 (0.558) Pbx3 (0.558) Smad3 (0.558) Wt1 (0.558) Eomes (0.558) Plagl1 (0.558) Klf16 (0.558) Klf13 (0.558) Smad2 (0.558) Hnf4a (0.558) Egr1 (0.558) Sp2 (0.558) Zfx (0.558) Mtf1 (0.558) Sp4 (0.558) Egr3 (0.558) Mafa (0.558) Ctcfl (0.558) | BP: negative regulation of transcription from RNA polymerase II promoter BP: Wnt receptor signaling pathway CC: transcription factor complex MF: ATP binding MF: zinc ion binding |
| 34 | 127 |  |  | 0.228 | Pknox1 (0.228) Grhl1 (0.228) Tcfcp2 (0.525) Dmbx1 (0.757) Cutl1 (0.757) Meis2 (0.757) Meis3 (0.757) Meis1 (0.757) Mrg1 (0.757) Id4 (0.757) Sp5 (0.757) Klf12 (0.757) Tgif2 (0.757) Pknox2 (0.819) Klf7 (0.827) Nr3c2 (0.852) Klf8 (0.852) | BP: cell division BP: RNA splicing CC: transcription factor complex MF: ATP binding MF: zinc ion binding |
| 35 | 38 |  |  | 0.236 | Myb (0.236) Mybl1 (0.445) Myb (0.445) | BP: sensory perception of smell BP: G-protein coupled receptor protein signaling pathway CC: integral to membrane CC: extracellular region MF: olfactory receptor activity |
| 36 | 110 |  |  | 0.251 | Ctcf (0.251) Ctcfl (0.251) | BP: negative regulation of transcription from RNA polymerase II promoter BP: mRNA processing CC: transcription factor complex MF: ATP binding MF: zinc ion binding |
| 37 | 31 |  |  | 0.258 | Atoh1 (0.258) Ctcfl (0.324) Yy1 (0.324) Tcfap4 (0.328) Mbtps2 (0.328) Smarcc2 (0.363) Neurod1 (0.440) Twist1 (0.440) Ctcf (0.440) Tcf3 (0.463) Rfx5 (0.560) Atoh8 (0.736) Figla (0.736) Tcfap2c (0.736) | BP: alcohol metabolic process BP: regulation of signal transduction CC: nucleus CC: endoplasmic reticulum MF: transcription factor activity |
| 38 | 0 |  |  | 0.273 | Plagl1 (0.273) Zfp128 (0.735) Six2 (0.828) | BP: sensory perception of smell BP: G-protein coupled receptor protein signaling pathway CC: integral to membrane CC: extracellular space MF: olfactory receptor activity |
| 39 | 115 |  |  | 0.277 | Ascl2 (0.277) | BP: immune response BP: signal transduction BP: cell adhesion CC: extracellular space MF: calcium ion binding |
| 40 | 86 |  |  | 0.287 | Klf15 (0.287) Tcfap2c (0.287) Tcfap2e (0.287) Tcfap2c (0.287) Tcfap2a (0.299) Tcfap2b (0.323) E2f4 (0.359) Zbtb49 (0.513) Tcfap2e (0.535) Smad3 (0.548) E2f6 (0.548) Tcfap2d (0.548) Tcfap2a (0.548) Egr3 (0.627) Elf1 (0.645) Ehf (0.835) ENSMUSG00000044690 (0.835) Gm5454 (0.835) Gm98 (0.835) Zfp263 (0.835) Sp3 (0.835) Etv4 (0.835) Klf5 (0.835) Tcfap2b (0.866) | BP: transcription CC: transcription factor complex MF: transcription factor activity MF: transcription activator activity MF: ATP binding |
| 41 | 2 |  |  | 0.323 | Myf6 (0.323) Hes7 (0.705) Myod1 (0.705) Ascl2 (0.705) Id4 (0.705) Tcfap2d (0.705) Smarcc2 (0.705) Hes1 (0.705) Myf6 (0.705) Zbtb7a (0.705) Ascl2 (0.705) Mafa (0.705) Nr2e3 (0.714) Atoh1 (0.714) Mesp2 (0.886) Tcf4 (0.886) | BP: transcription BP: inner ear morphogenesis MF: transcription factor activity MF: sequence-specific DNA binding MF: ATP binding |
| 42 | 125 |  |  | 0.335 | Nkx2-5 (0.335) | BP: innate immune response BP: regulation of production of small RNA involved in gene silencing by RNA CC: extracellular space MF: cytokine activity MF: cytokine receptor activity |
| 43 | 107 |  |  | 0.346 | Twist2 (0.346) Mycn (0.346) Atoh1 (0.346) Neurod2 (0.346) Nfib (0.346) Bhlhe22 (0.346) Tcf15 (0.346) Neurod1 (0.346) Bhlha15 (0.346) Max (0.353) Creb3l2 (0.353) Nr5a2 (0.353) Clock (0.353) Neurog1 (0.353) Tcfe2a (0.353) Bhlhe23 (0.353) Max (0.353) Mtf1 (0.353) Neurog3 (0.353) Trp63 (0.353) Myc (0.353) Neurog2 (0.353) Olig1 (0.353) Tal1 (0.353) Msc (0.353) Nr5a1 (0.353) | BP: G-protein coupled receptor protein signaling pathway BP: visual perception CC: integral to membrane CC: extracellular space MF: serine-type endopeptidase activity |
| 44 | 7 |  |  | 0.348 | Mlxip (0.348) Tcfap2c (0.665) Bhlhb2 (0.665) Zic3 (0.665) Mnt (0.665) Tcfe2a (0.665) Zic1 (0.665) Zic2 (0.665) Npas2 (0.665) Tcf4 (0.665) Creb3l2 (0.665) Tcfap2c (0.665) Max (0.665) Hey2 (0.758) | BP: transcription BP: cell development BP: apoptosis CC: mitochondrion MF: transcription factor activity |
| 45 | 16 |  |  | 0.382 | Foxl1 (0.382) | BP: sensory perception of smell BP: G-protein coupled receptor protein signaling pathway BP: signal transduction BP: cell communication MF: olfactory receptor activity |
| 46 | 82 |  |  | 0.382 | E2f1 (0.382) Smad3 (0.735) Nr2f2 (0.735) Sp3 (0.735) Tcfap2d (0.735) Egr4 (0.735) Sp1 (0.735) Plagl1 (0.735) Zbtb7a (0.735) Rfx6 (0.735) Klf6 (0.735) E2F2 (0.735) Sp100 (0.735) Irf6 (0.735) Egr1 (0.735) Zfp263 (0.735) Zfp148 (0.735) Zfp219 (0.735) Mbd2 (0.735) Zfp161 (0.735) Ehf (0.735) Tcfap2c (0.735) Wt1 (0.735) Zfp161 (0.735) Rorb (0.735) Plagl1 (0.735) Zbtb1 (0.735) Sim1 (0.735) Ctcfl (0.735) Zscan4c (0.735) E2f3 (0.735) Egr2 (0.735) Sp4 (0.735) Hivep2 (0.735) Klf5 (0.735) Tcfap2a (0.735) | BP: negative regulation of transcription from RNA polymerase II promoter CC: transcription factor complex MF: ATP binding MF: zinc ion binding MF: magnesium ion binding |
| 47 | 24 |  |  | 0.453 | Ebf1 (0.453) Tcfe2a (0.453) Twist1 (0.453) Trp63 (0.534) Zeb1 (0.575) | BP: regulation of transcription, DNA-dependent BP: organ morphogenesis MF: calcium ion binding MF: transcription factor activity MF: serine-type endopeptidase activity |
| 48 | 72 |  |  | 0.454 | Pou6f1 (0.454) Glis2 (0.454) Gata5 (0.773) | BP: G-protein coupled receptor protein signaling pathway BP: sensory perception of smell BP: signal transduction CC: integral to membrane MF: olfactory receptor activity |
| 49 | 22 |  |  | 0.460 | Hic1 (0.460) Gm397 (0.460) Smad4 (0.460) Klf1 (0.496) Zscan4c (0.496) Zscan4 (0.581) Smad1 (0.581) Klf2 (0.581) Gm397 (0.581) Hes7 (0.581) Zscan4 (0.581) Mtf1 (0.581) Gli2 (0.581) Zfp3 (0.581) Gli3 (0.581) Glis3 (0.720) | BP: defense response BP: response to external stimulus CC: anchored to membrane CC: plasma membrane part MF: calcium ion binding |
| 50 | 81 |  |  | 0.470 | E2f1 (0.470) Zbtb7a (0.470) Bcl6b (0.470) Wt1 (0.528) Klf6 (0.528) Sp3 (0.587) Zfp281 (0.706) Maz (0.706) Smad3 (0.706) Klf15 (0.706) Zfp219 (0.706) Max (0.712) Ctcf (0.712) Egr4 (0.712) Dlx5 (0.712) Zfp410 (0.712) Sp4 (0.712) Zfp740 (0.712) Ascl2 (0.712) E2f4 (0.715) E2f6 (0.746) E2f3 (0.765) Smarcc1 (0.765) Rreb1 (0.765) Sp2 (0.765) Zfp148 (0.765) Irf5 (0.765) Klf5 (0.765) Klf16 (0.765) Zfp281 (0.765) Zbtb4 (0.765) Zfp161 (0.765) | BP: transcription BP: protein amino acid phosphorylation MF: zinc ion binding MF: transcription activator activity MF: ATP binding |
| 51 | 51 |  |  | 0.484 | Zfp740 (0.484) Zfp202 (0.484) Mzf1 (0.484) Glis2 (0.484) Zfp281 (0.652) Prdm4 (0.755) Zbtb7b (0.859) Plagl1 (0.859) Egr1 (0.859) | BP: sensory perception of smell BP: G-protein coupled receptor protein signaling pathway BP: cell communication BP: signal transduction MF: olfactory receptor activity |
| 52 | 97 |  |  | 0.486 | Elf3 (0.486) Mef2a (0.486) Mef2c (0.486) Zfp105 (0.486) Ahctf1 (0.634) Phf21a (0.638) Foxp4 (0.638) Hoxb8 (0.670) Hoxa9 (0.670) Lcor (0.770) Onecut3 (0.770) Zfp957 (0.770) | BP: sensory perception of smell BP: G-protein coupled receptor protein signaling pathway BP: signal transduction BP: cell communication MF: olfactory receptor activity |
| 53 | 26 |  |  | 0.497 | Tcf4 (0.497) Zfp740 (0.497) Figla (0.497) Tcfe2a (0.497) Usf2 (0.497) Mlxip (0.497) Tbx18 (0.497) Mga (0.497) Sp4 (0.497) Atoh8 (0.497) Pax9 (0.497) Sp1 (0.497) Pax5 (0.497) Tbx15 (0.497) Sox13 (0.497) E2f3 (0.497) T (0.497) Creb3l2 (0.497) Mesp2 (0.497) Sp4 (0.497) Bhlhe41 (0.497) Pax1 (0.497) Klf12 (0.497) Sp5 (0.497) Tbx4 (0.497) Max (0.497) Twist1 (0.497) Srebf2 (0.497) Pax8 (0.497) ENSMUSG00000044690 (0.497) Snai2 (0.497) Eomes (0.497) Rbpj (0.497) Usf1 (0.497) Tcfe3 (0.497) Id2 (0.497) Tbx5 (0.497) Zfp161 (0.497) Klf7 (0.497) | BP: transcription MF: transcription factor activity MF: ATP binding MF: RNA binding MF: sequence-specific DNA binding |
| 54 | 71 |  |  | 0.555 | Pgr (0.555) Hoxc9 (0.555) Ppara (0.555) Nr2f1 (0.555) Rxrg (0.555) Nr2f2 (0.555) Rxrb (0.555) Zic5 (0.555) Rara (0.555) ENSMUSG00000044690 (0.555) Esr1 (0.562) Nr3c1 (0.604) Atoh8 (0.690) Zbtb7b (0.690) Nr2f6 (0.791) Nr2c1 (0.824) Tal1 (0.824) Zfp691 (0.824) Nr4a2 (0.824) Six4 (0.824) Esrra (0.824) Nr1d1 (0.824) Hoxa3 (0.824) | BP: defense response to bacterium BP: fatty acid metabolic process MF: cytokine activity MF: olfactory receptor activity MF: serine-type endopeptidase activity |
| 55 | 109 |  |  | 0.648 | Zbtb7b (0.648) Ctcfl (0.648) Obox2 (0.648) Klf6 (0.808) Ctcf (0.808) Glis2 (0.826) Obox3 (0.826) Zfp219 (0.826) Gli1 (0.826) Zfp3 (0.826) Plagl1 (0.826) Gli2 (0.826) Hsf1 (0.826) Plagl1 (0.826) Gli3 (0.826) Nkx2-5 (0.826) Zbtb7b (0.826) Nkx2-6 (0.826) Pou6f1 (0.826) Pgr (0.826) Nkx2-2 (0.826) Glis1 (0.826) Zscan10 (0.826) Egr1 (0.826) Tbx3 (0.826) Zbtb7a (0.826) Nkx2-4 (0.826) Tbr1 (0.826) Glis2 (0.826) Tcf12 (0.826) Mzf1 (0.826) Zic4 (0.826) Nkx3-1 (0.826) Pitx1 (0.826) Plag1 (0.826) Zbtb7c (0.826) | CC: keratin filament CC: proteinaceous extracellular matrix CC: high-density lipoprotein particle MF: calcium ion binding MF: sequence-specific DNA binding |
| 56 | 116 |  |  | 0.669 | Irf6 (0.669) Irf6 (0.669) | BP: transcription BP: positive regulation of transcription from RNA polymerase II promoter MF: transcription factor activity MF: sequence-specific DNA binding MF: ATP binding |
| 57 | 42 |  |  | 0.677 | Ctcf (0.677) | BP: defense response to bacterium BP: regulation of production of small RNA involved in gene silencing by RNA CC: extracellular space CC: external side of plasma membrane MF: chloride ion binding |
| 58 | 34 |  |  | 0.715 | Myb (0.715) Lhx8 (0.715) Lhx6 (0.715) Crebzf (0.715) Lhx6 (0.715) Mybl1 (0.831) Cxxc1 (0.831) | BP: cell division CC: mitochondrial inner membrane CC: spliceosomal complex MF: ATP binding MF: structural constituent of ribosome |
| 59 | 78 |  |  | 0.755 | Irx5 (0.755) Irx3 (0.755) | BP: sensory perception of smell BP: G-protein coupled receptor protein signaling pathway BP: signal transduction CC: integral to membrane MF: olfactory receptor activity |
| 60 | 101 |  |  | 0.757 | Irx3 (0.757) Irx3 (0.757) Irx3 (0.757) Irx5 (0.802) Trp53 (0.887) Mlxip (0.887) Myc (0.887) Dmrtc2 (0.887) | BP: sensory perception of smell BP: G-protein coupled receptor protein signaling pathway BP: signal transduction BP: cell communication MF: olfactory receptor activity |
| 61 | 5 |  |  | 0.758 | Ctcfl (0.758) Ctcf (0.758) Esr2 (0.758) | BP: transcription MF: ATP binding MF: RNA binding MF: transcription factor activity MF: sequence-specific DNA binding |
| 62 | 56 |  |  | 0.773 | E2f3 (0.773) Zfp148 (0.773) Smad1 (0.773) Bcl6 (0.773) Maz (0.773) Gata2 (0.773) | BP: positive regulation of transcription from RNA polymerase II promoter BP: chemotaxis BP: cell fate determination BP: anterior/posterior pattern formation MF: calcium ion binding |
| 63 | 3 |  |  | 0.802 | Bhlha15 (0.802) Twist1 (0.802) Twist2 (0.802) Tcf21 (0.802) Atoh8 (0.802) Mesp2 (0.802) Tbx19 (0.802) Zfp110 (0.802) Scrt2 (0.802) Olig3 (0.802) Olig1 (0.880) Neurog1 (0.880) Tal1 (0.880) Scrt1 (0.880) Neurog2 (0.897) | BP: sensory perception of smell BP: G-protein coupled receptor protein signaling pathway BP: signal transduction BP: cell communication MF: olfactory receptor activity |
| 64 | 98 |  |  | 0.808 | Zbtb7c (0.808) Rfx3 (0.808) Zbtb4 (0.808) Zfp524 (0.808) Esr2 (0.808) Rreb1 (0.808) Smad3 (0.808) Egr2 (0.814) Runx2 (0.833) Gli2 (0.865) Klf3 (0.865) Rfx7 (0.865) Egr3 (0.865) Zfp281 (0.865) | BP: transcription BP: positive regulation of transcription from RNA polymerase II promoter MF: transcription factor activity MF: sequence-specific DNA binding MF: zinc ion binding |
| 65 | 55 |  |  | 0.828 | Tcfap2d (0.828) | BP: transcription MF: transcription factor activity MF: sequence-specific DNA binding MF: ATP binding MF: zinc ion binding |
| 66 | 68 |  |  | 0.863 | Gm397 (0.863) Egr2 (0.863) Sox7 (0.863) | BP: sensory perception of smell BP: G-protein coupled receptor protein signaling pathway BP: signal transduction CC: extracellular space MF: olfactory receptor activity |
| 67 | 63 |  |  | 0.884 | Junb (0.884) Arid3b (0.884) Bach1 (0.884) Msx1 (0.884) Nr2e1 (0.884) Barhl1 (0.884) Fosb (0.884) Batf (0.884) Hoxd3 (0.884) Otp (0.884) Onecut1 (0.884) Dmrt3 (0.884) Hnf4a (0.884) Alx3 (0.884) Bsx (0.884) Sox1 (0.884) Jund (0.884) Vax1 (0.884) Nkx1-1 (0.884) Phf21a (0.884) Phox2b (0.884) Lbx2 (0.884) Jun (0.884) Cart1 (0.884) Isl2 (0.884) Lmx1b (0.884) Esx1 (0.884) Nfe2l1 (0.884) Nfe2l2 (0.884) Hoxb3 (0.884) Hoxa3 (0.884) Dmrta1 (0.884) Foxc2 (0.884) Hoxa1 (0.884) Pax6 (0.884) Nfe2 (0.884) En2 (0.884) Prop1 (0.884) Foxd4 (0.884) Foxa2 (0.884) Pax4 (0.884) Barhl2 (0.884) Dbx1 (0.884) Lhx1 (0.884) Hoxd1 (0.884) Lhx6 (0.884) Cdc5l (0.884) Fosl1 (0.884) Hoxd4 (0.884) Foxc1 (0.884) Mecp2 (0.884) Hoxa3 (0.884) Pou3f2 (0.884) Tead4 (0.884) Onecut2 (0.884) Dlx1 (0.884) Sox15 (0.884) Dbx2 (0.884) Foxb1 (0.884) Lhx3 (0.884) Hoxc6 (0.884) Hoxb7 (0.884) Pou3f4 (0.884) Rora (0.884) Pbx2 (0.884) Hoxd8 (0.884) Six6 (0.884) Pou3f1 (0.884) Otp (0.884) Lhx5 (0.884) Hoxa5 (0.884) Jdp2 (0.884) Nkx6-1 (0.884) Hoxa4 (0.884) Pou3f4 (0.884) Hoxa2 (0.884) Nkx1-2 (0.884) Hoxa7 (0.884) Esx1 (0.884) Tlx2 (0.884) Gsh2 (0.884) Msx3 (0.884) Hoxb5 (0.884) Ipf1 (0.884) En1 (0.884) Hoxc8 (0.884) Sox8 (0.884) Pou1f1 (0.884) Sox21 (0.884) Foxa1 (0.884) Pou1f1 (0.884) Zfp652 (0.884) Sox7 (0.884) Zfp187 (0.884) Lhx3 (0.884) Lhx5 (0.884) Foxa3 (0.884) Hoxa6 (0.884) Cdx1 (0.884) Prrx1 (0.884) Arx (0.884) Hoxb4 (0.884) Otx1 (0.884) Pou2f1 (0.884) Vdr (0.884) Hoxc4 (0.884) Lhx2 (0.884) Hoxc5 (0.884) Lef1 (0.884) Lhx1 (0.884) Pou3f1 (0.884) Nfix (0.884) Isx (0.884) Nkx6-3 (0.884) Vsx1 (0.884) Atf4 (0.884) | BP: sensory perception of smell BP: G-protein coupled receptor protein signaling pathway BP: signal transduction CC: integral to membrane MF: olfactory receptor activity |
| 68 | 4 |  |  | NA |  | CC: extracellular region CC: mitochondrial membrane MF: protein binding MF: calcium ion binding MF: kinase activity |
| 69 | 8 |  |  | NA |  | BP: sensory perception of smell BP: G-protein coupled receptor protein signaling pathway BP: signal transduction CC: integral to membrane MF: olfactory receptor activity |
| 70 | 10 |  |  | NA |  | BP: regulation of production of small RNA involved in gene silencing by RNA MF: calcium ion binding MF: serine-type endopeptidase activity MF: transcription factor activity MF: cation channel activity |
| 71 | 11 |  |  | NA |  | BP: complement activation, classical pathway BP: sensory perception of smell CC: extracellular space CC: external side of plasma membrane MF: olfactory receptor activity |
| 72 | 12 |  |  | NA |  | BP: sensory perception of smell BP: G-protein coupled receptor protein signaling pathway BP: signal transduction CC: integral to membrane MF: olfactory receptor activity |
| 73 | 13 |  |  | NA |  | BP: sensory perception of smell BP: G-protein coupled receptor protein signaling pathway BP: signal transduction BP: cell communication MF: olfactory receptor activity |
| 74 | 19 |  |  | NA |  | BP: sensory perception of smell BP: G-protein coupled receptor protein signaling pathway BP: signal transduction CC: integral to membrane MF: olfactory receptor activity |
| 75 | 20 |  |  | NA |  | BP: potassium ion transport MF: sequence-specific DNA binding MF: transcription factor activity MF: calcium ion binding MF: oxidoreductase activity, acting on the CH-NH2 group of donors, oxygen as acceptor |
| 76 | 21 |  |  | NA |  | BP: sensory perception of smell BP: G-protein coupled receptor protein signaling pathway BP: signal transduction BP: cell communication MF: olfactory receptor activity |
| 77 | 23 |  |  | NA |  | BP: G-protein coupled receptor protein signaling pathway BP: sensory perception of smell BP: signal transduction BP: cell communication MF: olfactory receptor activity |
| 78 | 25 |  |  | NA |  | BP: regulation of production of small RNA involved in gene silencing by RNA BP: cell adhesion BP: G-protein coupled receptor protein signaling pathway MF: cytokine activity MF: calcium ion binding |
| 79 | 29 |  |  | NA |  | BP: G-protein coupled receptor protein signaling pathway BP: sensory perception of smell BP: signal transduction CC: integral to membrane MF: olfactory receptor activity |
| 80 | 35 |  |  | NA |  | BP: transcription BP: protein amino acid phosphorylation MF: ATP binding MF: transcription factor activity MF: sequence-specific DNA binding |
| 81 | 37 |  |  | NA |  | BP: sensory perception of smell BP: G-protein coupled receptor protein signaling pathway BP: signal transduction BP: cell communication MF: olfactory receptor activity |
| 82 | 39 |  |  | NA |  | BP: defense response to bacterium BP: sensory perception of smell CC: external side of plasma membrane MF: calcium ion binding MF: serine-type endopeptidase inhibitor activity |
| 83 | 40 |  |  | NA |  | BP: sensory perception of smell BP: G-protein coupled receptor protein signaling pathway BP: signal transduction CC: integral to membrane MF: olfactory receptor activity |
| 84 | 41 |  |  | NA |  | BP: sensory perception of smell BP: G-protein coupled receptor protein signaling pathway BP: signal transduction CC: integral to membrane MF: olfactory receptor activity |
| 85 | 44 |  |  | NA |  | BP: sensory perception of smell BP: G-protein coupled receptor protein signaling pathway CC: integral to membrane CC: extracellular region MF: olfactory receptor activity |
| 86 | 48 |  |  | NA |  | BP: sensory perception of smell BP: G-protein coupled receptor protein signaling pathway BP: signal transduction BP: cell communication MF: olfactory receptor activity |
| 87 | 50 |  |  | NA |  | BP: monovalent inorganic cation transport BP: metal ion transport BP: protein kinase B signaling cascade CC: plasma membrane MF: calcium ion binding |
| 88 | 52 |  |  | NA |  | BP: sensory perception of smell BP: G-protein coupled receptor protein signaling pathway BP: signal transduction CC: integral to membrane MF: olfactory receptor activity |
| 89 | 54 |  |  | NA |  | BP: regulation of behavior BP: cellular ion homeostasis CC: extracellular space MF: calcium ion binding MF: serine-type endopeptidase activity |
| 90 | 58 |  |  | NA |  | BP: positive regulation of transcription from RNA polymerase II promoter CC: transcription factor complex MF: ATP binding MF: zinc ion binding MF: transcription activator activity |
| 91 | 59 |  |  | NA |  | BP: sensory perception of smell BP: signal transduction CC: extracellular space MF: olfactory receptor activity MF: cytokine activity |
| 92 | 61 |  |  | NA |  | BP: inflammatory response BP: response to external stimulus CC: extracellular space CC: proteinaceous extracellular matrix MF: triglyceride lipase activity |
| 93 | 64 |  |  | NA |  | BP: sensory perception of smell BP: G-protein coupled receptor protein signaling pathway BP: signal transduction CC: integral to membrane MF: olfactory receptor activity |
| 94 | 65 |  |  | NA |  | BP: sensory perception of smell BP: G-protein coupled receptor protein signaling pathway BP: signal transduction CC: integral to membrane MF: olfactory receptor activity |
| 95 | 66 |  |  | NA |  | BP: sensory perception of smell BP: G-protein coupled receptor protein signaling pathway CC: integral to membrane CC: extracellular region MF: olfactory receptor activity |
| 96 | 69 |  |  | NA |  | BP: immune response BP: cellular hormone metabolic process CC: integral to membrane CC: extracellular space CC: lysosome |
| 97 | 73 |  |  | NA |  | BP: rRNA processing BP: cell division CC: spliceosomal complex MF: ATP binding MF: structural constituent of ribosome |
| 98 | 74 |  |  | NA |  | BP: sensory perception of smell BP: G-protein coupled receptor protein signaling pathway BP: signal transduction BP: cell communication MF: olfactory receptor activity |
| 99 | 75 |  |  | NA |  | BP: sensory perception of smell BP: G-protein coupled receptor protein signaling pathway BP: signal transduction CC: integral to membrane MF: olfactory receptor activity |
| 100 | 76 |  |  | NA |  | BP: G-protein coupled receptor protein signaling pathway BP: sensory perception of smell BP: cell communication BP: defense response to bacterium MF: olfactory receptor activity |
| 101 | 77 |  |  | NA |  | BP: translation BP: protein folding MF: structural constituent of ribosome MF: RNA binding MF: ATP binding |
| 102 | 80 |  |  | NA |  | BP: transcription BP: protein amino acid phosphorylation MF: transcription factor activity MF: sequence-specific DNA binding MF: ATP binding |
| 103 | 83 |  |  | NA |  | BP: G-protein coupled receptor protein signaling pathway BP: defense response to bacterium CC: integral to membrane CC: extracellular space MF: calcium ion binding |
| 104 | 84 |  |  | NA |  | BP: mRNA processing BP: RNA splicing BP: negative regulation of transcription from RNA polymerase II promoter MF: transcription factor activity MF: ATP binding |
| 105 | 85 |  |  | NA |  | BP: sensory perception of smell BP: G-protein coupled receptor protein signaling pathway BP: signal transduction BP: cell communication MF: olfactory receptor activity |
| 106 | 87 |  |  | NA |  | BP: cell division BP: negative regulation of transcription from RNA polymerase II promoter CC: transcription factor complex MF: ATP binding MF: zinc ion binding |
| 107 | 88 |  |  | NA |  | BP: sensory perception of smell BP: G-protein coupled receptor protein signaling pathway BP: signal transduction BP: cell communication MF: olfactory receptor activity |
| 108 | 91 |  |  | NA |  | BP: rRNA processing BP: DNA repair BP: mitosis CC: spliceosomal complex MF: structural constituent of ribosome |
| 109 | 92 |  |  | NA |  | BP: sensory perception of smell BP: G-protein coupled receptor protein signaling pathway BP: signal transduction CC: integral to membrane MF: olfactory receptor activity |
| 110 | 93 |  |  | NA |  | BP: sensory perception of smell BP: G-protein coupled receptor protein signaling pathway BP: defense response to bacterium CC: integral to membrane MF: olfactory receptor activity |
| 111 | 95 |  |  | NA |  | BP: sensory perception of smell BP: G-protein coupled receptor protein signaling pathway BP: signal transduction CC: integral to membrane MF: olfactory receptor activity |
| 112 | 96 |  |  | NA |  | BP: G-protein coupled receptor protein signaling pathway BP: sensory perception of smell BP: signal transduction CC: integral to membrane MF: olfactory receptor activity |
| 113 | 99 |  |  | NA |  | CC: extracellular region CC: cytoplasm MF: binding MF: symporter activity MF: carboxylic acid transmembrane transporter activity |
| 114 | 100 |  |  | NA |  | BP: cell development BP: negative regulation of cell migration BP: striated muscle tissue development CC: plasma membrane MF: transcription factor activity |
| 115 | 102 |  |  | NA |  | BP: mRNA processing BP: RNA splicing BP: regulation of transcription MF: RNA binding MF: DNA binding |
| 116 | 103 |  |  | NA |  | BP: RNA splicing BP: mRNA processing BP: translation BP: cell cycle CC: nucleus |
| 117 | 104 |  |  | NA |  | BP: G-protein coupled receptor protein signaling pathway BP: sensory perception of smell BP: signal transduction BP: cell communication MF: olfactory receptor activity |
| 118 | 105 |  |  | NA |  | BP: transcription BP: protein amino acid phosphorylation MF: ATP binding MF: zinc ion binding MF: transcription activator activity |
| 119 | 106 |  |  | NA |  | BP: transcription BP: mRNA processing MF: ATP binding MF: zinc ion binding MF: magnesium ion binding |
| 120 | 108 |  |  | NA |  | BP: sensory perception of smell BP: G-protein coupled receptor protein signaling pathway BP: signal transduction CC: integral to membrane MF: olfactory receptor activity |
| 121 | 111 |  |  | NA |  | BP: transcription MF: transcription factor activity MF: sequence-specific DNA binding MF: ATP binding MF: zinc ion binding |
| 122 | 112 |  |  | NA |  | CC: extracellular region |
| 123 | 113 |  |  | NA |  | BP: mitosis CC: spliceosomal complex MF: ATP binding MF: zinc ion binding MF: translation regulator activity |
| 124 | 114 |  |  | NA |  | BP: sensory perception of smell BP: G-protein coupled receptor protein signaling pathway BP: signal transduction CC: integral to membrane MF: olfactory receptor activity |
| 125 | 119 |  |  | NA |  | BP: sensory perception of smell BP: G-protein coupled receptor protein signaling pathway BP: signal transduction CC: integral to membrane MF: olfactory receptor activity |
| 126 | 120 |  |  | NA |  | BP: sensory perception of smell BP: G-protein coupled receptor protein signaling pathway BP: defense response to bacterium CC: integral to membrane MF: olfactory receptor activity |
| 127 | 123 |  |  | NA |  | BP: G-protein coupled receptor protein signaling pathway BP: sensory perception of smell BP: signal transduction CC: integral to membrane MF: olfactory receptor activity |
| 128 | 124 |  |  | NA |  | BP: monovalent inorganic cation transport BP: lung epithelium development CC: protein complex CC: cell projection MF: serine-type peptidase activity |
